# Cross-Study Projections of Genomic Biomarkers: An Evaluation in Cancer Genomics

**DOI:** 10.1371/journal.pone.0004523

**Published:** 2009-02-19

**Authors:** Joseph E. Lucas, Carlos M. Carvalho, Julia Ling-Yu Chen, Jen-Tsan Chi, Mike West

**Affiliations:** 1 Institute for Genome Sciences and Policy, Duke University, Durham, North Carolina, United States of America; 2 Graduate School of Business, The University of Chicago, Chicago, Illinois, United States of America; 3 Department of Statistical Science, Duke University, Durham, North Carolina, United States of America; University of Pennsylvania School of Medicine, United States of America

## Abstract

Human disease studies using DNA microarrays in both clinical/observational and experimental/controlled studies are having increasing impact on our understanding of the complexity of human diseases. A fundamental concept is the use of gene expression as a “common currency” that links the results of *in vitro* controlled experiments to *in vivo* observational human studies. Many studies – in cancer and other diseases – have shown promise in using *in vitro* cell manipulations to improve understanding of *in vivo* biology, but experiments often simply fail to reflect the enormous phenotypic variation seen in human diseases. We address this with a framework and methods to dissect, enhance and extend the *in vivo* utility of *in vitro* derived gene expression signatures. From an experimentally defined gene expression signature we use statistical factor analysis to generate *multiple* quantitative factors in human cancer gene expression data. These factors retain their relationship to the original, one-dimensional *in vitro* signature but better describe the diversity of *in vivo* biology. In a breast cancer analysis, we show that factors can reflect fundamentally different biological processes linked to molecular and clinical features of human cancers, and that in combination they can improve prediction of clinical outcomes.

## Introduction

Microarray technology allows the capture of diverse aspects of genetic, environmental, oncogenic and other factors as reflected in global mRNA expression and opens the possibility of personalizing treatment of disease [1], [2]. Multiple studies have taken a “top-down” approach to profiling gene expression in human cancers, and this has led to the identification of tumor subtypes unrecognized previously as well as gene signatures predicting various clinical phenotypes [3]–[7]. Alternatively, other studies have taken a “bottom-up” approach to determine the change of gene expression caused by specific manipulations of cultured cells *in vitro*. In these studies gene expression serves as a common phenotype to recognize similar features in human cancers *in vivo* and to provide a direct linkage between the known biological perturbation and the clinical contexts [8]–[12].

Though many such studies have shown promise in using *in vitro* cell manipulations to understand *in vivo* biology, this approach cannot fully reflect the enormous phenotypic variation seen in human cancers. From such studies, one can derive *signatures*. These we define to be lists of genes that are differentially expressed along with their associated levels of differential expression (which we call weights). However, there is nearly always a poor match between these signatures and expression patterns of the same genes *in vivo*. Therefore, a conceptual framework is needed to further dissect, enhance and extend the *in vivo* utility of the *in vitro* derived signature. Here, we present a technique for achieving this purpose. We propose deriving multiple factors, based on human cancer gene expression studies, from an experimentally defined signature. These derived factors will retain their relationship to the original signature but represent distinct biological processes. Importantly, we show that different derived factors can be combined to provide much better predictive values for the clinical outcomes. Different factors also reflect different biological processes and are linked to various aspects of molecular and clinical features of human cancers.

There are a number of possible approaches to this problem. One popular approach has been to compare the identities of the differentially expressed probes to databases of pre-defined pathways. Descriptions of such approaches can be found in [13]–[15]. While these approaches are appealing for their interpretability, they rely on the appropriately pre-defined pathways rather than the structure of the data under study. Alternatively, one may simply define the signature activity level for a sample as the weighted average of *in vivo* expression levels (where the genes over which to compute the weights and the weights themselves are drawn from the original signature). Although some studies have shown the power of this concept, it is clear that one can not hope to capture the heterogeneity of *in vivo* biology from the one-dimensional controlled biological response the *in vitro* signature reflects.

The inherent heterogeneity of environment and cell type in tissue samples means that the genes in a signature may potentially involve many additional activities not evident *in vitro*. Further, experiments on cloned cell lines of a single cell type grown under tightly controlled conditions for a fixed (and relatively short) length of time may contrast starkly with clinical samples extracted from living organisms containing multiple cell types that have been in a dynamic environment for months or years. There is no clearly “correct” method for taking what is learned by microarray experiment in culture and applying it to assess pathway activity in tissue samples. Some genes may be poorer representatives of pathway activity *in vivo* because they are more likely to be involved in other pathways, because they react to environmental conditions that are not present *in vitro*, or for a myriad of other reasons. It is, therefore, important to provide a statistical and conceptual framework which can allow us to use the *in vivo* expression data to further dissect, refine and enhance the *in vitro*-derived gene signatures.

*Signature Factor Profiling Analysis* (SFPA), based on sparse statistical factor models, [16], [17] is a framework for mapping *in vitro* signatures to a collection of *in vivo* factors. While this sounds similar to hierarchical clustering (which has become the default method for this type of problem), there are important distinctions. First, while hierarchical clustering can be used to break a set of samples into groups, within which expression patterns are similar in some way, it does not quantify that similarity. Second, hierarchical clustering requires that each observation (gene) be a member of just one cluster. This precludes assigning clusters to biological pathways, because many combinations of pathway activity are possible. Lastly, because the factors are generated within a statistical model, it is possible to identify the levels of activity in each of the factors on a newly measured sample without redoing the statistical analysis. While there are techniques other than hierarchical clustering which address some of these issues, for example soft-clustering [18] and k-means clustering [19], our algorithm addresses them all within a single coherent statistical framework. SFPA provides:

- Robust statistical modeling of both experimental gene expression and tissue sample expression.
- Identification and correction of assay artifacts, which are known to be a significant issue associated with the use of microarray technologies.
- A mapping from a single signature, generated *in vitro*, to a collection of factors that retain the pertinent characteristics of the signature while better reflecting heterogeneity *in vivo* associated with the biological perturbation the signature represents.
- A model for imputing the values of factors in new collections of tissue samples even though these samples may originate from different groups and at different times.

We explore this analysis approach in translating a collection of gene signatures reflecting cellular response to five known tumor microenvironmental factors, discovered *in vitro* [8], with particular emphasis on the signature associated with response to lactic acidosis. We demonstrate that multiple factors arising in a breast cancer context remain representative of the individual microenvironmental pathway responses from which they are derived. Furthermore, these factors differentiate key biological phenotypes in breast cancer, are able to improve clinical predictions across multiple cancer data sets, and retain their predictive ability even when applied to samples taken at vastly different times or at different study centers.

## Results

### Context, Data and Analysis Strategy

We begin with five signatures defined by the transcriptional responses of cultured human mammary breast epithelial cells to five microenvironmental perturbations: hypoxia, lactic acidosis, hypoxia plus lactic acidosis, lactosis, and acidosis. Each of these is seen in human cancers and carries prognostic information with respect to clinical outcomes [8]. The signatures represent changes in expression of genes between a set of control observations and cells grown in the presence of lactic acidosis (25 mM lactic acid, pH 6.7), hypoxia (2% O2), lactic acid plus hypoxia, lactosis (25 mM sodium lactate, neutral pH), and acidosis (pH 6.7 without lactate). Expression assays used Affymetrix U133+ 2.0 microarrays and signatures reflecting each of the microenvironmental factors have been described [8]. As shown in [8], hypoxia, lactic acidosis and acidosis have strong prognostic significance in several studies of breast cancers. Our aim here is to explore the various components of the original gene signatures to evaluate the opportunity for further enhancing their prognostic values and dissecting them into distinct biological pathway-relevant factors with clinical relevance.

We use Bayesian Factor Regression Modeling (BFRM) [20] to define and estimate factors based on a given signature. This begins with a small collection of genes that are highly responsive to the original intervention (highly differentially expressed between control and experimental groups in cell culture) and then iteratively refines the gene set, based on co-expression in an in vivo data set, in the context of a statistical factor analysis. First, common patterns of expression (factors) are discovered within the subset of genes currently under consideration. Next, the association between these factors and the full set of genes on the array allows us to identify additional genes to be included in a revision of the factor analysis. The rationale for this is that, while evaluating factors underlying the initial selected signature genes allows us to elucidate *in vivo* variability that is not present *in vitro*, adding genes from outside the original signature can improve the characterization of these factors while providing linkages to other relevant pathways. Running SFPA on each of the five signatures independently, we obtain 11 hypoxia factors, 10 lactic acidosis factors, 20 hypoxia plus lactic acidosis factors, 17 lactosis factors and 9 acidosis factors. SFPA stops discovering factors once most of the variability in the original gene set has been explained.

### Signature-Factor Relationships

**Figure 1:**
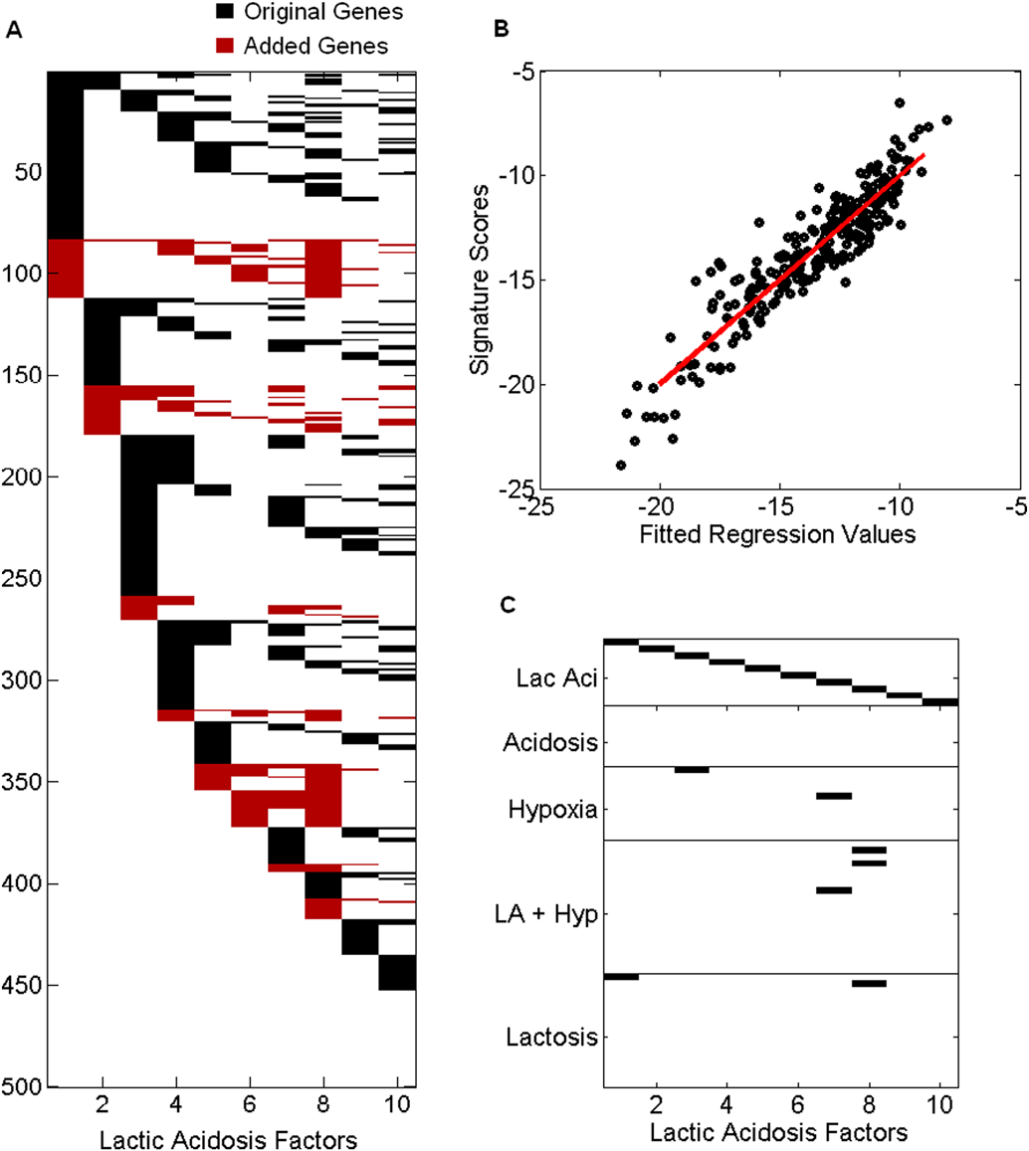
Factor associations. (a) Connections between genes and the 10 lactic acidosis factors in the statistical factor analysis of the breast cancer data from [21]. The genes include the initial selected signature genes (black) and those added through the iterative enrichment analysis (red), with black or red indicating that a gene (row) is highly associated with a factor (column), and white indicating little or no association. Cross-talk between putative pathway-related factors and genes is evident. (b) Lactic acidosis signature (vertical axis) is predicted by a linear regression fit (horizontal axis) on the seven factors significantly associated with the lactic acidosis signature. (c) Image of thresholded correlations between 67 factors (vertical) and the 10 lactic acidosis factors (horizontal), with black indicating pairs of factors whose pairwise sample correlation exceeds 0.9 in absolute value.

We will focus, for now, on the ten lactic acidosis factors. Examining the genes in each of the factors (Figure 1a) shows that all factors have representatives from the original signature in addition to genes added during the process of fitting the factor model. It is important to be sure that in the discovery of these ten factors, we have not lost our original signature. We check this by regressing the 10 sets of derived factor scores on the lactic acidosis signature scores. (Calculation of a signature score is described in the Methods section.) Witin a single multivariate regression model, we find that 7 of the 10 are significant at the .01 level, and that when we eliminate the remaining three factors from the multivariate regression, those seven remain significant. Thus, at least seven of the factors show a significant association to the original signature.

Figure 1b shows the fitted values from the regression of the lactic acidosis signature score on the lactic acidosis factors from the analysis of the 251 tumor sample data set from [21]. The 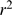 for this regression is high (.74), but it is possible these ten factors might be able to explain many different signatures. In order to show that this is not a spurious association, we test the hypothesis that this 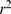 level is independent of which genes are assigned which weights. We re-sampled the weights 10,000 times, each time regressing the signature score vector computed from these weights on the 10 lactic acidosis factors and computing an 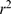 value. Of the 10,000 values of 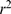 so computed under the null hypothesis, the maximum was .48 ensuring that the p-value ≪10^−4^. If we approximate the distribution of 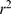 values by a beta distribution (calculated by method of moments) we get a very close fit (see Figure S1) and estimate the p-value to be ≈10^−13^. Because only the list of highly differentially expressed genes from the lactic acidosis signature, and not the weights, are used in the factor discovery, and because the weights are critical for the computation of the lactic acidosis signature scores, the ability to recover signature scores from factors is strong evidence of the relationship between the two.

The three factors derived from the lactic acidosis signature that were not important in the prediction of signature scores may still represent activity relevant to the presence of lactic acid, but they are not strongly predictive of the original signature. They may also simply represent the activity of biological pathways that involve very large sets of genes, and are thus discovered from many different possible starting points. Nonetheless, they represent significant structure in expression of the expanded signature gene set in tumor data, and none of these factors would be detectable from studying the signature alone as a phenotype.

Factors can reflect distinct aspects of biological activity. Figure 1c shows which of the 67 factors (all factors discovered from each of the five starting signatures) have high correlation with the 10 lactic acidosis factors from the Miller breast data analysis [21]. Notice that no two of the lactic acidosis factors are highly correlated, thus these factors seem to describe distinct processes. Some of the 10 factors, such as lactic acidosis factor 8 for example, are highly correlated with multiple other factors, indicating that these factors have been identified from multiple initial signatures. Most, however, show low levels of pairwise correlation. Among the 67 factors, 40 principal components are required to account for 95% of the observed variability (supplementary figure S2) implying that a relatively high biological “dimension” underlies the 67 factors – they reflect a diverse set of biological activities, and presumably pathways altered in the cellular responses to lactic acidosis within human breast tumors. Figure 1a shows the connections between genes and the 10 lactic acidosis factors in the analysis. The genes include the initial selected signature genes and those added through the iterative enrichment analysis. The SFPA-derived factors retain a high percentage of genes that have been shown to exhibit a change in expression when cells are exposed to the presence of lactic acid *in vitro*, showing in another way that these factors still maintain their connection with the original signature. The cross-talk between factors, in terms of genes defining more than one factor, is also evident.

### Factors Predict Molecular Features

SFPA-derived factors can represent distinct aspects of biological processes associated with clinical phenotypes. To evaluate this, we explored subset regression models to predict a number of clinical phenotypes in the Miller data set [21] - the phenotypes including ER and PgR status, p53 status and survival times. The molecular status indicators were modelled with binary probit regressions on the factors, and survival with standard Weibull survival models. We utilized the Shotgun Stochastic Search (SSS) method [22], [23] to identify small subsets of the factors showing predictive value with respect to each of these phenotypes. SSS is a variable selection model which allows the use of model averaging (based on posterior likelihood) for prediction. Model averaging has been shown to perform better than algorithms which use the single best model for prediction (such as AIC or BIC) because it gives a truer estimation of uncertainty [24]. This analysis was performed on the data set from [21], and then the resulting fitted/trained regression models were used to predict phenotypes in each of five separate and biologically diverse breast cancer data sets [25]–[28]. All data sets are available from the Gene Expression Omnibus (GEO).

#### Factors predict ER status

**Figure 2:**
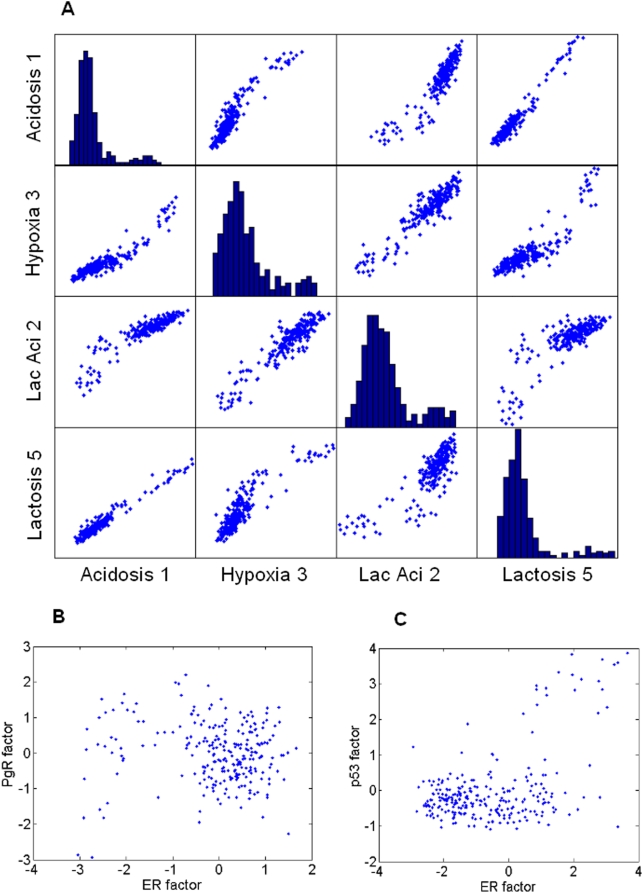
Estrogen receptor factor: Derivation and associaions. Each point in these plots represents a single patient from the dataset in [21]. (a) Pairwise scatterplots of factors Acidosis 1, Hypoxia 4, Lactic acidosis 2, and Lactosis 5 of the sixty-seven factors. Each of these factors is derived from a different starting signature and they are important and exchangeable in the prediction of ER status. The plots on the diagonal axis show histograms of the scores on the respective factors. (b) Three is no significant correlation between the ER and PgR factors. (c) The ER and p53 factors show some evidence of a relationship, but have clearly different structures (values shown are for activity of the respective factors in the data from [21]).

**Figure 3:**
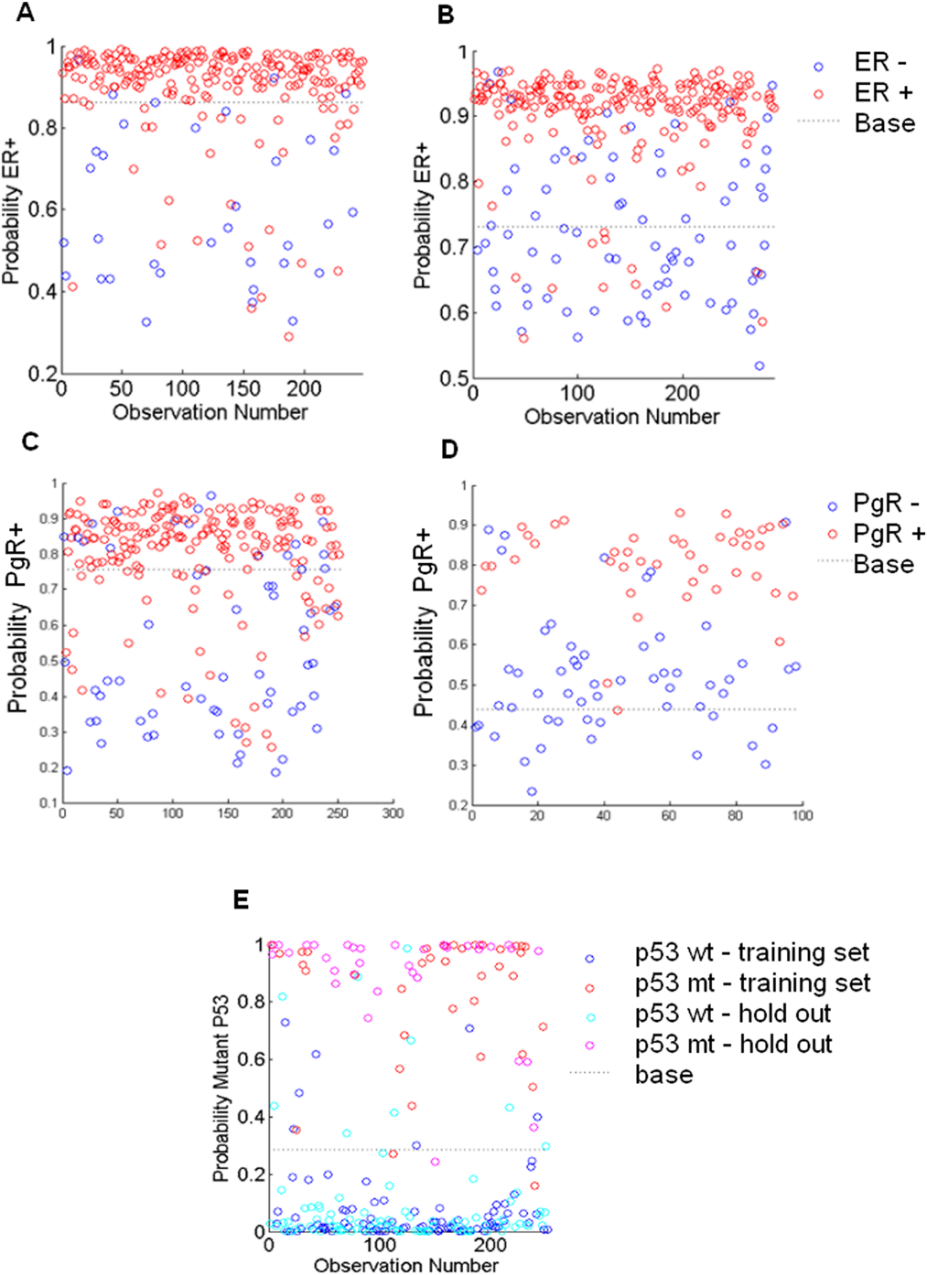
Factor – phenotype relationships. ER and PgR factors predict progesterone receptor status: (a) training data set [21]; (b) projected into the Wang data. Outcomes are PgR− (blue, obs = 0) and PgR+ (red, obs = 1). The ER factors (Acidosis 1, Hypoxia 4, Lactic Acidosis 2, or Lactosis 5): (c) training set [21], strongly associated with ER status; (d) projected into the tumor expression data from a completely different study – the Wang data set in this case 25 – are able to predict ER status. Outcomes are ER− (blue, obs = 0) and ER+ (red, obs = 1). (e) p53 status prediction, with outcomes p53 wild type (blues, obs = 0) and mutant (reds, obs = 1) split between training (dark blue and red) and test/validation (light blue and pink) samples.

The analysis indicates that highly scoring regression models for the prediction of ER status utilize one of the factors – Acidosis 1, Hypoxia 4, Lactic Acidosis 2, or Lactosis 5. From Figure 2a, one can see that the correlation between any two of these factors is high, so we will refer to them collectively as the ER factors. Figure 3a demonstrates the ability of this factor to predict ER status on the training set [21] and 3b shows prediction on a distinct and completely unrelated test set [27]. To examine the gene ontology (GO) composition of the list of genes involved in the ER factors, we applied the GATHER analysis [29] and find that GO terms associated with cell cycle, proliferation and and mitosis are greatly enriched in these factors (Table 1), corroborating well-known connection between cell progression and ER. It is also expected that the presence of lactic acid or hypoxia acts to shut down the cell cycle and the ER factor appears to directly link the two processes.

**Table 1:** Gene Ontology of the ER Factor.

Gene Ontology	# Genes	p-value	Bayes Factor
Cell Cycle	34	<.0001	28
Cell Proliferation	39	<.0001	25
Regulation of cell cycle	21	<.0001	17
Mitotic cell cycle	15	<.0001	16

We use GATHER to identify the collection of probes that have >99% probability of inclusion in the ER factor. There is, not surprisingly, a high level of enrichment of cell cycle genes in this factor. Bayes Factors and p-values are reported by GATHER, see [29] for details.

#### Factors predict PgR status

Estrogen and progesterone are known to be antagonists, so it is expected that ER factors can predict progesterone receptor status. Using SSS we find that the highly scoring regression models for PgR status involve the ER factor in addition to Lactic Acidosis factor 10 – we label this the PgR specific factor. Figures 3c and 3d show the fitted and predictive ability of these two factors used in a binary regression model fit to progesterone receptor status. There is no significant correlation in tumor expression between the PgR and ER factors (Figure 2b). Gene ontology for the genes in the PgR specific factor (Table 2) bear out some of the known links between progesterone and RNA metabolism in breast cancer [30].

**Table 2:** Gene Ontology of the PgR Factor.

Gene Ontology	# Genes	p-value	Bayes Factor
Nucleotide Metabolism	6	.0004	4
RNA Processing	8	.0008	4
RNA Splicing	5	.003	2
Nulcear mRNA splicing	5	.003	2
RNA metabolism	8	.003	2

The gene ontology from GATHER for the probes with >99% probability of inclusion in the PgR factor.

#### Factors predict p53 status

The third binary phenotype, wild type versus mutant p53 gene, is present in only the data set from [21]. SFPA was re-run on a randomly selected 50% of these data and used to predict the other 50% (Figure 3). Highly scored models for p53 involve the ER factor, the PgR specific factor, and one of either Hypoxia 1 or Lactic Acidosis 3. The correlation between these latter two factors is 99%, so we label them collectively as the p53 specific factor. Gene ontology for this factor is identical to that for the ER factor with the exceptions that “cell proliferation” and “DNA replication initiation” are replaced by “nuclear division” and “M phase”. For all gene ontologies listed in the top eight for these two factors, the Bayes factors are ≥10. Because of the high degree of similarity in the gene ontology, it is tempting to try to equate these two factors. Figure 2c shows a scatterplot of the activity of the tumors in the data from [21] on each of the two factors. The p53 factor is significantly bimodal, and the mild correlation one can see is due entirely to this bimodality, as tumor samples with high ER factor activity are more likely to be in the second mode of the p53 factor. We theorize that this bimodality is associated with a particular subtype of the p53 mutation. However, there is no evidence of multimodality in the ER factor, and the p53 specific factor predicts ER status poorly. Because of these differences, and because cell replication is a complex process, it is likely that these two factors are related to distinct features of cell development.

We stress that, if we restrain ourselves to considering the original *in vitro* lactic acidosis signature, we have no ability to fit or predict any of these biological phenotypes (Table 3). Additionally, these factors were generated entirely without regard to the ER status, PgR status, or p53 status of the samples. This is in contrast to a more typical design in which signatures associated with phenotypes are defined strictly based on genes with expression profiles that match those phenotypes (for example [21]). This type of design is plagued with difficulties that arise from the large number of genes, out of the tens of thousands on an array, with expression patterns that match any arbitrary phenotype. With SFPA, we search for genes that are expressed together without regard to phenotype, and we are therefore much less likely to be plagued by false discovery (as proven by our out of sample predictive accuracy).

**Table 3:** Phenotype Associations with Factors and Signature.

	LA Factors		LA Signature	
	P53 Mutant	P53 Wild	P53 Mutant	P53 Wild
>50%	63	5	23	10
<50%	9	174	49	169
	ER+	ER−	ER+	ER−
>50%	202	17	212	31
<50%	11	17	1	3
	PgR+	PgR−	PgR+	PgR−
>50%	180	33	185	54
<50%	10	28	5	7

Cross tabulation for prediction of three pheontypes by lactic acidosis factors (left) and by the lactic acidosis signature (right). There is a mild correspondence between lactic acidosis signature score and P53 status, while the best model for predicting ER or PgR status from the lactic acidosis signature involves assuming that all samples are positive (essentially the null model).

### Factors Predict Clinical Phenotypes

SFPA offers a technique for interrogating a single independent tumor sample against any number of biologically determined signatures, and then consequent linking of factors to phenotypes may include clinically relevant outcomes such as patient survival outcomes and drug response.

#### Factors improve prediction of breast cancer survival

**Figure 4:**
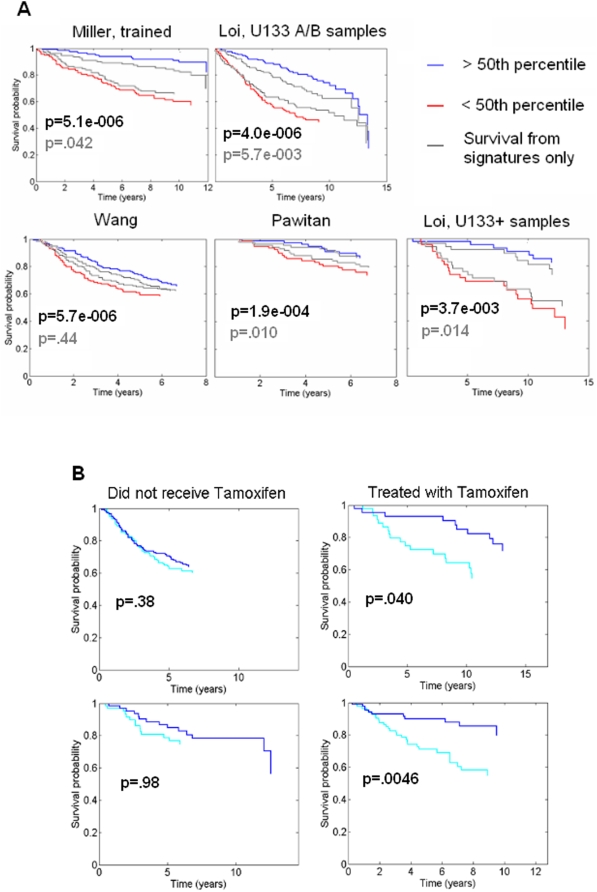
Predicting survival and drug response. (a) Predicted survival times from an average of Weibull survival models where used to split the 251 samples from [21] according to above/below median predictions, and the resulting empirical survival curves (Kaplan Meier curves) are shown. The red/blue stratification of patients is from the analysis using subsets of the 67 factors (red - high risk 50%, blue low risk 50%); the grey curves are from the same analysis using all of the original five signatures (thus there is no compensation for over-fitting here). The p-values in each of the plots correspond to stratification by factor analysis (top, black) and stratification using the signatures (bottom, grey). Data from [21] was used to identify the survival models, therefore this plot represents fitted values. The four additional plots represent prediction in the four different breast tumor samples based on the analysis of only the training data. The predictive relevance, and importance, of the factors is evident and consistent across studies, and consistently improves on that achieved by use of signatures alone. (b) The first Lactic Acidosis factor predicts survival in patients who were treated with Tamoxifen (left half), but shows no predictive value in patients who did not receive the drug (right half). In all of these figures, p-values represent significance in a cox proportional hazards model.

Subsets of the 67 factors were evaluated in Weibull survival regression models using the SSS method to identify and score models predicting survival. Each model in a resulting set of highly scoring models produces fitted survival curves and also may be used to predict survival for new samples. Bayesian analysis mandates averaging predictions from such a set of models, and this was done to result in Figure 4a. This shows fits of survival curves for the training data set [21], together with out of sample predictions in four of the other data sets for which information regarding survival exists. Recall that these are data sets from quite distinct and diverse studies, so we are assessing a model fitted to one data set on four quite challenging out of sample validation data sets. Though not described further here, the BFRM statistical model analysis used by the SFPA also addresses issues of gene-sample-study specific effects within the analysis and is able to correct enough of the idiosyncracies and bias inherent in microarray assays to retain predictive accuracy [19], [31]. The results demonstrate that the factorprofiles of these *in vitro* environmental signatures can improve survival prediction significantly across several test data sets. Similar results are obtained for the prediction of metastasis-free survival.

#### Factors predict Tamoxifen response

Four of the breast cancer data sets have clinical annotation pertaining to treatment with Tamoxifen. Though the 67 factors are in no way specifically targeted at Tamoxifen, we do know they are associated with relevant biological pathways. From our 67 factors, we found that Lactic Acidosis 1 is predictive of Tamoxifen resistance. It differentiates metastasis-free survival in patients who received the drug and shows no predictive ability in patients who did not (Figure 4b; the analysis underlying this followed the same approach as for survival discussed above). Because all of the patients who received Tamoxifen were ER positive, drug resistance associated with this factor must be independent of the antagonistic action of the drug on estrogen receptors. Since none of these data sets were used in the training of the factor model, the ability of these factors to distinguish resistance to Tamoxifen is remarkable and demonstrates that they are robust to the collection biases often seen in microarray experiments. We again used GATHER to study the ontology of the genes included in this factor (Table 4). This connects with the known association of Tamoxifen with phosphate transport [32], [33] as well as cell adhesion [34], [35]. In particular, Cowell et al. report that p130Cas/BCAR1 is a cell adhesion molecule that promotes resistance to Tamoxifen via a particular phosphorylation pathway. In addition to these connections to the secondary effects of Tamoxifen is the well-known connection between survival of patients on Tamoxifen and toxicity associated with blood coagulation [36]. Further study of the genes in this factor may lead to insight into the mechanism behind Tamoxifen resistance in ER positive breast cancer.

**Table 4:** Gene Ontology of the Tamoxifen Factor.

Gene Ontology	# Genes	p-value	Bayes factor
Phosphate transport	6	<.0001	8
Inorganic anion transport	6	.0002	5
Cell adhesion	11	.0002	5
Anion transport	6	.0003	4
Response to abiotic stimulus	8	.0008	4
Response to external stimulus	15	.001	3
Blood coagulation	4	.002	3

The gene ontology from GATHER for the probes with >99% probability of inclusion in the Tamoxifen specific factor.

#### Discovery of organ-specific factors from lactic acidosis signatures

While the same biological processes may contribute to tumor phenotypes in different cancers, the process by which this happens may be entirely different given the particular cellular context, tissue-specific gene expression and epigenetic influences. Since SFPA can utilize *in vivo* cancer gene expression to dissect the *in vitro*-generated gene signature, it offers the possibility of identifying tissue and organ-specific factors associated with the same gene signatures. This application has the potential to distinguish sub-pathways that are conserved across many tissue types from those that are organ-specific. To illustrate this point, we utilize the lung cancer data set published in [11] and the ovarian cancer data set from [10]. We obtained the lung cancer data from GEO and the ovarian cancer data from the Duke Integrative Cancer Biology Program (ICBP) web site (http://data.cgt.duke.edu/platinum.php). We performed SFPA analysis of the same lactic acidosis signature as a starting point for factor discovery from the lung and ovarian cancer data sets.

In the case of the lung cancer, the analysis discovered 20 factors associated with lactic acidosis. When we compared the expression levels of the 10 lactic acidosis factors in the breast cancer data with the 20 lactic acidosis factors discovered in the lung cancer data, we found that several factors are highly conserved, including the tamoxifen factor, the p53 specific factor, as well as factors 7 and 8. In contrast, the ER and PgR factors are only found in breast cancers. If we look specifically at standardized raw expression levels for the genes in the ER factor in the breast data (figure S3a) as compared to that for the lung data (figure S3b) we see that there is consistent variation in the breast data which is not present in the lung data. In contrast, the standardized raw expression for the conserved tamoxifen factor shows a coherent expression pattern in both breast (Figure S3c) and the lung cancers (Figure S3d). Additionally, within this data set, our newly discovered factors also possess significant prognostic value, being able to distinguish between the two types of cancers (Figure S4a) as well as distinguish between high and low risk patients (Figure S4b). Similar observations are also present in ovarian cancer since the model averaged survival using the 8 lactic acidosis factors discovered in the ovarian cancer dataset can clearly differentiate high versus low acuity patients (Figure S4c). Additionally, we see the same patterns of loss of the ER factor (Figure S5(a,b)) and conservation of the tamoxifen factor (Figure S5(c,d)). Finally, we find that the exact same three factors, the p53 specific factor, factor 7, and factor 8, have analogous factors in the ovarian cancer data with greater than 90% correlation (as computed on the 251 breast cancer samples).

## Discussion

It is increasingly common for investigators to use gene expression signatures directly as phenotypes to link various biological processes and perturbations to disease phenotypes and chemical agents. Although these signatures derived *in vitro* offer a way to understand the *in vivo* biology, there is still considerable limitation due to the differences between these two settings. Here, we have exemplified a statistical approach to further improve the *in vitro* gene signatures based on the gene expression in *in vivo* cancer samples. Elaborating the factor profile underlying the original signatures can, as we have seen, improve the *in vivo* relevance by more fully describing the diversity of *in vivo* expression patterns. This may enhance prognostic value and provide mechanistic insights into how biological processes affect clinical phenotypes. As an example, we have found direct links between factors generated by the use of SFPA on the lactic acidosis signature. Such links are intriguing, and open questions regarding causation as well as questions about the biological associations of the remaining factors. However, regardless of links to known biology, this strategy and analysis seem to provide an advance in our ability to obtain consistent results across many different data sets collected at different times by different groups. This is a significant advance, as data collection inconsistencies are one of the main roadblocks to the use of microarrays in a clinical setting.

There are several possible explanations for the enhancement of the prognostic values achieved with SFPA. It is possible that certain genes or pathway components in the original gene signatures are simply noise or artifact due to their *in vitro* origins. These genes may offer no or even negative prognostic values for *in vivo* biology. By using SFPA to separate different components, it is possible to enhance the prognostic value by selecting only the relevant components or genes for predictive purposes. By so doing, it is also possible to examine the genes comprising those factors with strong links to clinical phenotypes which will lead to biological insights into this association.

Another opportunity this analysis raises is the ability to uncover the pathways which would be “hidden” in the *in vitro* signature. In our current study, factor one was not immediately recognizable as a clinically relevant list of genes, but the ability of this factor to predict patient resistance to Tamoxifen points to an important connection which would not have been possible to discern otherwise. This observation will lead to efforts in investigating the biological roles of this factor and how it is related to Tamoxifen treatment and cellular response to lactic acidosis. For example, it is well known that tumor hypoxia negatively impacts clinical outcomes, but the actual mechanism by which this occurs is complex and may include radiation resistance, increased tumor invasion, migration, increased survival and decreased drug sensitivities [37]. Although these hypoxia-induced effects occur in cancer patients, many of these events cannot be replicated or modeled in any particular *in vitro* setting. It is possible to uncover these processes via of the use of SFPA for the cancer gene expression. In a similar fashion, it is unclear how lactic acidosis responses are linked to good prognosis [8], and SFPA will allow us to explore *in vivo* gene expression to dissect this response and develop testable biological hypotheses. Equally importantly, the mechanisms by which hypoxia and lactic acidosis link to different clinical outcomes may vary among different cancer types, and the use of SFPA can specifically pinpoint the relevant biological processes to target or intervene to modulate clinical courses of cancer patients.

Tremendous resources continue to be expended on the discovery of biomarkers for drug susceptibility. The ability to predict susceptibility to a given drug has the potential to significantly increase efficacy while decreasing morbidity and mortality in the relevant patient population. Additionally, it opens the possibility of facilitating the process of bringing new drugs to market. We have demonstrated the efficacy of SFPA for translating signatures discovered *in vitro* into factors which are clearly related to specific biological processes and which can be used to assess important clinical outcomes. The factors may be applied to just one observation (an important consideration for use in a clinical setting), and remain consistent across many different data sets. We do view this as a useful step ahead in thinking about how gene expression genomics will advance us towards the goals of personalized medicine.

## Methods

A total of five signatures were derived from two different experiments on Human Mammary Epithelial Cells (HMEC). The details of the collection of gene expression data from these cell lines are in [8]. Signatures from these experiments were derived using the Bayesian Factor Regression Modeling (BFRM) software detailed in [19], and that has been used in multiple previous analyses of similar data [31], [38]. The workspaces used for BFRM are available in the supplementary materials S1, S2, and the software is publicly available [20].

In designed experiments such as [8], BFRM provides a sparse ANOVA framework for studying changes associated with environmental stresses. It includes functionality for correcting systematic laboratory bias which come about due to differences in conditions under which the microarray data is collected. These systematic differences are reflected in the doping control genes, which are used to construct the correction factors.

### Sparse Regression for Experiments with Known Variation

BFRM is a Bayesian modeling framework. As such, we assume that all of the parameters of our model are random variables. In order to learn more about the values of these parameters, we specify prior distributions, which are subsequently updated based on the data. The result of fitting the model to data in this way is a joint posterior distribution for all of the model parameters. In our case, the parameters of interest are the coefficients of the regression.

The general model implemented in BFRM is as follows. Let 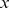 be a matrix of expression values where (row 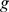, column 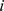) 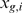 is the expression of gene 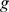 from sample 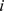 where 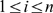. Denote the design matrix (describing known sources of variability) by 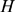 having elements 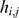 on sample 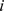 and design or regression variable 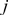. The model may be written as a separate linear regression for each probe on the array:
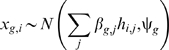



Or alternatively in matrix notation
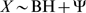
where 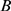 is a matrix of regression coefficients having elements 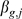 and 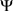 is a diagonal covariance matrix with non-zero diagonal elements 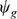. We allow the regression coefficients to vary across both genes and design vectors ,but assign them a sparsity prior, 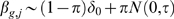. We define 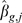 to be the posterior mean for 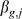, 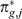 to be the posterior probabilities on non-zero values of the 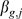, and 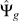 to be the posterior mean of 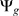. All of these parameters are computed automatically by BFRM (along with many others).

We have used a prior distribution for the coefficients of the regression that has a point mass at zero. This reflects our belief that, for any particular intervention, there will be relatively few genes (of the over ten thousand that are measured in a microarray experiment) that are affected. For the case outlined in this paper, we argue that growing mammary epithelial cells in the presence of mild lactic acidosis has led to changes in the expression of some of the genes on the array, but that most remain unchanged. Thus our posterior distribution for each 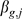 will consist of a probability that the parameter is non-zero (corresponding to a probability that the gene is differentially expressed in the lactic acidosis experimental group versus the control group), along with a distribution on the magnitude of that possible change.

The prior on 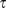 is assumed to be a diffuse inverse gamma distribution (which is a standard conjugate prior), and the prior on 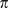 is also given a point mass mixture prior, reflecting the belief that we must maintain significant mass around the extremes (zero and one) even after updating with all of the probes on the chip (50,000+). The precise values of all hyper-parameters are available in the parameter files in the supplementary section.

We define a signature to be a list of genes and associated weights. Using the posterior parameters from above we define the weight of gene 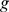 for experimental group (design variable) 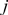 to be 
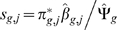
. Calculation of the level of activity of a known signature within a tumor sample requires that we initially subtract mean expression levels and laboratory biases. These are computed with BFRM exactly as above with the exception that the design matrix contains only the intercept vector and correction factors (no design vectors). If 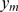 is a *p*-dimensional vector of corrected expression values associated with tumor sample *m*, then the signature score of signature 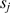 in sample *m* is the weighted average 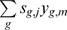.

### Analysis of Breast Cancer Data Sets

We use six cancer data sets with Affymetrix U133+ expression samples available on the Gene Expression Omnibus (GEO) web site. Details of the collection and measuring are contained in [21], [25]–[28]. For all but Wang, Affymetrix .CEL files were available, and we computed RMA normalized values in these cases. For the data set from [27], we used MAS5 normalized data which was obtained from the authors.

Statistical factor analysis using BFRM estimates latent factors that represent common, underlying aspects of covariation of subsets of genes, typically representing expression gene-by-gene in terms of contributions from possibly several factors. The iterative analysis to expand on an initial set of signature genes that we used here then revises the gene list by adding in genes apparently associated with estimated factors, and then refitting the model. Full details of this algorithm are available in [19], [20]. To choose a collection of seed genes associated with experimental group *j*, we identify all genes, *g*, such that 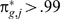. From this list of genes, we take the 25% with the highest absolute change in expression level between the control group and the corresponding experimental group (as measured by the posterior 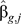). Following this procedure, we obtain between 20 and 200 “seed” genes for factor analysis.

Given a signature, we must choose a collection of tissue samples on which to train the factor model. Because of its relatively large size, the availability of CEL files, and the wealth of clinical and phenotypic information, we chose the data set from [21] for the identification of factors. We have five sets of seed genes, obtained as described above, from experiments on HMEC's. For each of these five sets of genes, we independently use BFRM to obtain the factors that are represented. We limited the number of genes to recruit into factors to a total of 500.

To fit our binary regression and survival models, statistical analysis used Shotgun Stochastic Search (SSS) routines from [22], [23]. Initialization files used by SSS for these searches are included in the supplementary materials S1, S2. All Kaplan-Meier curves showing differential survival are drawn by separating samples at the median of the score that is relevant for that figure.

### Statistical Factor Models for Tumor Expression Data

Factor models are structured as in [16]. If 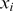 represents the column vector of gene expression measures on *p* genes as assayed in a single tumor, 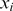 is regressed linearly on a combination of an overall intercept term and assay correction factor, plus a set of latent (i.e., to be estimated) factors. If 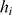 is the column vector of known regressors (the intercept and assay correction terms) on tumor *i*, the model is of the form
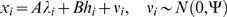
where 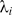 is a column vector of unknown latent factor values on tumor *i*, and *A*,*B* are coefficient matrices. In the BFRM context, both *A* and *B* are large “tall and skinny” matrices with many more rows (genes) than columns (the number of regressors and factors), and are described by the same sparsity probability models introduced above for the elements of 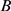 (inducing many zeros).

Implicit in this formulation is the assumption that there is a set of vectors, equivalent to design vectors, which describe some part of the variation observed in the matrix of expression values, 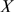. This leads to the grouping of probes in a way that is comparable to clustering, whereby we assign genes corresponding to non-zero values in any particular column of 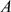 to the same group. This is exactly parallel to sparsity in the coefficients associated with the design vectors in that we are assuming that most genes are not differentially expressed with any single latent factor.

Calculation of the activity of a set of factors, 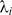 on each tumor *i*, and estimation of the factor loadings, 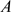, is then a problem of statistical estimation of the overall model. Details of these calculations are available in [39]. The issue of projecting factors to a new sample, 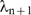, is then one of prediction that is immediately available from the BFRM analysis framework [19], [20]. For completeness, we present the formula here:
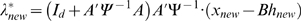



Where 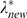 are approximations of the factor scores for a new observation, with gene expression values 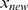 and design variables 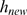.

## Supporting Information

Figure S1 We computed the significance of the relationship between the lactic acidosis factors and the lactic acidosis signature by resampling the lactic acidosis signature weights and modeling the resulting scores with the factors. After 10,000 iterations, we fit the sampled r-squared values to a beta distribution. This figure shows a Q-Q plot of the distribution of resampled values versus the best fit beta distribution. Using this beta distribution, we find that the r-squared value from regressing the true signature scores on the factors is significant with p-value approximately 1e-13.
(0.03 MB JPG)

Figure S2 Percent of variation across all discovered factors as a function of the number of principal components used.
(0.01 MB PNG)

Figure S3 Figures (a) and (b) show the expression levels of the probes from the ER factor (discovered in breast tissue). (a) shows a conserved pattern of expression in the breast samples that is lost in the lung samples (b). (c) and (d) show the same figure, but for probes from the Tamoxifen susceptibility factor. For purposes of visualization, samples are sorted such that the first principal component is increasing. In figures (a) and (c) the rows are sorted according to increasing correlation with the first principal component. The ordering of the rows in figures (b) and (d) is forced to be the same as that in (a) and (c) respectively.
(0.64 MB PNG)

Figure S4 Lactic acidosis factors discovered in lung cancer can distinguish between adenocarcinoma and squamous cell carcinoma (a) as well as stratify patients according to rates of recurrence (b). Factors discovered in ovarian cancer have similar prognostic ability (c).
(0.05 MB PNG)

Figure S5 As in figure s3, probes show a consistent expression pattern in breast cancer that is missing in the ovarian cancer data set (a) and (b) while the tamoxifen susceptibility factor is conserved across the two data sets.
(0.48 MB PNG)

Supplemental Materials S1 High dimensional sparse factor modeling: Applications in gene expression gneomics. Reference 17 is currently in press, so we have included it as supplementary material.
(3.27 MB PDF)

Supplemental Materials S2 In-vitro to in-vivo factor profiling in expression genomics. Reference 37 is currently in press, so we have included it as supplimentary material.
(0.73 MB PDF)
