# Supplementary figures and images for: Cross-Study Projections of Genomic Biomarkers: An Evaluation in Cancer Genomics
Source: PLoS One. 2009 Feb 19;4(2):e4523. doi: 10.1371/journal.pone.0004523 (PMC2638006; doi:10.1371/journal.pone.0004523)

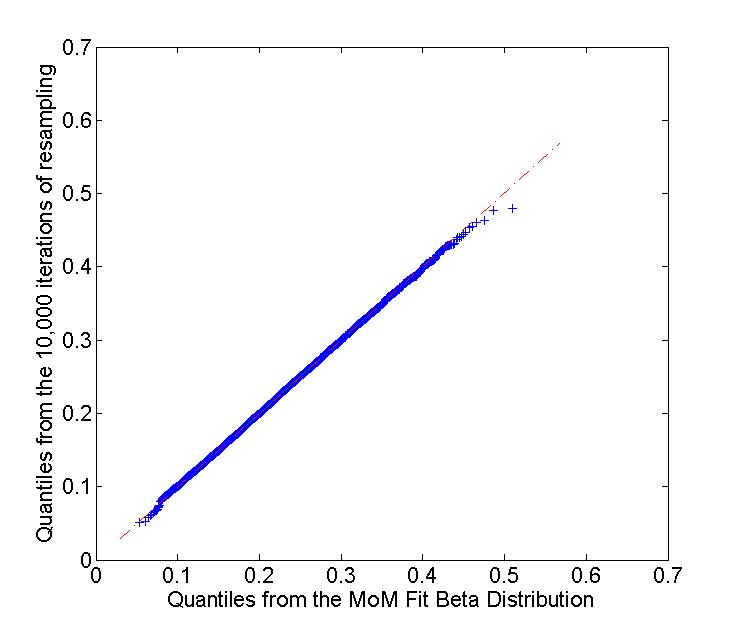

Supplement: Figure S1 — We computed the significance of the relationship between the lactic acidosis factors and the lactic acidosis signature by resampling the lactic acidosis signature weights and modeling the resulting scores with the factors. After 10,000 iterations, we fit the sampled r-squared values to a beta distribution. This figure shows a Q-Q plot of the distribution of resampled values versus the best fit beta distribution. Using this beta distribution, we find that the r-squared value from regressing the true signature scores on the factors is significant with p-value approximately 1e-13. (0.03 MB JPG) [file pone.0004523.s001.jpg]

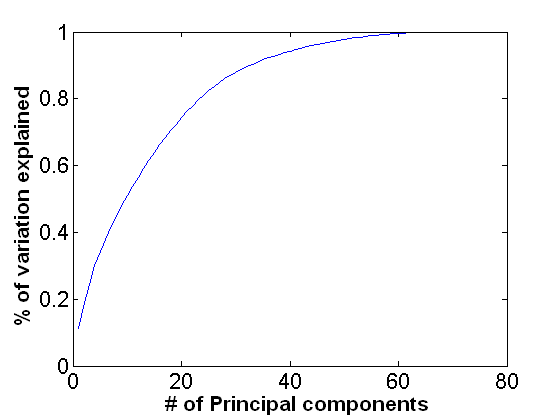

Supplement: Figure S2 — Percent of variation across all discovered factors as a function of the number of principal components used. (0.01 MB PNG) [file pone.0004523.s002.png]

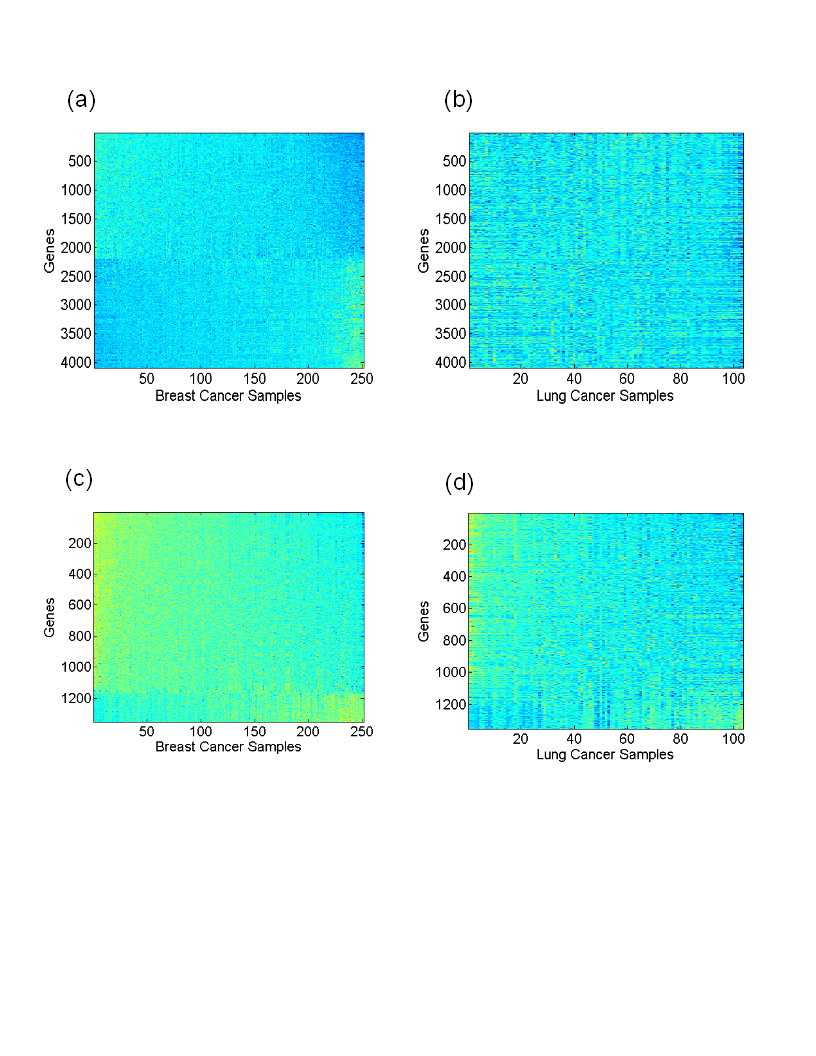

Supplement: Figure S3 — Figures (a) and (b) show the expression levels of the probes from the ER factor (discovered in breast tissue). (a) shows a conserved pattern of expression in the breast samples that is lost in the lung samples (b). (c) and (d) show the same figure, but for probes from the Tamoxifen susceptibility factor. For purposes of visualization, samples are sorted such that the first principal component is increasing. In figures (a) and (c) the rows are sorted according to increasing correlation with the first principal component. The ordering of the rows in figures (b) and (d) is forced to be the same as that in (a) and (c) respectively. (0.64 MB PNG) [file pone.0004523.s003.png]

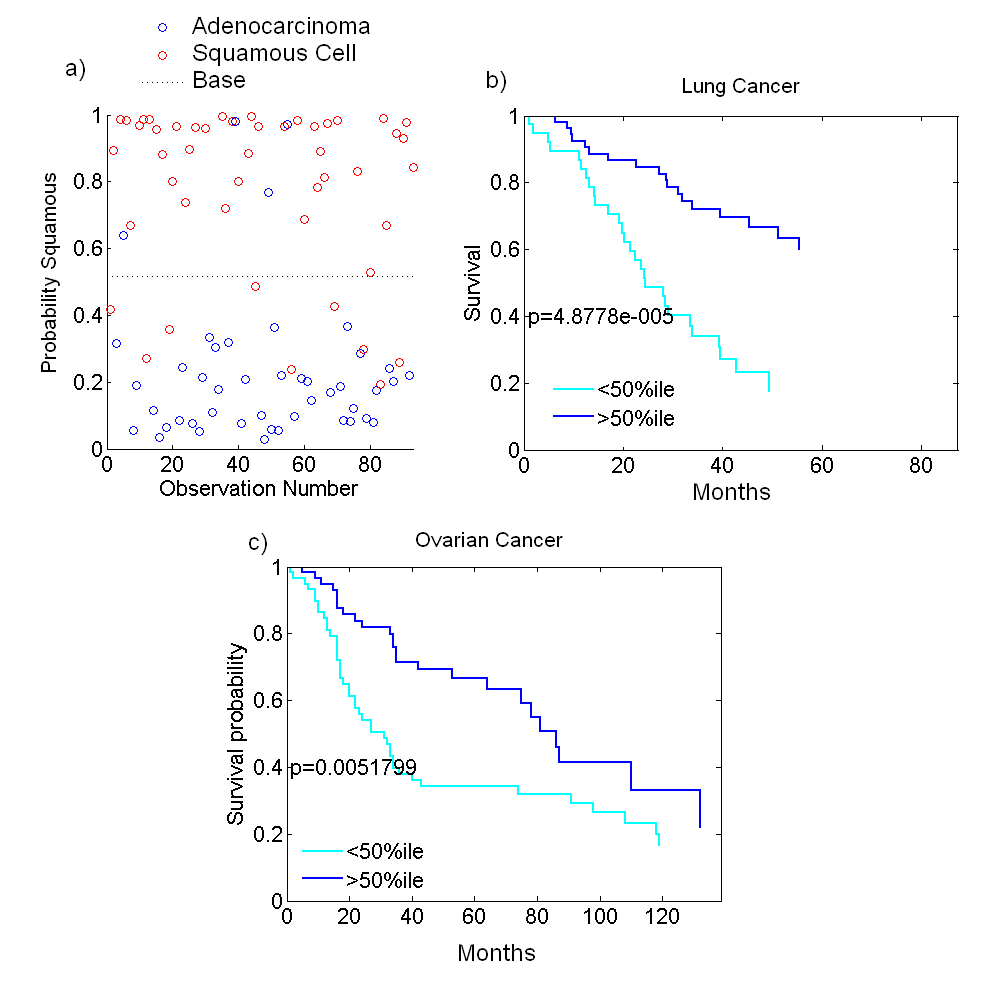

Supplement: Figure S4 — Lactic acidosis factors discovered in lung cancer can distinguish between adenocarcinoma and squamous cell carcinoma (a) as well as stratify patients according to rates of recurrence (b). Factors discovered in ovarian cancer have similar prognostic ability (c). (0.05 MB PNG) [file pone.0004523.s004.png]

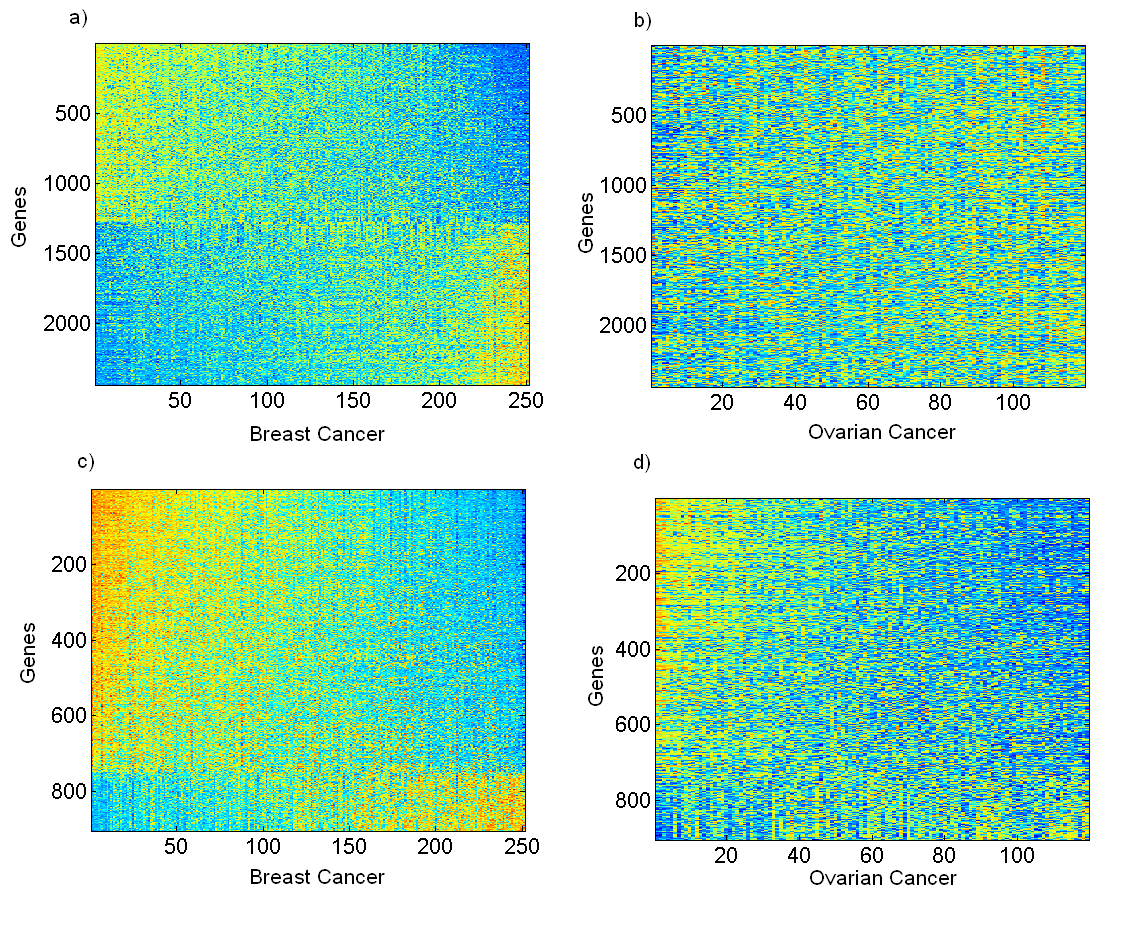

Supplement: Figure S5 — As in figure s3, probes show a consistent expression pattern in breast cancer that is missing in the ovarian cancer data set (a) and (b) while the tamoxifen susceptibility factor is conserved across the two data sets. (0.48 MB PNG) [file pone.0004523.s005.png]
