# Supplementary material for: Cross-Study Projections of Genomic Biomarkers: An Evaluation in Cancer Genomics
Source: PLoS One. 2009 Feb 19;4(2):e4523. doi: 10.1371/journal.pone.0004523 (PMC2638006; doi:10.1371/journal.pone.0004523)
Supplement: Supplemental Materials S1 — High dimensional sparse factor modeling: Applications in gene expression gneomics. Reference 17 is currently in press, so we have included it as supplementary material. (3.27 MB PDF) [file pone.0004523.s006.pdf]

High-Dimensional Sparse Factor Modelling: Applications in Gene Expression Genomics

Carlos Carvalho[*†], Jeffrey Chang[‡], Joe Lucas[*],

Joseph R Nevins[‡], Quanli Wang[‡] & Mike West[*]

[*]Institute of Statistics and Decision Sciences, Duke University, Durham NC 27708-0251

[†]Contact: *carlos@stat.duke.edu*

[‡]Institute for Genome Sciences and Policy, Duke University, Durham NC 27710

Abstract

In studies of molecular profiling and biological pathway analysis using DNA microarray gene expression data we are utilising a broad class of sparse latent factor and regression models for large-scale multivariate analysis and regression prediction. We present examples of these applications with discussion of key aspects of the modelling and computational methodology. Our case studies are drawn from breast cancer genomics, where we are concerned with the investigation and characterisation of heterogeneity of structure related to specific oncogenic pathways, as well as predictive/prognostic uses of aggregate patterns in gene expression profiles in clinical contexts. Based on the metaphor of statistically derived "factors" as representing biological "subpathway" structure, we explore the decomposition of fitted sparse factor models into pathway subcomponents, and how these components overlay multiple aspects of known biological structure in this network. We discuss the discovery and predictive uses of this approach, and the ability to use such models to generate enrichment of existing biological descriptions through identification of interactions between factors and subsequent experimental validation. We further illustrate the coupled use of predictive factor regression models with the high-dimensional sparse factor analysis of expression profiles.

Our methodology is based on sparsity modelling of multivariate regression, anova and latent factor models, and a general class of models that combines all components. Novel and effective sparsity priors address the inherent questions of dimension reduction and multiple comparisons, as well as scalability of the methodology. The models include practically relevant non-Gaussian/non-parametric components for modelling latent structure underlying often quite complex non-Gaussianity in multivariate expression patterns related to underlying biology. Model search and fitting are addressed through stochastic simulation and evolutionary stochastic search methods that are exemplified in oncogenic pathway studies. Supplementary supporting material provides more details of the applications as well as examples of the use of freely available software tools implementing the methodology.

Keywords: Biological pathways, Breast cancer genomics, Evolutionary stochastic search, Decomposing gene expression patterns, Dirichlet process factor model, Factor regression, Gene expression analysis, Gene expression profiling, Gene networks, Non-Gaussian multivariate analysis, Sparse factor models, Sparsity priors

1 Introduction

Gene expression assays of human cancer tissues provide data that reflect the heterogeneity characteristic of the cancer process. Figure 1 represents a few nodes in a central biological network of gene pathways, including the Rb/E2F pathway that is a major controlling component of mammalian cell growth, development and fate. As part of a much more complex network of interconnected signaling pathways (including the Ras, Myc and p53 response pathways) Rb/E2F is fundamental to the control of cell cycle, links the activity of cellular proliferation processes with the determination of cell fate, and is subject to many aspects of deregulation that relate to the development of human cancers. Many small changes due to spontaneous somatic mutations and the host of environmental influences overlaid on inherited characteristics induce subtle variation and deregulation in nodes in this network; natural variability in gene/protein functioning and communication results. This induces variations in cell proliferation control and many other "downstream" processes, some aspects of which can be reflected in large-scale gene expression assays from cell lines or tissue assays. The studies illustrated here use gene expression analysis on human breast cancer tissue samples in studies aiming to improving our understanding of aspects of this and related pathways; the overall goals are to better characterise the state and nature of a tumour based on expression profiles and to link to prognostic uses of expression profiles.

Our statistical framework utilises sparse latent factor models of the multivariate gene expression data, and allows extensions for regression and anova components based on explanatory variables as well as predictive regression components for measured responses. The approach builds on the initial class of sparse factor models and the analysis framework introduced in West (2003). In modelling dependencies among many variables – here the gene expression measurements across genes – we use linear latent factor models in which the factor loadings matrix is sparse, i.e., each factor is related to only a relatively small number of genes, representing a sparse, parsimonious structure underlying the associations among genes. With genes related to a set of interacting pathways, one key idea is that recovered factors overlay the known biological structure and that genes appearing linked to any specific "pathway characterising" factor may be known or otherwise putatively linked to function in that pathway. The modelling approach provides for biological information to be infused in the model in a number of ways, but also critically serves as an exploratory analysis approach to enrich the existing biological pathway representations. This analysis is en-

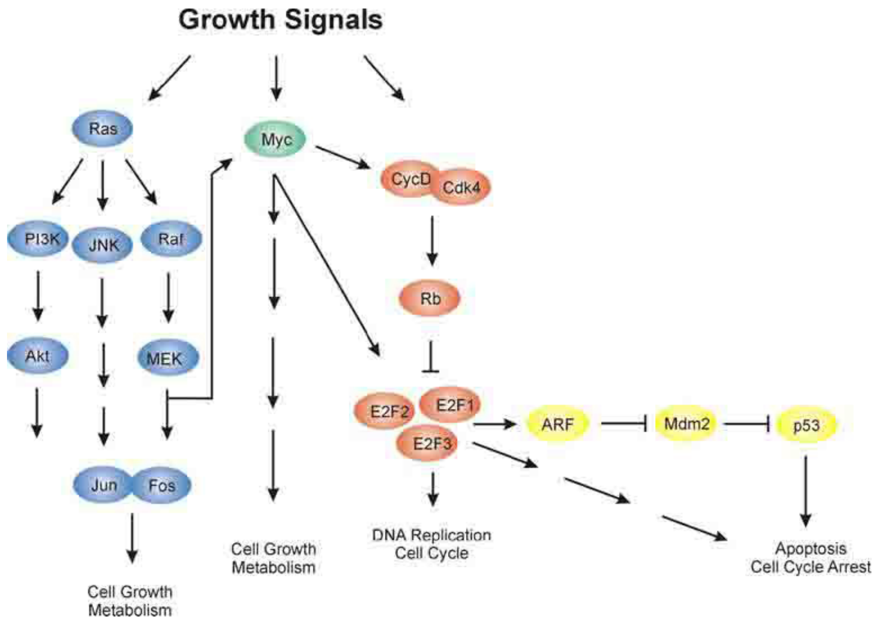

Figure 1: Schematic of key cell signalling pathways involved in cancer. The diagram illustrates some of the known activating and repressive actions of genes and proteins in the Rb/E2F pathway as well as connections to other key pathways. For example, the transcription factors Myc plays a role in activating CycD (Cyclin D) and the family of E2F transcription factors, while the retinoblastoma protein Rb represses (indicated by $\perp$) the transcription of the E2F family (Nevins, 1998).

abled through the use of a flexible class of sparsity-inducing priors that allow the introduction of arbitrary patterns of zeros in sets of factor loadings and regression parameters, so that data can inform on the sparsity structure and.

One other key methodological development is the use of non-parametric model components for the distributions of latent factors; this allows for flexible adaption to the often radically non-Gaussian structure in multiple aspects of the high-dimensional distributions of gene expression outcomes, reflecting aspects of experimental/technological noise as well as the more important non-Gaussianity that relates to biological heterogeneity.

The statistical methodology involves computation using evolutionary stochastic search and MCMC methods, and is described and illustrated in the examples. The evolutionary component uses the theory underlying MCMC methods for our sparse latent factor models to generate a variable selection method that is useful in enriching an existing model with new variables (here genes)

that appear to relate to the factor structure identified by an existing set of genes already modelled. In the pathway study context, model-based analysis of genes linked to a known biological pathway naturally recommends beginning with genes (variables) of known relevance and then gradually exploring beyond these initial variables to include others showing apparent association so as to“evolve” the model specification to higher-dimensions. This method meshes with MCMC analysis in the sparse factor models on a given set of genes. Our examples focused on the Rb/E2F signalling pathway and also hormonal pathways illustrate the methodology as an approach to exploring, evaluating and defining molecular phenotypes of sub-pathway characteristics – for both characterisation and prediction – in this important disease context. We conclude with comments about software for these analyses, as well as open issues and current research directions.

2 General Factor Regression Model Framework

The overall model framework combines latent factor modelling of a high-dimensional vector quantity **x** with regression for a set of response variables in a vector **z**, while allowing for additional regression and/or anova effects of other known covariates **h** on both **x** and **z**. In our gene expression case studies, **x** represents a column vector of gene expression measures on a set of genes in one sample, **z** a set of outcomes or characteristics, such as survival time following surgery or a hormonal protein assay measure, and **h** may represent clinical or treatment variables, or normalisation covariates relevant as assay correction factors for the gene expression data, for example.

2.1 Basic Factor Regression Model Structure

Observations are made on a $p-$dimensional random quantity **x** with the ith sample modelled as a regression on independent variables combined with a latent factor structure for patterns of covariation among the elements of $\mathbf{x}_i$ not explained by the regression. That is,

$$\mathbf{x}_i = \boldsymbol{\mu} + \mathbf{B}\mathbf{h}_i + \mathbf{A}\boldsymbol{\lambda}_i + \boldsymbol{\nu}_i, \qquad i = 1:n, \tag{1}$$

or, elementwise,

$$x_{g,i} = \mu_g + \boldsymbol{\beta}'_g h_i + \boldsymbol{\alpha}'_g \boldsymbol{\lambda}_i + \nu_{g,i} \;=\; \mu_g + \sum_{j=1}^{r} \beta_{g,j} h_{j,i} + \sum_{j=1}^{k} \alpha_{g,j} \lambda_{j,i} + \nu_{g,i} \tag{2}$$

for $g = 1 : p$ and $i = 1 : n$. These equations have the following components:

- $\boldsymbol{\mu} = (\mu_1, \dots, \mu_p)'$ is the $p-$vector of intercept terms.
- **B** is the $p \times r$ matrix of regression parameters $\beta_{g,j},\ (g = 1 : p, j = 1 : r)$, having rows $\boldsymbol{\beta}'_g$.
- **A** is the $p \times k$ matrix of factor loadings $\alpha_{g,j},\ (g = 1 : p, j = 1 : k)$, having rows $\boldsymbol{\alpha}'_g$.
- $\mathbf{h}_i = (h_{1,i}, \dots, h_{r,i})'$ is the $r-$vector of known covariates or design factors for sample i.
- $\boldsymbol{\lambda}_i = (\lambda_{1,i}, \dots, \lambda_{k,i})'$ is the latent factor $k-$vector for sample i.
- $\boldsymbol{\nu}_i = (\nu_{1,i}, \dots, \nu_{p,i})'$ is the idiosyncratic noise or error $p-$vector.

Variation in $x_{g,i}$ not predicted by the regression is defined by the underlying common factors through the factor term $\boldsymbol{\alpha}'_g\boldsymbol{\lambda}_i$, while ψ_g is the unexplained component of variance of $x_{g,i}$, representing natural variation, technical and measurement error that is idiosyncratic to that variable. We use the traditional zero upper-triangular parametrization of **A** to define identifiable models, the parametrization in which the first k variable have distinguished status (Aguilar and West, 2000; Lopes and West, 2003; West, 2003). Here $\alpha_{g,g} > 0$ for $g = 1, \dots, k$, and $\alpha_{g,j} = 0$ for factors $j = g+1, \dots, k$ and $g = 1, \dots, k-1$. The choice of these k lead variables is then a key modelling decision, and one of the questions addressed in our development of evolutionary model search in Section 6 below. We refer to the lead, ordered k variables as the *founders* of the factors.

The factors $\boldsymbol{\lambda}_i$ are assumed independently drawn from a latent factor distribution $F(\cdot)$. Traditionally, $F(\boldsymbol{\lambda}_i) = N(\boldsymbol{\lambda}_i|\mathbf{0}, \mathbf{I})$ where **0** and **I** are the zero vector and identity matrix respectively (used generically); the zero mean and unit variance matrix are identifying assumptions. One of our key methodological developments, discussed below, introduces non-parametric factor models based on a Dirichlet process extension of this traditional latent factor distribution.

The residual error terms are assumed normal, $\boldsymbol{\nu}_i \sim N(\boldsymbol{\nu}_i|\mathbf{0}, \boldsymbol{\Psi})$ where $\boldsymbol{\Psi} = \text{diag}(\psi_1, \dots, \psi_p)$. Practically useful extensions to heavier-tailed errors using T distributions in place of the normal here are easily encompassed within the simulation based Bayesian analysis we develop and use, though details are omitted here.

2.2 General Predictive Factor Regression Models

The above multivariate model for **x** combines with predictive model components relating **x** to a vector of response variables **z** in general predictive factor regression models that provide our framework for this paper. This simply views the multivariate modelling (of **x**) and regression

prediction (for $\mathbf{z}$ given $\mathbf{x}$) as derivative of an overall multivariate model for $(\mathbf{z}, \mathbf{x})$ jointly. This extends the initial factor regression model of West (2003) to incorporate the view that predictions of $\mathbf{z}$ from $\mathbf{x}$ may be partly influenced by the latent factors $\boldsymbol{\lambda}$ underlying $\mathbf{x}$ as well via additional aspects of $\mathbf{x}$. These potential "additional aspects" of $\mathbf{x}$ are represented in terms of additional latent factors referred to as *response factors*. That is, we simply extend the model to include additional latent factors arising in predicting each of the individual response variables, adding to the existing model.

To be specific, suppose $\mathbf{z}$ is $q-$vector with i^{th} observation $\mathbf{z}_i = (z_{1,i}, \ldots, z_{q,i})'$ and that, initially, each element is continuous and the variables are modelled jointly with $\mathbf{x}$. In a normal model context, this is natural by the immediate extension of the factor model (1) in which $\mathbf{x}_i$ is simply extended to a $(p+q) \times 1$ vector $(\mathbf{x}_i', \mathbf{z}_i')'$. For simplicity in notation, we simply redefine $\mathbf{x}_i$ as the extended vector of $(p+q)$ elements with $x_{p+g} = z_g$ for $g = 1 : q$. The general model is then of the precise form specified in equation (1) with this extended dimension; that is, elementwise,

$$x_{g,i} = \mu_g + \boldsymbol{\beta}_g' h_i + \boldsymbol{\alpha}_g' \boldsymbol{\lambda}_i + \nu_{g,i} \ = \ \mu_g + \sum_{j=1}^{r} \beta_{g,j} h_{j,i} + \sum_{j=1}^{k+q} \alpha_{g,j} \lambda_{j,i} + \nu_{g,i} \tag{3}$$

for $g = 1 : (p+q)$ and $i = 1 : n$, and with the additional following changes:

- $\boldsymbol{\mu} = (\mu_1, \ldots, \mu_{p+q})'$ is the extended vector of intercepts for both $\mathbf{x}_i$ and $\mathbf{z}_i$ vectors.
- $\mathbf{B}$ is the extended $(p+q) \times r$ matrix of regression parameters of all elements of $\mathbf{x}_i$ and $\mathbf{z}_i$ on the regressor variables in $\mathbf{h}_i$. Now $\mathbf{B}$ has elements $\beta_{g,j},\ (g = 1 : (p+q), j = 1 : r)$, with rows $\boldsymbol{\beta}_g'$.
- $\mathbf{A}$ is the extended (in both rows and columns) $(p+q) \times (k+q)$ matrix of factor loadings $\alpha_{g,j},\ (g = 1 : (p+q), j = 1 : (k+q))$, having rows $\boldsymbol{\alpha}_g'$.
- $\boldsymbol{\lambda}_i = (\lambda_{1,i}, \ldots, \lambda_{k+q,i})'$ is the extended $(k+q)-$vector of latent factors, where the additional q are introduced as *response factors*.
- $\boldsymbol{\nu}_i = (\nu_{1,i}, \ldots, \nu_{p+q,i})'$ is the extended idiosyncratic noise or error vector with the additional q elements now related to $\mathbf{z}_i$; the variance matrix is extended accordingly as $\boldsymbol{\Psi} = \text{diag}(\psi_1, \ldots, \psi_{p+q})$.

Beyond notation, the key extension is the introduction of additional potential latent factors, the final q in the revised $\boldsymbol{\lambda}_i$ vectors, each linked to a specific response variable in $\mathbf{z}_i$. The structure of the extended factor loadings matrix $\mathbf{A}$ reflects this: each of the q response variables serves define

an additional latent factor, i.e., serves as a a founder of a factor, while the first k of the $\mathbf{x}$ variables in the order specified serve, as originally, to define the k factors in the latent model component reflecting inherent structure in $\mathbf{x}$. Thus the structure of $\mathbf{A}$ is

$$\mathbf{A} = \begin{pmatrix} \mathbf{A}_x & \mathbf{A}_{x,z} \\ \mathbf{A}_{z,x} & \mathbf{A}_z \end{pmatrix}$$

where both $\mathbf{A}_x$ and $\mathbf{A}_z$ have the structure as described in the initial model of $\mathbf{x}$ alone, i.e., the traditional zero upper-triangular parametrization. That is, the *structural constraints* on $\mathbf{A} = \{\alpha_{g,j}\}$ have two components. First, as in the initial model for $\mathbf{x}$ alone, the $p \times k$ matrix $\mathbf{A}_x$ has $\alpha_{g,g} > 0$ for $g = 1, \ldots, k$, and $\alpha_{g,j} = 0$ for $j = g+1, \ldots, k$ and $g = 1, \ldots, k-1$. Second, the square response factor loadings matrix $\mathbf{A}_z$ is lower triangular with positive diagonal elements, i.e., $\alpha_{p+g,p+g} > 0$ for $g = 1, \ldots, q$, and $\alpha_{p+g,p+j} = 0$ for $j = g+1, \ldots, q$ and $g = 1, \ldots, q-1$.

Different scales of response variables can be corrected so that all variables lie on the same scale; this simplifies specification of prior distributions over the elements of the $\mathbf{A}$ and $\mathbf{B}$ matrices, in particular. Additional considerations relates to specification of values or priors for the variance terms in $\mathbf{\Psi}$, some of which arise in connection with non-Gaussian responses, now mentioned.

2.3 Non-Gaussian Response Variables

The extensions to allow for binary, categorical and censored data (such as survival time data) can be incorporated through the use of additional response-defining latent variables. Some key examples include:

- Binary responses modelled by probit regressions. For example, interpret $z_{i,1}$ as the unobserved, underlying latent variable such that an observable response $y_{1,i} = 1$ if, and only if, $z_{1,i} > 0$, and fix the variance $\phi_1 = 1$ accordingly. Modifications to logistic and other link functions can be incorporated using standard methods (Albert and Johnson, 1999).
- Categorical responses modelled via cascades of probit (or other) binary variables. For example, an observable response variable $y_{1,i}$ taking values 0,1 or 2 is easily (and usually adequately) modelled as defined by two underlying latent variables $z_{1,i}, z_{2,i}$ – now two elements of $\mathbf{z}_i$ in the factor regression – such that *(i)* $z_{i,1} \leq 0$ implies $y_{1,i} = 0$, *(ii)* $z_{i,1} > 0$ and $z_{i,2} \leq 0$ implies $y_{1,i} = 1$, while *(iii)* $z_{i,1} > 0$ and $z_{i,2} > 0$ implies $y_{1,i} = 1$. In fact, the hier-

archical/triangular structure of the factor model for $\mathbf{z}_i$ makes this construction for categorical data most natural.

- Right-censored survival data modelled as censored, transformed normal data. One useful model has outcome data that are logged values of survival times, in which case $z_{1,i}$ represents the mean of the normal on the log scale for case i. For observed times, $z_{1,i}$ is observed; for a case right-censored at time c_i, we learn only that $z_{i,1} \geq c_i$.

In each case, the uncertain elements of $\mathbf{z}_i$ – whether due to the inherent latent structure of binary and categorical variables or the censored data in survival analysis – are included in MCMC analyses with all model parameters and latent factors. This standard strategy also applies to cases of missing data when some elements of $\mathbf{z}_i$ are simply missing at random, and in predictive assessment and validation analysis when we hold-out the response values of some (randomly) selected samples to be predicted based on the model fitted to the remaining data.

2.4 Non-Gaussian, Non-Parametric Factor Modelling

A relaxation of the Gaussian assumption for the population distribution of the latent factors is of interest in expression studies, as in other application areas, in view of quite commonly encountered non-Gaussian features in data. An example that highlights this is discussed further in the first application below (in Section 5). Figure 5 displays scatter plots of estimated factor values from the analysis of a sample of expression profiles from breast tumours, and the two factors are labelled as representing key biological growth factor pathways – that related to the growth factor hormone estrogen measured by the estrogen receptor factor, and that related to the tyrosine kinase growth factor HER2/ERB-B2. Each of the ER and HER2 pathways play notable roles in the pathogenesis of breast cancer. For the illustration of non-Gaussianity here, simply note that the scatter plot reflects something like three overlapping groups of tumours that can be identified as distinct biological subtypes of breast cancer. In particular, higher levels of the HER2 factor in this plot are consistent with the known prevalence of an amplification of the HER2-ν gene, or over-expression of its protein product, in about 25-35% of breast cancers. Over-expression of this receptor in breast cancer is associated with increased disease recurrence and worse prognosis.

A first step towards non-parametric modelling of the latent factor distribution $F(\boldsymbol{\lambda}_i)$ is to utilise the widely used Dirichlet process framework (Escobar and West, 1995, 1998; West et al., 1994;

MacEachern and Müller, 1998). The direct relaxation of the standard normal model simply embeds the normal distribution as a prior expectation of a Dirichlet process (DP) over what is now regarded as an uncertain $k-$variate distribution function $F(\boldsymbol{\lambda}_i)$. In standard notation, $F \sim Dir(\alpha F_0)$, a DP prior with base measure αF_0 for some total mass, or precision parameter, $\alpha > 0$ and prior expectation $F_0(\boldsymbol{\lambda}) = N(\boldsymbol{\lambda}|0, I)$. Write $\boldsymbol{\lambda}_{1:n} = \{\boldsymbol{\lambda}_1, \dots, \boldsymbol{\lambda}_n\}$ and, for each $i = 1 : n$, denote by $\boldsymbol{\lambda}_{-i}$ the set of $n-1$ factor vectors $\boldsymbol{\lambda}_i$ removed. A key feature of the DP model is the set of implied complete conditionals for $\boldsymbol{\lambda}_i$ (marginalising over the uncertain F). These are given by

$$(\boldsymbol{\lambda}_i|\boldsymbol{\lambda}_{-i}) \sim a_{n-1}N(\boldsymbol{\lambda}_i|0, I) + (1 - a_{n-1}) \sum_{r=1, r\neq i}^{n} \delta_{\lambda_r}(\boldsymbol{\lambda}_i) \tag{4}$$

where $\delta_{\lambda}(\cdot)$ is the Dirac delta function, representing a distribution degenerate at $\boldsymbol{\lambda}$, and $a_{n-1} = \alpha/(\alpha + n - 1)$. This means that, conditional on $\boldsymbol{\lambda}_{-i}$, the factor vector $\boldsymbol{\lambda}_i$ comes from the prior normal distribution with probability a_{n-1}, otherwise it takes the same value as one of the existing $\boldsymbol{\lambda}_r$, those $n-1$ values having equal probability. Hence in any sample of n factor vectors there will be some number of distinct values less than or equal to n, and the samples will be configured across that number of "clusters" in factor space; of course the latency means that we will never know the configuration or number, and all inferences average over the implied posterior distributions. Full details and supporting theory can be found in the above references. For our purposes here, the key is the utility of the DP model as a flexible and robust non-parametric approach that will adapt to non-Gaussian structure evident in data. The concentration of factor values on common values does also add value from the point of view of expression data modelling; for example, it allows for the representation of both "inactive" and "upregulated" biological pathways across a number of samples, while also permitting variation in levels of activity of a pathway across other samples. Generally, the expectation will be of a larger number of distinct values in any hypothetical realisation of $\boldsymbol{\lambda}_{1:n}$, and this is consistent with larger values of the precision parameter α, a parameter to be included in posterior analysis using the approach of Escobar and West (1995).

3 Sparsity Modelling

In the gene expression contexts as in other areas of application of factor models, a basic perspective is that of sparsity in the factor loadings matrix. That is, any given gene may associate with one

or a few factors, but is unlikely to be related to (or implicated in) latent structure involving many factors. In complement, any one factor will link to a number of genes, but generally a relatively (to p) small number. The same reasoning applies to the new response factors introduced in the combined predictive factor regression models for a vector of responses **z** together with **x**. That is, in problems with large p, the factor loadings matrix **A** of the general model of equation (3) will be expected to have many zero elements, though the pattern of non-zero values is unknown and to be estimated. A priori, each (of the unconstrained) $\alpha_{g,j}$ may be zero or take some non-zero value, so that relevant priors should mix point masses at zero with distributions over non-zero values as in standard Bayesian "variable selection" analyses in regression and other areas (Clyde and George, 2004; George and McCulloch, 1993; Raftery et al., 1997). This was initiated in factor models in West (2003), and parallels the development of the concept in other models including large p regression applications (Rich et al., 2005; Dressman et al., 2006; Hans et al., 2007) and in related graphical models (Dobra et al., 2004; Jones et al., 2005). The standard mixture priors (sometimes referred to as "slab and spike" priors) have been used effectively in anova and related models for gene expression by several groups (Broet et al., 2002; Lee et al., 2003; Ishwaran and Rao, 2003, 2005; Do et al., 2005). Our extensions of sparsity prior modelling below represent generalisations of the standard methods for multivariate regression and anova as well as extensions of the original sparse factor regression model versions in West (2003).

Precisely the same ideas apply to the regression on independent variables component, i.e., the regression parameter matrix **B** of equation (3). That is, among the many variables there may be some significantly related to proposed explanatory variables in $\mathbf{h}_i$, whether these be dummy variables for design factors or other measured covariates. In designed intevention experiments differential expression resulting from a treatment effect will be evident for some genes but not all, so that some or many of the corresponding elements of **B** will be zero. Another specific example in gene expression studies involves the use of so-called control or housekeeping genes to generate multiple reference expression measures that are supposed to be consistent and unvarying across experimental conditions or observational samples. Observed variation in such controls can then be attributed to systematic or random variations experimental protocols and expression array assays – referred to as *assay artifacts* – and used as covariate information to provide potential regression-based corrections for the p genes of interest. Though expression measures of some genes may be

subject to distortion by assay artifacts and hence the regression on such control variates significant for such genes, many others will be robust and unaffected by artifacts, so that corresponding regression parameters will be zero. This is a nice example that highlights the relevance of sparsity prior modelling on regression coefficients as well in parallel to its natural relevance in latent factor modelling. For the balance of this section we focus discussion of concepts on the factor loadings matrix **A** but the methodology implemented applies the same ideas, and resulting sparsity prior distributional models, to **B** also.

At a general level, the strategy of *sparsity prior modelling* builds on a class of prior distributions under which each element $\alpha_{g,j}$ of **A** has a probability $\pi_{g,j}$ of taking a non-zero value. A variety of model structures may then be overlaid through hierarchical priors for these *sparsity probabilities* as well as the priors for values of the non-zero elements in **A**. Our main class of these "point-mass mixture priors" involves novel extension of the more standard variable selection priors (as used in the above references, for example) that address and overcome a number of key shortcomings of the standard approach in higher dimensional problems (Lucas et al., 2006a). In particular, we model the factor loadings as conditionally independent with

$$\alpha_{g,j} \sim (1 - \pi_{g,j})\delta_0(\alpha_{g,j}) + \pi_{g,j} N(\alpha_{g,j}|0, \tau_j) \tag{5}$$

independently over g, where $\delta_0(\cdot)$ is the Dirac delta function at zero. This states that variables have individual probabilities of association with any factor, $\pi_{g,j}$ for variable g and factor j, and that non-zero loadings on factor j are drawn from a normal prior with variance τ_j. A slight modification is required for the cases of diagonal elements since they are constrained to be positive to ensure identifiability; thus the normal component of equation (5) is adapted to $N(\alpha_{g,g}|0, \tau_j)I(\alpha_{g,j} > 0)$ for $g = 1, \ldots, k$ and $g = p+1, \ldots, p+q$, where $I(\cdot)$ is the indicator function.

The usual variable selection prior model adopts $\pi_{g,j} = \pi_j$, a common chance ("base rate") of non-zero loading on factor j for all variables, and estimates this base rate π_j under a prior that heavily favours very small values. The problem is that, with larger p, a very informative prior on π_j favouring very small values is required, and resulting posterior probabilities for $\alpha_{g,j} \neq 0$ that are quite spread out over the unit interval; while generally consistent with smaller values of π_j, this leads to a counter-intuitively high level of uncertainty concerning whether or not $\alpha_{g,j} = 0$ for a non-trivial fraction of the variables. This was clearly illustrated in West (2003) and has been demonstrated in other models with use of these standard priors (Lucas et al., 2006a).

An effective resolution of this problem is available within the more general model (5) by adding an appropriate hierarchical component for the loading probabilities $\pi_{g,j}$. Sparsity indicates that many of these probabilities will be small or zero, and a small number will be high. About the simplest reflection of this key view is to model these probabilities as drawn from a prior of the form

$$\pi_{g,j} \sim (1-\rho_j)\delta_0(\pi_{g,j}) + \rho_j Be(\pi_{g,j}|a_j m_j, a_j(1-m_j)) \tag{6}$$

where $Be(\cdot|am, a(1-m))$ is a beta distribution with mean m and precision parameter $a > 0$. Each ρ_j has a prior that quite heavily favours very small values, such as $Be(\rho_j|sr, s(1-r))$ where $s > 0$ is large (e.g., $s = p+q$) and r a very small prior probability of non-zero values, usually taken as $r_0/(p+q)$ for some small integer r_0 (e.g., $r_0 - 5 - 10$). The beta prior on non-zero values of $\pi_{g,j}$ is fairly diffuse while favouring relatively larger probabilities, such as defined by $a_j = 10$ and $m_j = 0.75$, for example. Note that, on integrating out the variable-specific probabilities $\pi_{g,j}$ from the prior for $\alpha_{g,j}$ in equation (5), we obtain a similar distribution but now with $\pi_{g,j}$ simply replaced by $E(\pi_{g,j}|\rho_j) = \rho_j m_j$; this is precisely the traditional variable selection prior discussed above, with the common base-rate of non-zero factor loadings set at $\rho_j m_j$. The insertion of the additional layer of uncertainty between the base-rate and the new $\pi_{g,j}$ now reflects, however, the view that many (as represented by a high value of ρ_j) of the loadings will be zero for sure, and permits the separation of significant factor loadings from the rest. The practical evidence of this is that, in many examples we have studied, the posterior expectations of the $\pi_{g,j}$ generally have a large fraction heavily concentrated at or near zero, a smaller number at very high values, and with only a few in regions of higher uncertainty within the unit interval. In contrast, the standard variable selection prior leads to posterior probabilities on $\alpha_{g,j} = 0$ that are overly diffused over the unit interval – more discussion and examples in regression variable selection in anova models appears in Lucas et al. (2006a). That is, the model now has the ability to ability to much more effectively detect non-zero loadings, and to induce very substantial shrinkage towards zero for many, many loadings – effectively resolving signal and the implicit multiple comparison problem through an appropriately structured hierarchical model.

4 Prior to Posterior Analysis via MCMC Computation

Assume sparsity priors specified independently for each of the columns of $\mathbf{A}$ and $\mathbf{B}$. Model completion then requires specification of priors for the variance components in $\mathbf{\Psi}$ and the τ_j of the sparsity priors. This will involve consideration of context and ranges of variation of noise/error components. The priors for $\psi_{p+1}, \ldots, \psi_{p+q}$ will be response variable specific, though some values may be fixed as exemplified in the binary and categorical variable discussion above. Inverse gamma priors are the conditionally conjugate and will be used in general for the ψ_g and τ_j parameters. For the former, substantial prior information exists from prior experience with DNA microarrays across multiple experiments and observational contexts, and should be utilised to at least define location of proper priors. Finally, the hyper-parameters of the sparsity priors on factor loadings are to be specified, and we have already discussed general considerations earlier.

MCMC analysis for posterior simulation is effectively standard and can be implemented in a Gibbs sampling format. The components of sets of conditional distributions to iteratively simulate are noted here, though full details are omitted since most component conditional distributions are standard and the manipulations and simulations involved very much routine in applied Bayesian work. This comment applies also to the conditional posteriors for the latent factor vectors arising as a result of the non-parametric Dirichlet process structure; in other model contexts, this is nowadays a routinely utilised model component and MCMC is well developed and understood. Some specifics of the MCMC components related to the sparsity priors are developed. Importantly, much of the computation at each iteration can be done as a parallel calculation by exploiting conditional independencies in certain complete conditionals of the posterior distribution.

Write $\mathbf{x}_{1:n}$ for the set of n observations on the $(p+q)-$dimensional outcomes, and $\boldsymbol{\lambda}_{1:n}$ for the corresponding set of n $(p+k)-$dimensional latent factor vectors. For any quantity Δ – any subset of the full set of parameters, latent factors and variables – denote by $p(\Delta|-)$ the complete conditional posterior of Δ given the data $\mathbf{x}^*_{1:n}$ and all other parameters and variables. Then, the sequence of conditional posteriors to sample is a follows:

- Sample the conditional posterior over latent factors, $p(\boldsymbol{\lambda}_{1:n}|-)$. Under the Dirichlet process structure, this generates a set of some $d_n \leq n$ distinct vectors and assigns each of the $\boldsymbol{\lambda}_i$ to one of these vectors (Escobar and West, 1995, 1998). The inherent stochastic clustering underlying this assignment is algorithmically defined using the standard configuration sam-

pling of Dirichlet process mixture models. We simply note that, conditional on the data and all other model parameters, the model (3) can be re-expressed as a linear regression of each "residual" vector $\mathbf{x}_i - \boldsymbol{\mu} - \mathbf{B}\mathbf{h}_i$ on $\mathbf{A}\boldsymbol{\lambda}_i$, with the matrix $\mathbf{A}$ and the variance matrix $\boldsymbol{\Psi}$ of the regression errors known at the current values at each MCMC iterate. This then falls under the general regression and hierarchical model framework of Dirichlet mixtures as in West et al. (1994) and MacEachern and Müller (1998); we then have access to the standard and efficient configuration sampling analysis for resampling the $\boldsymbol{\lambda}_{1:n}$ at each MCMC step, as described in these references. For convenience, additional brief details are given in the Appendix A here.

- For all $j = 1, \ldots, q$, the use of inverse gamma priors for the τ_j leads to conditionally independent inverse gamma complete conditionals $p(\tau_j|-)$. These are trivially simulated.
- For all g for which ψ_g is not specified, the use of inverse gamma priors implies that the complete conditionals $p(\psi_g^*|-)$ are similarly inverse gamma, and so easily simulated.
- The main novel MCMC component arises in simulation of conditional posteriors for the elements $\alpha_{g,j}$ of $\mathbf{A}$ and $\beta_{g,j}$ of $\mathbf{B}$, together with their sparsity-governing probabilities $\pi_{g,j}$. The structure for resampling entries in $\mathbf{B}$ is completely analogous to that for $\mathbf{A}$ so we discuss here only the latter. For given factor index j, this focuses on the complete conditional posterior for the full $(p+q)-$ j^{th} column of $\mathbf{A}$, namely $\mathbf{a}_j = (\alpha_{1,j}, \ldots, \alpha_{p+q,j})'$.

  An efficient strategy is to sample the bivariate conditional posterior distribution for each pair $\{\alpha_{g,j}, \pi_{g,j}\}$ via composition – sampling $p(\alpha_{g,j}|-)$ followed by $p(\pi_{g,j}|\alpha_{g,j}, -)$. The model is such that, for a fixed factor index j, these pairs of parameters (as g varies from $g = j, \ldots, p^*$) are conditionally independent so that this sampling may be performed in parallel with respect to variable index g.

  – The first step is to draw $\alpha_{g,j}$ from its conditional posterior marginalised over $\pi_{g,j}$. This is proportional to the conditional prior of equation (5) but, as earlier discussed, with $\pi_{g,j}$ substituted by its prior mean $\rho_j m_j$, and then multiplied by the relevant conditional likelihood function; here it easily follows that this likelihood component contributes a term proportional to a normal density for $\alpha_{g,j}$. This defines a posterior that is a point-mass at zero mixed with a normal for $\alpha_{g,j}$ in the case of unrestricted parameters. The computation is more complicated for the diagonal elements due to the constraint to positivity; simulation of this is still standard and accessible using either direct calculation

or accept/reject methods.

  – The second step is to sample the conditional posterior $p(\pi_{g,j}|\alpha_{g,j},-)$, as follows: *(i)* if $\alpha_{g,j} \neq 0$, then $\pi_{g,j} \sim Be(a_j m_j + 1, a_j(1-m_j)))$; *(ii)* if $\alpha_{g,j} = 0$, then set $\pi_{g,j} = 0$ with probability $1 - \tilde{\rho}_j$ where $\tilde{\rho}_j = \rho_j(1-m_j)/(1-\rho_j m_j)$, and otherwise draw $\pi_{g,j}$ from $Be(a_j m_j, a_j(1-m_j)+1)$.

- Finally, draw each ρ_j independently from $p(\rho_j|-) = Be(sr + \sigma_j, s(1-r) + p + q - j - \sigma_j)$ where $\sigma_j = \#\{\pi_{g,j} \neq 0 : g = j+1, \ldots, p+q\}$.

5 Breast Cancer Genomics #1: Hormonal Pathways

5.1 Goals, Context and Data

A first application in breast cancer genomics draws on a large and very heterogenous dataset to provide some initial illustrations of several aspects of the factor regression modelling framework as well as applied aspects and practicalities of expression analysis. The dataset combines summary RMA measures of expression from Affymetrix u95av2 microarray profiles on three sets of breast cancer samples: 138 tumour samples from the previously published CODEx study (Huang et al., 2002, 2003; Nevins et al., 2003; Pittman et al., 2004) from the Sun-Yat Sen Cancer Center in Taipei, 74 additional samples from the same center collected a year or two later than the original CODEx samples, and 83 samples on breast cancer patients collected during 2000-2004 at the Duke University Medical Center. The combined set of $n = 295$ samples were processed using the standard RMA code from Bioconductor (www.bioconductor.org) and screened to identify 5671 genes showing non-trivial variation across samples.

The initial examples of analysis outputs concern $p = 250$ genes. The process of selection of these genes is discussed in Section 6. We aim to explore aspects of the patterns of variation and covariation in genes related to the two key biological growth factor pathways: the estrogen receptor (ER) pathway and the HER2/ERB-B2 pathway that are central to the pathogenesis of breast cancer. Previous studies have explored gene expression patterns predictive of both ER and HER2, the former representing a very large and complex network of genes playing roles in cell growth and development as well as hormonal regulation. In addition to the gene expression data we include in the study the clinical assays of protein levels related to both ER and HER2, based on traditional im-

munohistochemical (IHC) staining for protein expression in sections of each tumour. One interest relates to how mRNA signatures of biological variation in these key pathways relate to the global and cruder designations of ER positive or negative based on the IHC assays. Discordance between expression and protein measures arises from many factors, not the least of which is the geographical variation in expression (of both genes and proteins) throughout a tumour. For our purposes here, collections of genes included in the analysis are known to be regulated by, or co-regulated with, ER or other key genes in the ER pathway, and so can be expected to show patterns covariation that will drive the identification of ER factor structure in the latent factor model. HER2/ERB-B2, though a key and dominant biological factor in breast cancer through its roles in signal transduction pathways leading to cell growth and differentiation, is a much lesser player than ER in terms of the numbers of genes it interacts with, directly or indirectly, and so factor structure in expression will be expected to relate to fewer genes.

This illustrative analysis fits the model to the $p = 250$ genes together with $q = 2$ binary response variables defined by the IHC assays of ER and HER2. For each of the response variables there are substantial numbers of missing or uncertain/indeterminate outcomes, so that the analysis imputes/predicts a good fraction of the response values. The 2 response are the indicator of ER positive versus negative based on the protein assay, referred to simply as ER (91 negative, 143 positive, 61 missing or uncertain), and the corresponding HER2 measure (60 negative, 86 positive, 149 missing or uncertain).

5.2 Exploring Variable-Factor Associations and Sparsity Patterns

Examination of aspects of the posterior for the factor loadings $\alpha_{g,j}$ forms a key part of the model exploration. The Monte Carlo estimates of the posterior loading probabilities $\hat{\pi}_{g,j} = Pr(\alpha_{g,j} \neq 0|\mathbf{x}_{1:n})$ are central to this. High values define significant gene-factor relationships. Figure 2 provides one first broad visual summary of key aspects of a model fitted to the $p = 250$ selected genes together with the $q = 2$ specified binary response variables. This model analysis has $k = 10$ latent factors, and this figure displays aspects of the posterior distribution for the first p rows of the factor loadings matrix $\mathbf{A}$ that correspond to the loadings $\alpha_{g,j}$ for all genes $g = 1 : 250$ across the $k + q = 12$ factors. Frame (a) provides insight into the "skeleton" of the fitted model, displaying the indicator of $\hat{\pi}_{g,j} > \theta$ where $\theta = 0.99$ for this figure; frame (b)

displays the posterior estimates of loadings for those gene-factor pairs that pass this threshold, i.e., $\hat{\alpha}_{g,j} = E(\alpha_{g,j}|\alpha_{g,j} \neq 0, \mathbf{x}_{1:n})I(\hat{\pi}_{g,j} > \theta)$. These figures give a useful general impression of the relative sparsity/density of factors as well as the cross-talk in terms of genes significantly linked to multiple factors, and highlight the nature of the model.

In this example the estimated latent factors labelled 1,2,4 and 5 are founded by known ER related genes and have a number of genes known to be linked to the ER pathways with significant loadings. Factor 1 is a primary ER factor and strongly associated with the protein IHC assay for ER status (see Figure 5); factors 2,4 and 5 contain highly loaded genes know to relate to the ER gene pathways but do not seem directly related to the IHC measure. Factor 3 is founded by the primary sequence probe on the Affymetrix array for HER2/ERB-B2. The Affymetrix array has three separate probe sets with DNA oligonucleotides representing different sections of this gene, that has historically been referred to as both ERB-B2 and HER2, and this factor picks up co-variation in the three along with a small number of other genes (12 at the threshold of $\hat{\pi}_{g,j} > 0.99$), defining what we can therefore label the HER2 factor. Table 1 lists a few of these as well as few of the "top genes" on some other selected factors; these genes simply score most highly in terms of (absolute values of the) estimated factor loadings among all genes exceeding the 0.99 threshold on loading probabilities. All genes listed are known to be regulated by, co-regulated with, interactive/synergistic with, or, from prior experimental studies, co-expressed with ER for factors 1 and 5, and with HER2 for factor 3. These genes, and many others in the key ER related factors this analysis identifies, have been earlier identified and discussed in, for example, our prior studies of ER expression variation (Spang et al., 2001; West et al., 2001; Huang et al., 2003) as well as by other authors. Factor 5 is loaded on a very small number of ER related genes, led by the transcription factor TFF3 that is known to be estrogen responsive or associated with ER status; the emergence of this additional ER related factor indicates potential connections into the TFF3 related signalling pathway. In the list for Factor 1 we include some of these that are lower loaded though still significant. These include some specific genes – implicated in this primary ER factor – that are noted in further discussions below. Some of these arise in Factor 8; this factor has very few significantly loaded genes, and the top three here are all sequences from the Cyclin D1 gene – the Affymetrix array has three separate probe sets with DNA oligonucleotides representing different sections of Cyclin D1, and this factor picks up co-variation in the three along with a small number

of other genes, defining what we label a Cyclin D1 factor in the same way that we identify several ER factors and a single HER2 factor based on gene membership and what is known about some of the top genes in each factor. We return to discuss the Cyclin D1 factor – its biological connections and how it highlights some of the discovery utility of this modelling approach – below.

5.3 Factor Variation, Decompositions and Interactions

Exploring plots of estimated latent factors across samples can provide useful insights into the nature of the contributions of the factors to patterns of variation in expression gene-by-gene and also relationships across genes. For example, Figures 3 and 4 provide some such plots that add to the discussion above of the ER factor structure in the data set and also the utility of this form of analysis in revealing interacting pathways. Factors are plotted only in cases of significant gene-factor association ($\hat{\pi}_{g,j} > 0.99$), so that these figures indicate highly significant attribution of expression fluctuations to identified factors. Focus initially on the two upper frames of Figure 3; these represent two versions of Cyclin D1. The corresponding estimates of gene-factor loadings $\hat{\alpha}_{g,j}$ are approximately as follows: for gene PRAD1, loadings of 0.53 on the ER factor 1 and 0.83 on the Cyclin D1 factor 8; for gene BCL-1, 0.54 on the ER factor and 0.81 on the Cyclin D1 factor. The agreement is clear: Cyclin D1 expression fluctuations are, up to residual noise and the components labelled c3 and c2 to be discussed below, described by these two factors in an approximate 5:8 ratio. This is a nice example not only of the agreement between factor model decompositions for what by design should be highly related expression profiles, but also consonant with known biology. Cyclin D1 is a regulatory component of the protein kinase Cdk4 and, together, they mediate the phosphorylation and inactivation of the Rb protein. Thus, its activity is required for cell cycle transitions and control of growth and proliferation. It is known (Sabbah et al., 1999) that ER directly binds to the CCND1 gene which encodes the Cyclin D1 protein; this can promote cell proliferation in target tissues by stimulating expression of Cyclin D1 and thus progression of the cell cycle. This has been observed in a number of ways including the inhibition of Cyclin D1 indirectly through the application of the estrogen targeting drug tamoxifen (Kilker et al., 2004). The relationship has feedback through the regulation of ER itself by Cyclin D1; for example, Cyclin D1 also acts to antagonize BRCA1 repression of ER (McMahon et al., 1999; Wang et al., 2005). There are further experimentally defined interactions between Cyclin D1 and ER – with

consequences for the resulting levels of activation of each of the two pathways – as reviewed in Fu et al. (2004). Hence the description of Cyclin D1 expression fluctuations via a non-ER related cell cycle component (factor 8) together plus a significant ER related component is consonant with known regulatory interactions between the cell cycle/Cylin D1 pathway and the ER pathway; the factor analysis reveals and quantifies these interactions.

In the lower frame of Figure 3 the third Cyclin D1 gene probeset, CCND1, shows substantial association with the ER and cell cycle Cyclin D1 factors, as expected; the estimated loadings are somewhat reduced relative to those of the other two probesets, at about 0.45 for the ER factor and 0.73 for the cell-cycle factor relative to the 0.5/0.8 levels of the other two probesets. CCND1 shows additional significant association with latent factor $j = 4$ with an estimated coefficient of 0.24. Though not apparently related to the ER IHC response (unlike factor 1), factor 4 is loaded on genes that include several ER related genes and other cyclins. The founder variable for factor 4 is the LIV-1 gene that also scores highly on the primary ER factor 1. LIV-1 is well-known to be regulated by estrogen and is coregulated with estrogen receptor in some breast cancers, though not apparently in some other cancers. Factor 4 may reflect more complexity of the interactions between the ER and early cell-cycle pathways. The CCND1 gene probeset shows significant association with this factor though the practical contribution of factor 4 to expression levels of CCND1 is relatively small compared to that of the others. Finally, CCND1 is also related to latent factor $j = 10$; we discuss this factor further in the following section.

This example of three Cyclin D1 probesets highlights differences in data measured in different ways on a single gene, and the need to consider questions of robustness and data quality in measurement of mRNA levels; the third frame indicates some concern about the measurements for CCND1 in the early samples, transferred to the residuals for this probeset. One strength of the model is the realistic attribution of substantial levels of variation in expression data to residual, unexplained terms. In many cases, purely experimental artifact and noise can be evident concordantly across multiple genes; the sparse factor and regression model can then be effectively used to protect estimation of biological effects from such contamination, as we now discuss and exemplify.

5.4 Exploration of Factors and Artifacts in Microarray Data

Gene expression data measurements are often quite sensitive to small changes in the experimental conditions and can show evidence of variation that is purely experimentally derived rather than representing biological variation of interest. We have previously introduced the use of sparse regression terms in which the covariates are summary measures of so-called housekeeping or control gene data (Lucas et al., 2006a,b). With Affymetrix arrays, as used in this example study, we use the principal components of sets of between 60-100 housekeeping gene probesets as readouts of such assay artifacts. These measures are designed to produce mRNA expression levels that show little or no biological or hybridization variation across samples, so that concordant patterns in these genes that define systematic variation through dominant principal components are potential artifact correction terms. Experience across multiple studies has demonstrated that, indeed, substantial assay artifacts can be identified this way, and that, typically, variation over samples in some of the dominant housekeeping correction factors can be reflected in multiple genes of interest. Contamination by assay artifact is usually sporadic, affecting multiple genes but by no means all, and hence the immediate relevance of the sparse regression components of the model.

In this analysis the first five components of the expression measures on the housekeeping genes were used in this way. In the upper two frames of Figure 3 we see that, in addition to the significant associations with the ER and Cyclin D1 factors, the sister PRAD1 and BCL-1 probesets shows significant association with, respectively, control factors 3 and 2 indicated by the labels c3 and c2. The levels of contribution of these systematic bias predictors are rather small, but they are nevertheless significant in that the corresponding estimated probabilities $\hat{\pi}_{g,j}$ for these genes g and their corresponding control covariates j are very high. Other genes are contaminated by assay artifact appearing in control factors 1-5 though the first three dominate; for some genes – unlike these two – the practical effect is much greater and the model does an effective job of artifact correction. Without correction, the potential for false discovery and misleading inferences can be substantial, as illustrated in sparse ANOVA model examples in Lucas et al. (2006a,b).

Experimental artifacts and induced variation across samples reflected in multiple genes can also be picked up by latent factors. The housekeeping gene data can and routinely does prove very useful in predicting artifacts, but additional systematic variation that can be linked back to batch effects, sets of samples processed in different labs or under slightly different conditions at

different times, for example, can often be quite substantial and impact in complex ways on many genes. Analysis that allows latent factors to be included in the model because collections of genes show evidence of common components of structure across samples has the ability to soak-up non-biological variation of this kind. This is a strength of the sparse factor modelling approach: they can confer robustness, protecting the estimation of biologically interesting structure.

One relevant example here concerns the probeset of the primary ER gene itself; see the first frame of Figure 4. The ER gene is naturally significantly loaded on the primary ER factor 1, but has a much lower estimated loading than other ER related genes, and shows significant association with the second putatively ER-related factor 2. The other two factors appearing with significant loadings for ER itself are factors 7 and 10, the latter also loaded for CCND1 as mentioned above. Review of the genes substantially linked to factor 10 have not yielded biological connections and we regard it as representing modest levels of experimentally induced variations in a number of genes. The same conclusion applies to factor 7, but much more forcefully and transparently. First, quite a few genes show somewhat increased volatility in expression fluctuations over the first 30-40 samples; the samples are ordered temporally and the early samples were processed in a more rapidly evolving laboratory context – as the new Affymetrix technology was refined – than later samples, consistent with the view that increased artifactual effects might be expected. Second, these breast samples are comprised of three distinct studies, and there are two apparent step-changes in the plot of the estimated factor 7 over samples that demark these three groups of samples; the very clear step-changes at or about $n = 138$, the end of the samples from the first study, and a second though less abrupt change at or about $n = 212$, the end of the second study. The housekeeping gene-based artifact correction covariates partly pick-up and correct for study effects in many genes, but apparently there is more systematic study effect evident in the data and this factor 7 is very clearly describing such structure that, by definition, must impact on a set of genes. With respect to this ER gene itself, this exemplifies how the factor model can protect estimation of biological structure – the ER factors and the attribution and quantification of their relationships with this gene – in the context of substantial contaminating noise.

5.5 Response Factors and Expression Signatures of Hormonal Status

Figure 5 scatters the samples on the estimated values of ER factor 1 and HER2 factor 3, with colour coding by the measured IHC assays for ER and HER2. In these figures we see the biologically interpretable groupings – naturally with much overlap – of breast tumours into ER$+$/HER2$-$, ER$-$/HER2$-$ and HER2$+$ as designated by the broad IHC-based protein assay for hormonal status. Gene expression signatures, as defined here solely by these two primary latent factors linked to these hormonal pathways through significantly loaded genes, are capable of refining the ER and HER2 scales and placing each tumour on the biologically relevant continuum.

The model of course includes the binary ER and HER2 responses and two response factors for them. The model has $p+2$ entries in $\mathbf{x}_i$, the final two being the linear predictors in probit regressions for ER and HER2, respectively. Figure 6 illustrates the overall signatures of ER and HER2 in terms of the probit transforms of the posterior means of the linear predictors. The posterior turns out to strongly favour only rather modest additional predictive value in the gene expression data beyond that captured by the $k = 10$ latent factors; that is, the posteriors for the response factor loadings elements $\alpha_{g,j}$ for $g = p + 1, p + 2$ and $j = 11, 12$ are almost all very concentrated at zero. There are a few genes that contribute significantly to the ER response prediction over and above the ER latent factors (8 genes at $\hat{\pi}_{g,j} > 0.99$), but none do so for HER2 prediction; this can be seen in the images in Figure 2. For ER, it is notable that a further key signally receptor gene is significant and most highly loaded on the ER response factor; this is the HER3 gene, known to play roles in the development of more highly proliferative cellular states in breast cancers (Holbro et al., 2003) as well as biochemically partnering with HER2 in promoting cellular transformation. The top two genes loaded on the ER response factor are the two probe sets for HER3 on the Affymetrix array. One of these is displayed in the right frame of Figure 4, where the significant association with the primary latent ER factor 1 along with the ER response factor (labelled y1) is clear. Note also that this probeset for HER3 also loads significantly on the artifactual factor 7, as does the ER gene, and the assay artifact covariate c2. Though not displayed, the second probeset for HER3 has a fitted decomposition that is almost precisely the same in terms of the split between contributions from f1 and y1, though is not apparently significantly linked to the artifactual factors. As with Cyclin D1, this is an example of different probesets for one gene – here HER3 – that can behave somewhat differently in terms of expression read-outs. The model analysis nevertheless identifies and extracts

the commonalities. The posterior estimates $\hat{\alpha}_{g,j}$ for the two HER3 probes on the ER$+/-$ response factor 1 are each approximately -0.51, those on the primary ER latent factor 1 are approximately 0.46 and 0.47. Thus, the sparse factor model analysis clean-up the artifacts to find and quantify the relevant associations with biologically interpretable and predictive factors.

5.6 Non-Gaussian Factor Structure Linked to Biology

The relevance of the non-Gaussian model for latent factor distributions is quite apparent from the plots in Figure 5. Other pairwise scatter plots suggest elliptical structure for some factor dimensions, though the full joint distribution is evidently highly-non-Gaussian. The biologically interpretable groupings are identified by the use of the non-parametric model that is designed to flexibly adapt to what can be quite marked non-Gaussian structure.

Non-Gaussianity in the factor model naturally feeds through from the observed non-Gaussian structure observed in expression of many genes individually and in subsets. This can be highlighted in prediction, one aspect of which is in connection with subjective exploration of aspects of model fit. The posterior distribution for the Dirichlet process model for latent factors is easily simulated, so that we can easily simulate from the posterior predictive distribution of a future latent factor vector $\boldsymbol{\lambda}_{n+1}$; this leads to easy simulation of the approximate prediction distributions for future outcomes $\mathbf{x}_{n+1}$ by fixing model parameters in the loadings and noise variance matrices at posterior estimates. (The more formal technical method is to fit the model with explicit inclusion of the sample $\mathbf{x}_{n+1}$ treated as a missing value that is imputed in the overall MCMC, though we have not yet implemented this). Suppose a specific gene g is, based on the posterior from the model fitted to $\mathbf{x}_{1:n}$, clearly not associated with the regression component; that is, the posterior for the regression parameters $\boldsymbol{\beta}_g$ is highly concentrated around $\mathbf{0}$. For such a gene, all the action is in the latent factor component, so that simulating the posterior predictive distribution for $\boldsymbol{\lambda}_{n+1}$ translates, via the addition of simulated noise terms $\nu_{g,n+1}$, directly to predictions for $x_{g,n+1}$.

This was done for this analysis, and the graphs in Figure 7 simply select two of the bivariate margins involving four genes for which the posterior shows high association with one or more factors and no association with regression or artifactual effects. These are the HER2/ERB-B2 gene already discussed and the ER-related FOXA1 in the first frame, and the two genes TFF3 and CA12 highly related to ER, in the second frame. The predictive simulation generates large samples of

the full joint distribution of all genes in the model and the samples on these two selected bivariate margins are simply contoured for presentation. The actual data on these genes is scattered over the contours, and the concordance is some reflection of model adequacy – at least in these dimensions. Sequencing through many such plots provides a useful global assessment of overall model structure and at least some guide to genes for which the model may be lacking. These kinds of plots also again highlight the relevance of the non-Gaussian factor model structure that feeds through to represent the observed non-Gaussianity of observed expression gene by gene.

6 Evolutionary Stochastic Model Search

The analysis discussed above uses a model defined by a process of evolutionary stochastic model search and refinement that has been developed to address variable (gene) selection, choice/limitation on the number of factors and the specification of the order of the first k founding variables in the model. This model search method is heavily inspired by the interest in evaluation of patterns of expression of genes linked to an pathway study – such as the exemplified ER pathway – and we have used it in a number of recent studies. The method is of course of general interest and utility in other application areas, though we describe it here in this pathway exploration context.

Directly specifying and fitting models with large numbers of variables p and factors k is a challenge statistically and computationally. In applied contexts such as biological pathway exploration, attempting to fit models to all the available variables (genes) would in any case be misguided scientifically. In the breast cancer study above, scientific goals include developing further insights into the genes and proteins that play roles in two key hormonal and cell proliferative processes linked to cancer. There are two main parts to this. The first is to enrich the understanding of gene expression patterns describing relationships among genes already known or hypothesised to participate; evaluating sparse factor models with the notion of factors representing aspects of dissected pathway structure on defined sets of genes in then useful. The second is to enrich the biological understanding by identifying additional factor structure and drawing in additional genes that link to the known biology; that is, using factor models to generate additional latent factors and expanded sets of genes linked to them, while maintaining a focus on the "neighbourhood" of the initial biological pathways of main interest. Fitting models to all the genes, or very many, with a discovery

intent – were it even possible – would be misguided due to the complexity of patterns of variation in high-dimensions that would dominate and obscure the structure at the restricted pathway level.

Rather, an appropriate view is to start with an initial set of biologically relevant genes and then expand the model by adding in new genes that appear to be linked to the factors identified in the initial model, and then refitting the model to adapt by expanding the number of factors if the new variables suggest additional structure. Repeating this process to iteratively refine the model underlies our evolutionary model search.

The technical key is to note that, given an initial set of p_0 variables and a model denoted by M_0 with k_0 latent factors, we can view the model as embedded in a larger model on all $p >> p_0$ variables and $k > k_0$ factors and in which the extended matrix of loadings probabilities has $\pi_{g,j} = 0$ for $g > p_0$ and $k > k_0$. Within this "full" overarching model, consider any of these variables $g > p_0$ and ask if it should be added to the current model with a single non-zero factor loading on, say, latent factor $j \in 1 : k_0$. Based on model parameters fixed at their posterior means based on the current model, we can then compute, approximately, the conditional posterior probability of inclusion, i.e., just $\tilde{\pi}_{g,j} = Pr(\alpha_{g,j} \neq 0|\mathbf{x}_{1:n}, M_0)$ where M_0 in conditioning simply stands for the current model and estimated parameters (note that we use $\tilde{\pi}_{g,j}$ compared to $\hat{\pi}_{g,j}$ to denote these inclusion probabilities for variables currently not included in the set to which the model is fitted). Variables g with high values of $\tilde{\pi}_{g,j}$ are candidates for inclusion – these are variables showing significant associations with one or other of the currently estimated factors, and so provide directions for model expansion around the currently identified latent structure. We can then rank and choose some of these variables – perhaps those for which $\tilde{\pi}_{g,j} > \theta$ for some threshold or, more parsimoniously, a specified small number of them – and refit the model.

Expanding the set of variables may identify other aspects of common association that suggest additional latent factors; enriching the sample space provides broader exploration of the complexity of associations around the initial model neighbourhood. This promotes exploring an expanded model M_1 on the new $p_1 > p_0$ variables and with $k_1 = k_0 + 1$ latent factors for which the first k_0 variables remain ordered as under M_0 – the factor founders in M_0 are those of the first k_0 factors in M_1. We can then refit M_1 and continue. This raises the question of the choice of the variable k_1 as founder of the new potential factor. We address this by fitting the model with some choice of this variable – perhaps just a random selection from the p_1 variables in M_1; from this model we

generate the posterior probabilities $\hat{\pi}_{g,j}$ and choose that variable with highest loading on the new factor $j = k_1$. Then model M_1 is refitted with this variable as founder of the new factor, assuming that these probabilities are appreciable for more than one or two variables.

Algorithmically, the evolutionary analysis proceeds as follows:

- Initialise a model M_0 and $i = 0$. For $i = 0, 1, \ldots,$ do the following:
- Compute approximate variable inclusion probabilities $\tilde{\pi}_{g,j}$ for variables g not in M_i and relative to factors $j = 1 : k_i$ in M_i. Rank and select at most r variables with highest inclusion probabilities subject to $\tilde{\pi}_{g,j} > \theta$ for some threshold. Stop if no additional variables are significant at this threshold.
- Set $i = i + 1$ and refit the expanded model M_i on the new p_i variables with $k_i = k_{i-1} + 1$ latent factors. First fit the model via MCMC with a randomly chosen founder of the new factor, and then choose that variable with highest estimated $\hat{\pi}_{g,k_i}$ as founder; refit the model and recompute all posterior summaries, including revised $\hat{\pi}_{g,j}$. Reject the factor model increase if fewer than some small prespecified number of variables have $\hat{\pi}_{g,j} > \theta$, then cutting back to k_{i-1} factors. Otherwise, accept the expanded model and continue to iterate the model evolutionary search at stage $i + 1$.
- Stop if the above process does not include additional variables or factors, or if the numbers exceed some prespecified targets on the number of variables including in the model and/or the number of factors.

This analysis has been developed and evaluated across a number of studies, and offers an effective way of iteratively refining a factor model based on a primary initial set of variables – the nucleating variables – of interest. Computational efficiencies can be realised by starting each new model MCMC analysis using information from the previously fitted model to define initial values. Control parameters include thresholds θ on inclusion probabilities for both variables and additional factors at each step, a threshold to define the minimum number of significant variables "required" to add a new latent factor, and overall targets to control the dimension of the final fitted model – specified maximum number of variables to include out of the overall (large) p, and (possibly) a specified maximum number of latent factors.

The analysis of Section 5 used this approach, starting with a $k_0 = 2$ factor model for $p_0 = 14$ initial genes chosen based on known function in the ER and HER2 pathways. The evolutionary

search immediately identified initial ER and HER2 factors at M_0, and then iteratively added in new variables known to be related to ER and HER as the model was revised. The final model, replete with hormonal and growth pathway genes, reflects the ability of the evolutionary search analysis to explore and refine the initial pathway description and, as the discussions of Cyclin D1 and HER3 illustrate, discover interconnections with other known interacting pathways.

It should be remarked that, at any "final" model M_I, say, we have available the computed values $\tilde{\pi}_{g,j}$ for all variables g not included in M_I. Thus, again, the model M_I can be viewed as embedded in a model for the full set of p variables, and these $\tilde{\pi}_{g,j}$ can be accessed to further explore *predicted* variable-factor associations between currently identified factors and all variables outside the model. In the breast cancer hormonal study, for example, the evolutionary process was controlled to terminate once the dimensions exceeded 10 latent factors and 250 variables, but the predictions indicate additional variables potentially significantly related to several of the ER factors that, were the process to be continued, could be drawn into an expanded description. In this sense, the images displayed in Figure 2 are only part of the full story; the full images on several thousand genes and just these 10 factors include additional variables with high values of the predicted loadings on these factors, but are otherwise far sparser since many of the remaining thousands of genes are simply not related to the biological pathways these factors characterise.

7 Breast Cancer Genomics #2: The p53 Pathway and Clinical Outcome

7.1 Goals, Context and Data

A second application in breast cancer genomics explores gene expression data from a study of primary breast tumours described in Miller et al. (2005). One original focus of this study was the patterns of tumour-derived gene expression potentially related to mutation of the p53 gene, and we explore this and broader questions of pathway characterisation and expression-based recurrence risk prediction using sparse factor regression models. The p53 transcription factor is a potent tumor suppressor that responds to DNA damage and oncogenic activity. The latter is seen in the connection of the p53 pathway to the primary Rb/E2F cell signalling pathway (Figure 1).

The consequences of p53 activation, either by oncogenic events or DNA damage, is an arrest of the cell cycle or an induction of cell death (apoptosis). Hence the use of models to characterise and discover factor structure in expression patterns that may relate to known, putative or novel connections between these pathways is certainly of current interest. Mutations in p53 occur in roughly 50% of human cancers. Multiple direct mutations lead to deregulation of key aspects of the p53 pathway and thus play roles in raising risk and aggressiveness of cancer due to the inability to properly program cell death, among other things. A number of current anticancer therapies target the p53 pathway as a result. The limited efficacy of such therapies is another motivation for studies that enrich our understanding of the biological interactions in the Rb/E2F/p53 network and that aid in characterising functional interactions of signalling mechanisms central to the control of cell proliferation and oncogenic processes.

This data set (Miller et al., 2005) comprises expression profiles on RNA extracted from $n = 251$ primary breast tumours. The expression profiles were created on Affymetrix u133a+ microarrays which, after RMA processing and screening to identify genes (probesets) showing non-trivial variation across samples, generates about $p = 30,000$ genes for the analysis here. Coupled with the expression data are clinical and genetic information on each patient. In each patient the p53 gene was sequenced for mutations at a number of loci, so generating one initial key binary variable: p53 mutant versus wildtype (no other mutational information was made available). Clinical and pathological information includes recurrence survival and the usual binary ER status from IHC assays. Miller et al. (2005) aimed to identify a gene expression signature associated with p53 mutational status; the concept under investigation was that such a signature would better represent perturbations in the p53 pathway and could be use as a refined clinical risk predictor in the same way that expression signatures of ER, HER2 and so forth will provide improvements over IHC assays. With this in mind, we have developed a detailed sparse factor regression analysis to thoroughly explore gene expression patterns linked to the broader p53 pathway and its neighbourhood connections to other pathways. Our final analysis seems to effectively dissect p53 activation into latent factors that represent core aspects of known underlying biology, identifies p53 related factors that are unrelated to mutations as well as others that are, suggests new pathway connections that tie into cell developmental activities in the Rb/E2F pathways, and also contributes through the factor regression component to rather more accurate recurrence risk prediction.

7.2 Factor Model Analysis and Latent Structure Linked to p53

The starting point for our analysis is a set of 25 genes known to participate in the p53 pathway (Sherr and McCormick, 2002). The model includes 3 response variables: the binary p53 mutational status, the binary ER status, and the continuous, right censored log of time to death. The MCMC analysis easily incorporates censoring of the continuous response, imputing the censored survival times from relevant conditional distributions at each iteration (see subsection 2.3). The evolutionary model search allows the model to evolve and sequentially include genes related to the factors in any current model – beginning with this known nucleating set of p53 related genes – as well as genes associated with the response factors in the current model. Hence the analysis can simultaneously explore sub-branches of the p53 pathway while identifying its connections to the outcomes of interest; that is, the regression variable selection process is part of the evolutionary analysis. Using thresholds of $\theta = 0.75$ for both variable and factor inclusion probabilities, and constraining such that a minimum of 3 genes are required to exceed this threshold on a factor to include that factor in an expanded model, the analysis terminated with a model on 1,010 genes distributed across $k = 12$ latent factors and $q = 3$ response factors.

Exploration of genes significantly and highly loaded on each factor provides some detailed annotation of common biological function, and potential interpretations of some of the factors in terms of biology driving the activities of pathway components. All factors have "top genes" that are known to be associated with cell cycle and oncogenic activity. Table 2 presents a summary of some of the top gene-factor pairings, with comments on their association with the two binary responses ER positive/negative and p53 mutant/wildtype. Relevant aspects of factor variation over samples and are displayed in Figures 8 and 9.

Of the genes in the p53 expression signature of Miller et al. (2005), our factor model identifies all but two and they all have significant loadings on the p53 factors 1, 3 and 4. The exploration of the p53 pathway guided by the mutational status combined with a set of canonical p53 genes also gives us the opportunity to identify other instances of the pathway that are not affected by mutations. This expands our understanding of alternative ways, other than mutation, in which p53 activity may be affected in cancer processes. To illustrate this we select three genes known to be key players in the p53 pathway: BAX, PERP and SFN. Miller et al. (2005) observe that the expression profiles of these genes do not relate to p53 mutations, raising the question that this

might represent cross talk between p53 and other oncogenic pathways. In our analysis, these genes are significantly loaded on factors 2 and 10; these two factors are not apparently directly associated with p53 mutation status, nor do they contribute significantly to p53 binary regression prediction in the analysis; but, they are composed primarily of genes that participate in cell developmental and apoptosis, hence do reflect substructure in the p53 pathway.

7.3 p53, ER and Recurrence Prediction

Table 3 summarises the estimated probabilities and coefficients of the most highly weighted latent and response factors in the linear predictors of the three response variables. Additional predictive value is generated by the included response factors that linked in a number of genes to elaborate on the predictions from the expression factors themselves, and Table 4 provides some information on a few of the top genes of the response factors. It is of interest to explore the genes most highly defining the factors that are implicated in prediction. Miller et al. (2005) note that p53 wildtype and mutant tumours can be distinguished by molecular differences heavily influenced by three major gene clusters comprising genes involved in immune response, proliferation and estrogen response, respectively (Figure 5 of supplemental material of Miller). All of the genes listed in these published clusters appear in our model in factors 1 and 3 (that we annotate as ER factors), factor 4 (that we annotate as a proliferation/p53 factor) and factor 6 (that we annotate as an immunological response factor). Each of these listed factors is directly associated with p53 status as evidence in Figures 8 and 9. A few examples of relevant genes in each of these factors are displayed in Figure 10 using the gene-factor decomposition format presented earlier.

The predictive uses of the model, and some informal predictive evaluations, are highlighted in Figures 11 and 12. The model analysis summarised was in fact based on fitting to a randomly selected 201 samples as training data, treating the remaining 50 as test or validation samples to be predicted. Figure 11 provides some indication of the within-sample discrimination for the two binary responses, p53 and ER, together with the out-of-sample predictive discrimination. Evidently, the combined factor regression model is capable of quite accurate prediction of both ER and p53 mutational status; these predictions are based on the integration over a few latent factors rather than a single direct signature, a point that the clinical genomics community has often, perhaps, undervalued in studies to develop genomic prognostics (Huang et al., 2003; Pittman et al., 2004; West

et al., 2006). The mutational outcome itself is certainly too complex, in terms of the apparent associations with multiple factor signatures in Figure 8, to be so accurately discriminated and predicted based on a single linear weighted signature of a set of genes. Figure 12 provides similar insight into the nature of the predictions for cancer recurrence, displayed in a format consistent with the use of expression signatures to indicate patient stratification into risk groups (Huang et al., 2003; Pittman et al., 2004). Here the test and validation samples, separately, are split by thresholds on the linear predictor of the survival regression on factors. The concordance between the resulting displays for test and training data is excellent and support the statistical validity of these model predictions; the potential practical relevance lies in the fact that these predictions are substantially more accurate than those based on stratification purely by p53 status as is currently commonly used.

The exploration of the p53 pathway guided by both mutational status and transcriptional activity of genes known to be associated in the biological process of interest is a key strategy in the generation of new biological hypothesis to be further examined. In this analysis, one of the factors with direct association with p53 (factor 7) and labeled as a cell development factor had a series of genes related to the RAS pathway through oncogenes FOS and JUN; see Figure 1 where the RAS/FOS-JUN pathways are linked to the Rb/E2F network. This discovery of significant gene expression factor structure linked to the complex p53 pathway suggests a connection between two very important branches of the major cell signalling network, raising questions to be explored and highlighting the potential discovery uses of this analysis.

8 Closing Comments

Sparsity of model structures and parametrisations is fundamental to scaling of scientific models to the higher-dimensional problems that are becoming common in many areas. Gene expression genomics, with the studies exemplified here becoming more and more common, is one currently active such arena. Models of both multivariate distributions in high-dimensions and regression for prediction when there are many candidate predictors yield practicable methodologies only if the effective dimension is explicitly or implicitly reduced. Sparsity – in terms of low-dimensional relationships underlying high-dimensional patterns of association of many variables and defined via parametric and conditional independence constraints – is key to this reduction. We have demon-

strated some of the potential utility of sparse factor models and factor regression models in these applications, and are utilising this approach in a number of related studies in cancer genomics as well as non-cancer areas.

The breast cancer genomics applications here illustrate a range of uses of the sparse factor modelling framework. Key elements of the model framework include: the use of new sparsity-inducing prior distributions over factor loadings and regression coefficients alike, with the ability to more adequately screen out insignificant variable-factor pairings and highlight – with quantitative probabilistic assessments – associations of interest; the isolation of idiosyncratic noise terms; the coupling of response prediction with factor analysis in an overall framework; the ability to handle missing or censored responses, and missing data in the multivariate outcomes **x** space itself; the integration of non-Gaussian, non-parametric factor components that are practically relevant in reflecting structure in common underlying patterns and their implications for non-Gaussian marginal data configurations in **x** space, among others. Key elements of the model fitting and analysis framework include: the implementation of efficient MCMC methods for analysis of a specified model; the use of evolutionary stochastic search methods for model extension based on an initial specified set of variables; and the investigation of model implications through evaluation and visualisation of variable decompositions, and others.

Beyond extensive development of applied studies in a number of genomics applications, there are a number of currently interesting statistical and computational developments. These include investigations of the connections between the evolutionary stochastic model search approach and related search methods, including projection pursuit methods and also the shotgun stochastic search approach for regression variable selection (Hans et al., 2007) that has seen success in graphical modelling (Dobra et al., 2004; Jones et al., 2005) and a range of prognostic applications in "large p" regression variable uncertainty problems in gene expression genomics (Rich et al., 2005; Dressman et al., 2006). Additional, topical investigations include: extensions of the Dirichlet process latent factor model; investigations of non-linear variants of the overall framework utilising kernel regression methods; and related studies of theoretical aspects of this approach to joint distributional modelling in prediction problems that link to the use of unlabelled data (Liang et al., 2007). Further development and refinement of the software used for all analyses here, and that is available to interested readers (see Appendix B below), are also in process.

Acknowledgments

We acknowledge support of the National Science Foundation (grants DMS-0102227 and DMS-0342172) and the National Institutes of Health (grants NHLBI P01-HL-73042-02 and NCI U54-CA-112952-01). Any opinions, findings and conclusions or recommendations expressed in this work are those of the authors and do not necessarily reflect the views of the NSF or NIH.

Appendix A: Elements of Configuration Sampling for Factors

Conditional on the data and all other model parameters, set $\mathbf{e}_i = \mathbf{x}_i - \boldsymbol{\mu} - \mathbf{B}\mathbf{h}_i$ so that the model (1) can be re-expressed as a linear regression $\mathbf{e}_i = \mathbf{A}\boldsymbol{\lambda}_i + \boldsymbol{\nu}_i$, where the matrix $\mathbf{A}$ and the variance matrix $\boldsymbol{\Psi}$ of the errors $\boldsymbol{\nu}_i$ are fixed at the current values at each MCMC iterate. With $\boldsymbol{\lambda}_i \sim F$ independently, and $F \sim Dir(\alpha_0 F_0)$, this is special case of the regression and hierarchical model framework of Dirichlet mixtures as in West et al. (1994) and MacEachern and Müller (1998). The standard and efficient configuration sampling analysis for resampling the $\boldsymbol{\lambda}_{1:n}$ uses the following steps at each of the MCMC iterates.

1. *Step 1: Resampling configuration indicators.* For each $i = 1, \dots, n$ in sequence, do the following:

   - Remove $\boldsymbol{\lambda}_i$ from the set of currently assigned factor vectors, leaving the set of $n-1$ vectors in $\boldsymbol{\lambda}_{-i}$. This current set of simulated factor vectors $\boldsymbol{\lambda}_{-i}$ is configured into some $s \le n-1$ groups with a common value within each group. Denote the s distinct factor vectors by $\boldsymbol{\theta}_{1:s} = \{\boldsymbol{\theta}_1, \dots, \boldsymbol{\theta}_s\}$ and the configuration indicators $c_r = j$ to indicate that $\boldsymbol{\lambda}_r = \boldsymbol{\theta}_j$ for $r = 1, \dots, i-1, i+1, \dots, n$. Write n_j for the number of occurrences of $\boldsymbol{\theta}_j$ in $\boldsymbol{\lambda}_{-i}$, i.e., $n_j = \sum_{r=1, r\neq i}^{n} \delta_j(c_r)$.
   - The complete conditional posterior for $\boldsymbol{\lambda}_i$ is the mixture

   $$(\boldsymbol{\lambda}_i|-) \sim q_{i,0} N(\boldsymbol{\lambda}_i|\mathbf{m}_i, \mathbf{M}) + \sum_{j=1}^{s} q_{i,j}\delta_{\theta_j}(\boldsymbol{\lambda}_i),$$

   so that $\boldsymbol{\lambda}_i$ equals $\boldsymbol{\theta}_j$ with probability $q_{i,j}$, otherwise it is sampled anew from $N(\cdot|\mathbf{m}_i, \mathbf{M})$ with probability $q_{i,0}$. These moments and probabilities are as follows:

     – $\mathbf{m}_i = \mathbf{M}\mathbf{A}'\boldsymbol{\Psi}^{-1}\mathbf{e}_i$ and $\mathbf{M}^{-1} = \mathbf{I} + \mathbf{A}'\boldsymbol{\Psi}^{-1}\mathbf{A}$

- $q_{i,0} \propto \alpha N(\mathbf{e}_i|\mathbf{0}, \mathbf{A}\mathbf{A}' + \mathbf{\Psi})$ and, for $j = 1:s$, $q_{i,j} \propto n_j N(\mathbf{e}_i|\mathbf{A}\boldsymbol{\theta}_j, \mathbf{\Psi})$, where the notation N here denotes the evaluated multivariate normal densities.

Draw a new configuration indicator c_i from $0:s$ using the probabilities $q_{i,0:s}$. If $c_i = 0$, sample a new value $\boldsymbol{\lambda}_i \sim N(\boldsymbol{\lambda}_i|\mathbf{m}_i, \mathbf{M})$.

2. *Step 2: Resample unique factor vectors.* Following Step 1, the full set of resampled configuration indicators defines a set of (some final number) s conditionally independent linear regressions: for each group $j = 1:s$, the "data" in group j is the set of n_j observations $\mathbf{e}_i \sim N(\mathbf{e}_i|\mathbf{A}\boldsymbol{\theta}_j, \mathbf{\Psi})$ such that $c_i = j$. Resample the unique factor vector $\boldsymbol{\theta}_j$ of each group $j = 1:s$ from the implied conditional posterior $N(\boldsymbol{\theta}_j|\mathbf{t}_j, T_j)$ where $\mathbf{t}_j = \mathbf{T}_j\mathbf{A}'\mathbf{\Psi}^{-1}\sum_{i:c_i=j}\mathbf{e}_i$ and $\mathbf{T}_j^{-1} = \mathbf{I} + n_j\mathbf{A}'\mathbf{\Psi}^{-1}\mathbf{A}$.

Appendix B: Software

Efficient software implementing the MCMC and evolutionary stochastic search for the full class of sparse Bayesian factor and regression models is available to interested readers. The BFRM code implements the analysis in the framework of sparse latent factor models coupled with sparse regression and ANOVA for multivariate data, relevant in many exploratory and predictive problems with high-dimensional multivariate observations as well as the kinds of biological pathway studies of the applications here. The software additionally includes model components that allow for the missing and censored data, binary, categorical and continuous responses, hold-out analyses for predictive validation, and customisation to gene expression studies to include automatic handling of data issues (the generalized normalisation and assay artifact correction examples here as cases in point) that arise in all expression studies that combine data on microarrays across experimental conditions or laboratories. Interested readers can download the BFRM executable and review instructions and examples at the web site *http://icbp.genome.duke.edu/bfrm.html*. Examples include studies of complex networks of intersecting biological pathways in cancer genomics as in the studies reported here.

References

Aguilar, O. and West, M. "Bayesian dynamic factor models and portfolio allocation." *Journal of Business and Economic Statistics*, 18:338–357 (2000).

Albert, J. and Johnson, V. *Ordinal Data Models*. New York: Springer-Verlag (1999).

Broet, P., Richardson, S., and Radvanyi, F. "Bayesian hierarchical model for identifying changes in gene expression from microarray experiments." *Journal of Computational Biology*, 9:671–683 (2002).

Clyde, M. and George, E. "Model uncertainty." *Statistical Science*, 19:81–94 (2004).

Do, K., Mueller, P., and Tang, F. "A Bayesian mixture model for differential gene expression." *Journal of the Royal Statistical Society, Ser. C (Applied Statistics)*, 54:627–644 (2005).

Dobra, A., Jones, B., Hans, C., Nevins, J. R., and West, M. "Sparse graphical models for exploring gene expression data." *Journal of Multivariate Analysis*, 90:196–212 (2004).

Dressman, H. K., Hans, C., Bild, A., Olsen, J., Rosen, E., Marcom, P. K., Liotcheva, V., Jones, E., Vujaskovic, Z., Marks, J. R., Dewhirst, M. W., West, M., Nevins, J. R., and Blackwell, K. "Gene expression profiles of multiple breast cancer phenotypes and response to neoadjuvant therapy." *Clinical Cancer Research*, 12:819–216 (2006).

Escobar, M. and West, M. "Bayesian density estimation and inference using mixtures." *Journal of the American Statistical Association*, 90:577–588 (1995).

Escobar, M. D. and West, M. "Computing nonparametric hierarchical models." In Mller, P., Dey, D., and Sinha, D. (eds.), *Practical Non and Semiparametric Bayesian Statistics*, 1–16. New York: Springer-Verlag (1998). P. Mller et al. Springer-Verlag.

Fu, M., Wang, C., Li, Z., Sakamaki, T., and Pestell, R. "Minireview: Cyclin D1: Normal and abnormal functions." *Endocrinology*, 145:5439–5447 (2004).

George, E. and McCulloch, R. "Variable selection via Gibbs sampling." *Journal of the American Statistical Association*, 88:881–889 (1993).

Hans, C., Dobra, A., and West, M. "Shotgun stochastic search in regression with many predictors." *Journal of the American Statistical Association*, to appear (2007).

Holbro, T., Beerli, R., Maurer, F., Koziczak, M., Barbas, C. r., and Hynes, N. "The ErbB2/ErbB3 heterodimer functions as an oncogenic unit: ErbB2 requires ErbB3 to drive breast tumor cell proliferation." *Proceedings of the National Academy of Sciences*, 100:8933–8 (2003).

Huang, E., Chen, S., Dressman, H. K., Pittman, J., Tsou, M. H., Horng, C. F., Bild, A., Iversen, E. S., Liao, M., Chen, C. M., West, M., Nevins, J. R., and Huang, A. T. "Gene expression predictors of breast cancer outcomes." *The Lancet*, 361:1590–1596 (2003).

Huang, E., West, M., and Nevins, J. R. "Gene expression profiles and predicting clinical characteristics of breast cancer." *Hormone Research*, 58:55–73 (2002).

Ishwaran, H. and Rao, J. "Detecting differentially expressed genes in microarrays using Bayesian model selection." *Journal of the American Statistical Association*, 98:438–455 (2003).

—. "Spike and slab gene selection for multigroup microarray data." *Journal of the American Statistical Association*, 100:764–780 (2005).

Jones, B., Dobra, A., Carvalho, C., Hans, C., Carter, C., and West, M. "Experiments in stochastic computation for high-dimensional graphical models." *Statistical Science*, 20:388–400 (2005).

Kilker, R., Hartl, M., Rutherford, T., and Planas-Silva, M. "Cyclin D1 expression is dependent on estrogen receptor function in tamoxifen-resistant breast cancer cells." *Journal of Steroid Biochemistry and Molecular Biology*, 92:63–71 (2004).

Lee, K., Sha, N., Dougherty, E., Vannucci, M., and Mallick, B. "Gene selection: a Bayesian variable selection approach." *Bioinformatics*, 19:90–97 (2003).

Liang, F., Mukherjee, S., and West, M. "Understanding the use of unlabelled data in predictive modelling." *Statistical Science*, to appear (2007).

Lopes, H. and West, M. "Bayesian model assessment in factor analysis." *Statistica Sinica*, 14:41–67 (2003).

Lucas, J., Carvalho, C., Wang, Q., Bild, A., Nevins, J. R., and West, M. "Sparse statistical modelling in gene expression genomics." In Müller, P., Do, K., and Vannucci, M. (eds.), *Bayesian Inference for Gene Expression and Proteomics*, 155–176. Cambridge University Press (2006a).

Lucas, J., Wang, Q., Bild, A., Nevins, J. R., and West, M. "Sparse Bayesian analysis and data synthesis in gene expression experiments." Technical report, ISDS, Duke University (2006b).

MacEachern, S. N. and Müller, P. "Estimating Mixture of Dirichlet Process Models." *Journal of Computational and Graphical Statistics*, 7(2):223–238 (1998).

McMahon, C., Suthiphongchai, T., DiRenzo, J., and Ewen, M. "P/CAF associates with Cyclin D1 and potentiates its activation of the estrogen receptor." *Proceedings of the National Academy of Sciences*, 53825387 (1999).

Miller, L., Smeds, J., George, J., Vega, V., Vergara, L., Ploner, A., Pawitan, Y., Hall, P., Klaar, S., Liu, E., and Bergh, J. "An Expression Signature for p53 Status in Human Breast Cancer Predicts Mutation Status, Transcriptional Effects, and Patient Survival." *Proceedings of the National Academy of Sciences*, 102:13550–13555 (2005).

Nevins, J. "Toward an understanding of the functional complexity of the E2F and Retinoblastoma families." *Cell Growth and Differentiation*, 9:585593 (1998).

Nevins, J. R., Huang, E. S., Dressman, H., Pittman, J., Huang, A. T., and West, M. "Towards integrated clinico-genomic models for personalized medicine: Combining gene expression signatures and clinical factors in breast cancer outcomes prediction." *Human Molecular Genetics*, 12:153–157 (2003).

Pittman, J., Huang, E., Dressman, H., Horng, C. F., Cheng, S. H., Tsou, M. H., Chen, C. M., Bild, A., Iversen, E. S., Huang, A. T., Nevins, J. R., and West, M. "Integrated modeling of clinical and gene expression information for personalized prediction of disease outcomes." *Proceedings of the National Academy of Sciences*, 101:8431–8436 (2004).

Raftery, A., Madigan, D., and Hoeting, J. "Bayesian model averaging for linear regression models." *Journal of the American Statistical Association*, 92:1197–1208 (1997).

Rich, J., Jones, B., Hans, C., Iversen, E. S., McClendon, R., Rasheed, A., Bigner, D., Dobra, A., Dressman, H. K., Nevins, J. R., and West, M. “Gene expression profiling and genetic markers in glioblastoma survival.” *Cancer Research*, 65:4051–4058 (2005).

Sabbah, M., Courilleau, D., Mester, J., and Redeuilh, G. “Estrogen induction of the Cyclin D1 promoter: Involvement of a camp response-like element.” *Proceedings of the National Academy of Sciences*, 96:11217–22 (1999).

Sherr, C. and McCormick, F. “The Rb and p53 Pathway in Cancer.” *Cancer Cell*, 2:103–112 (2002).

Spang, R., Zuzan, H., West, M., Nevins, J. R., Blanchette, C., and Marks, J. R. “Prediction and uncertainty in the analysis of gene expression profiles.” *In Silico Biology*, 2:0033 (2001).

Wang, C., Fan, S., Li, Z., Fu, M., Rao, M., Ma, Y., Lisanti, M., Albanese, C., Katzenellenbogen, B., Kushner, P., Weber, B., Rosen, E., and Pestell, R. “Cyclin D1 antagonizes BRCA1 repression of estrogen receptor alpha activity.” *Cancer Research*, 65:65576567 (2005).

West, M. “Bayesian factor regression models in the ”large p, small n” paradigm.” In Bernardo, J., Bayarri, M., Berger, J., Dawid, A., Heckerman, D., Smith, A., and West, M. (eds.), *Bayesian Statistics 7*, 723–732. Oxford University Press (2003). J.M. Bernardo et al. Oxford.

West, M., Blanchette, C., Dressman, H., Huang, E., Ishida, S., Spang, R., Zuzan, H., Marks, J. R., and Nevins, J. R. “Predicting the clinical status of human breast cancer utilizing gene expression profiles.” *Proceedings of the National Academy of Sciences*, 98:11462–11467 (2001).

West, M., Huang, A. T., G.Ginsberg, and Nevins, J. R. “Embracing the complexity of genomic data for personalized medicine.” *Genome Research*, 16:559–566 (2006).

West, M., Müller, P., and Escobar, M. D. “Hierarchical priors and mixture models, with application in regression and density estimation.” In Smith, A. and Freeman, P. (eds.), *Aspects of Uncertainty: A Tribute to D.V. Lindley*, 363–386. London: Wiley (1994).

(a) (b)

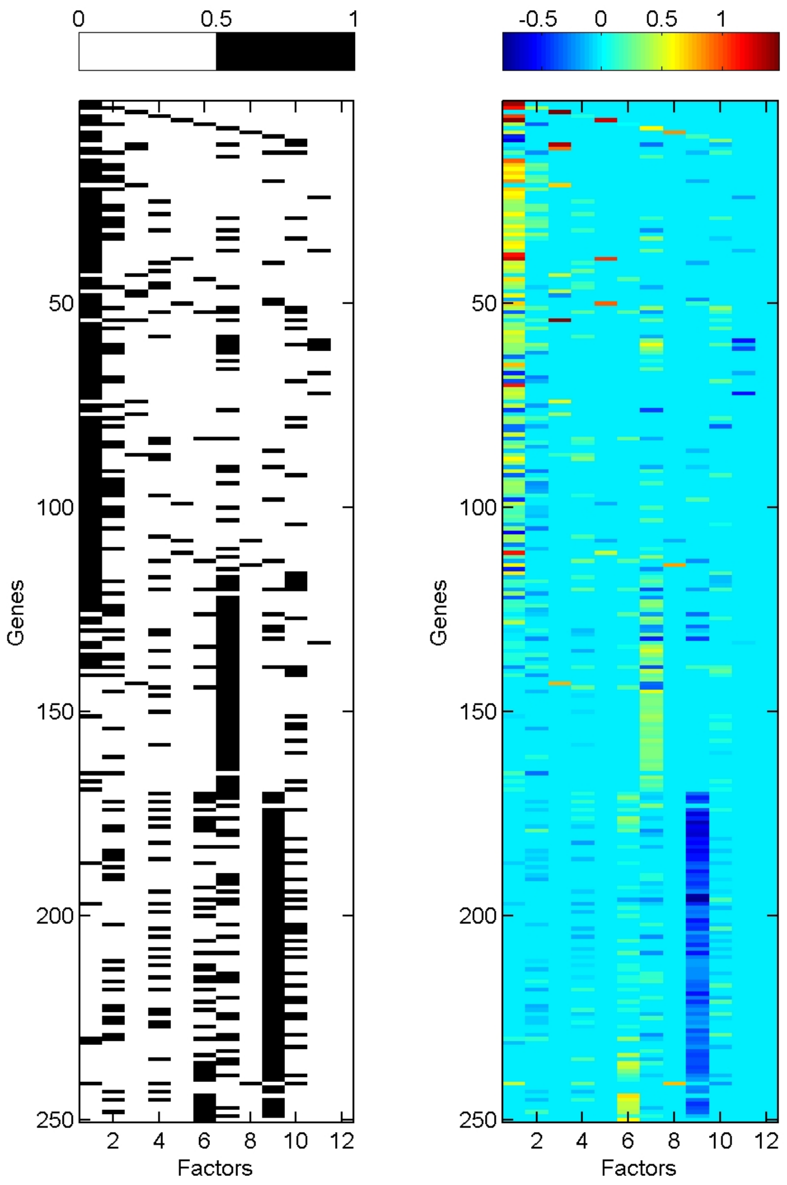

Figure 2: Breast cancer hormonal pathways. Skeleton of the fitted model for the 250 selected genes and 12 factors. Left frame: (binary) heatmap of thresholded approximate posterior loading probabilities $I(\hat{\pi}_{g,j} > 0.99)$. Right frame: heatmap of approximate posterior means of significant gene-factor loadings $\hat{\alpha}_{g,j}$.

	$\hat{\alpha}_{g,j}$	Gene	Gene symbol
Factor 1:			
	1.5	Intestinal trefoil factor	TFF3
	1.4	Carbonic anhydrase precursor	CA12
	1.3	Clone AA314825:EST186646	-
	1.1	Secreted cement gland protein XAG-2 homologue	AGR2
	1.1	Hepatocyte nuclear factor-3 alpha (HNF-3α)	FOXA1
	1.1	Trans-acting T-cell specific transcription factor	GATA-3
	1.1	Clone AL050025:DKFZp564D066	-
	1.0	Breast cancer, estrogen regulated LIV-1 protein	LIV-1
		...	
	0.71	Myeloblastosis viral oncogene homolog	C-MYB
	0.47	Human epidermal growth factor receptor	HER3
	0.46	Human epidermal growth factor receptor	HER3
	0.44	BCL-2	BCL-2
	0.42	Androgen receptor	AR
		...	
	0.54	PRAD1 (cyclin D)	CCND1
	0.53	BCL-1 (cyclin D)	CCND1
	0.45	CYCD1 (cyclin D)	CCND1
Factor 3:			
	1.5	c-Erb-B2	ERB-B2
	1.4	Human tyrosine kinase-type receptor (HER2)	HER2b
	1.4	Human tyrosine kinase-type receptor (HER2)	HER2
	0.93	Growth factor receptor-bound protein 7	GRB7
	0.78	CAB1	STARD3
Factor 5:			
	1.3	Intestinal trefoil factor	TFF3
	1.1	Clone AA314825:EST186646	-
	0.97	Clone AI985964:wr79d08.x1	-
	0.45	Secreted cement gland protein XAG-2 homolog	AGR2
	0.18	Cytochrome b5	CYB5
	0.16	Cytochrome b5	CYB5
Factor 8:			
	0.83	PRAD1 (cyclin D)	CCND1
	0.81	BCL-1 (cyclin D)	CCND1
	0.73	CYCD1 (cyclin D)	CCND1
	0.15	Cytochrome b5	CYB5

Table 1: Breast cancer hormonal pathways. Some of the genes significantly loaded on latent factors 1,3,5 and 8 in the ER/HER2 breast cancer data analysis.

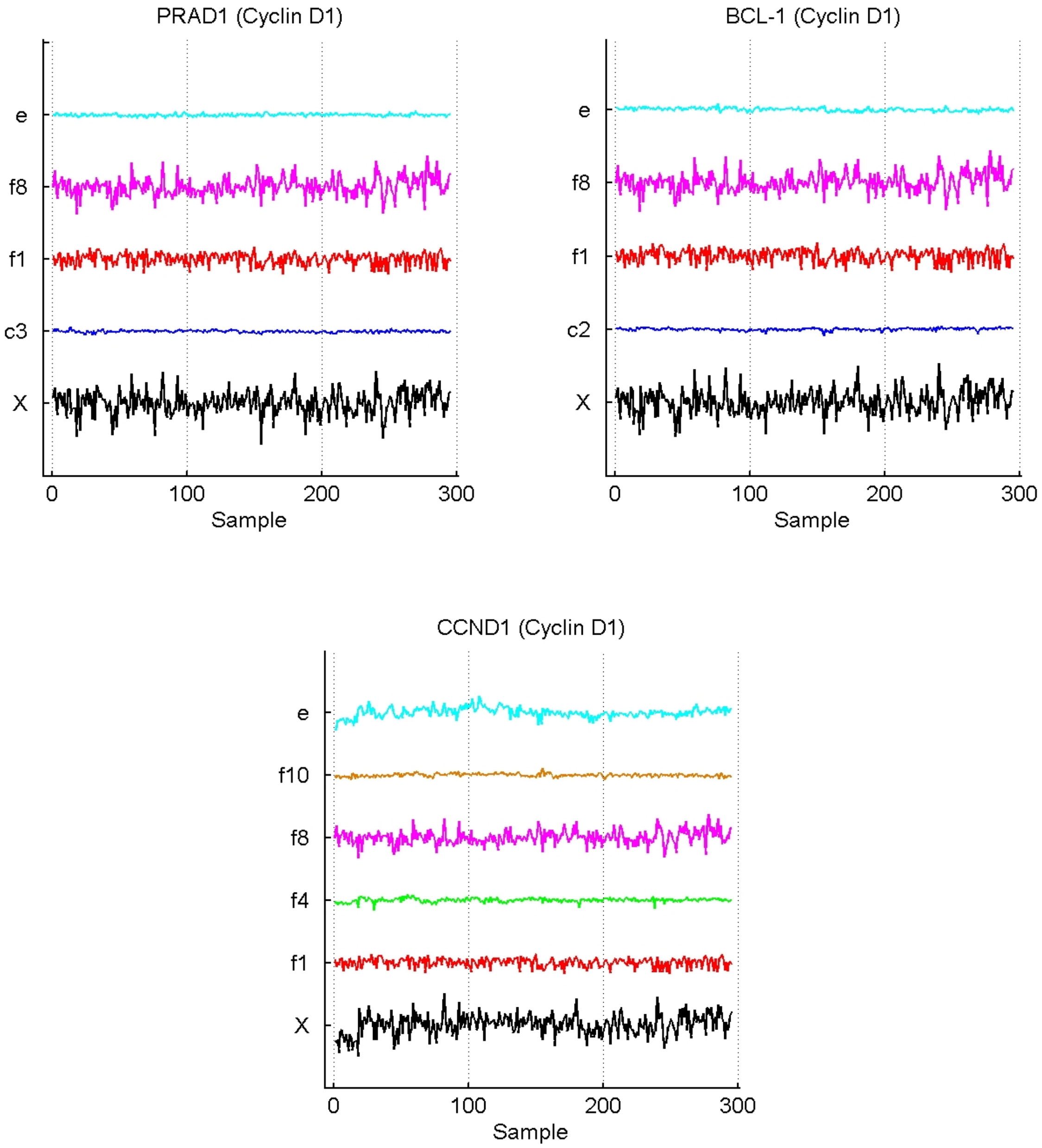

Figure 3: Breast cancer hormonal pathways. Plot across breast tumour samples of levels of expression (X) of the gene Cyclin D1. The upper left frame is the PRAD1/CCND1 probe set on the Affymetrix u95av2 microarray, one of the three probe sets for Cyclin D1 on this array; the upper right frame is the BCL-1/CCND1 probeset, and the lower frame that for the primary CCND1 probeset. Factors labelled f are primary latent factors, c indicates assay artifact covariates, and e represents the fitted residuals. In each of three frames, the plotted gene expression, factor and residual levels are on the same vertical scale within the frame, so indicating the breakdown of the expression fluctuations for Cyclin D1 gene probesets according to contributions from the factors. Factor 1 is the primary ER factor, and factor 8 a factor defined by the three probe sets for Cyclin D1, as discussed in text.

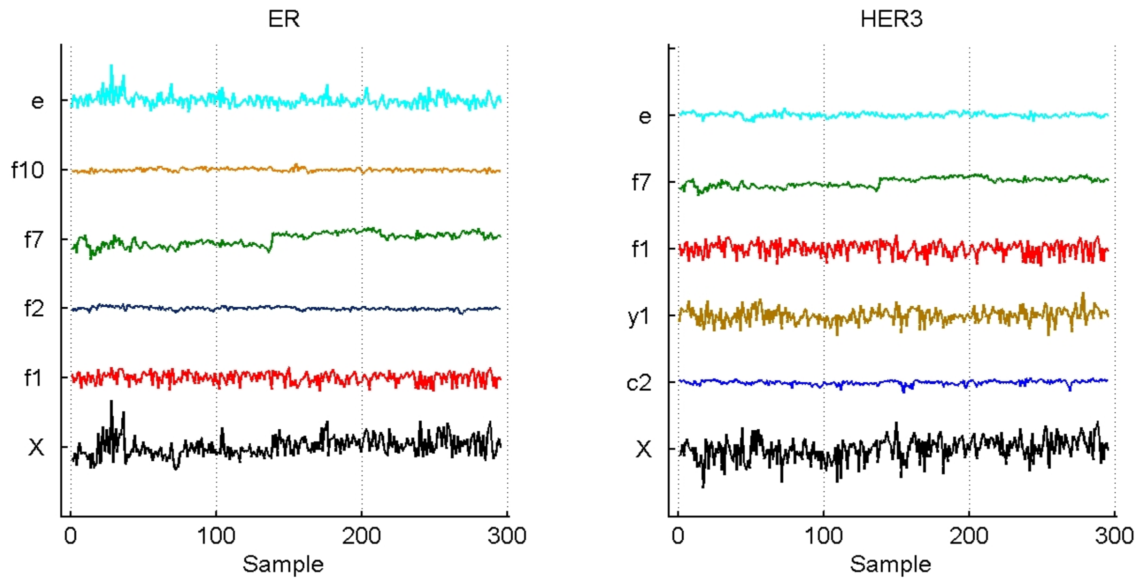

Figure 4: Breast cancer hormonal pathways. Plot across breast tumour samples of levels of expression (X) of the estrogen receptor (ER) gene and of the HER3 epidermal growth factor receptor tyrosine kinase, together with the estimates of factors contributing significantly to their expression fluctuations. Factors labelled f are primary latent factors, y indicates response factors, c indicates assay artifact covariates, and e represent the fitted residuals, and other layout details are as in Figure 3.

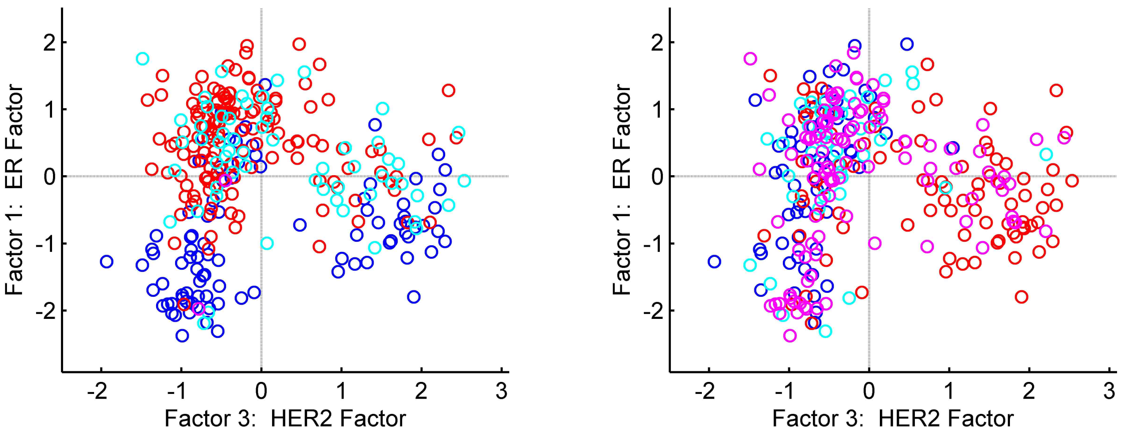

Figure 5: Breast cancer hormonal pathways. Scatter plot of the posterior means of designated ER factor 1 and HER2/ERB-B2 factor 4. Colour coding indicates the global measurement of protein level from IHC assays – Left frame: red ER+, blue ER−, magenta missing, cyan indeterminate. Right frame: red HER2+, blue HER2−, magenta missing, cyan indeterminate.

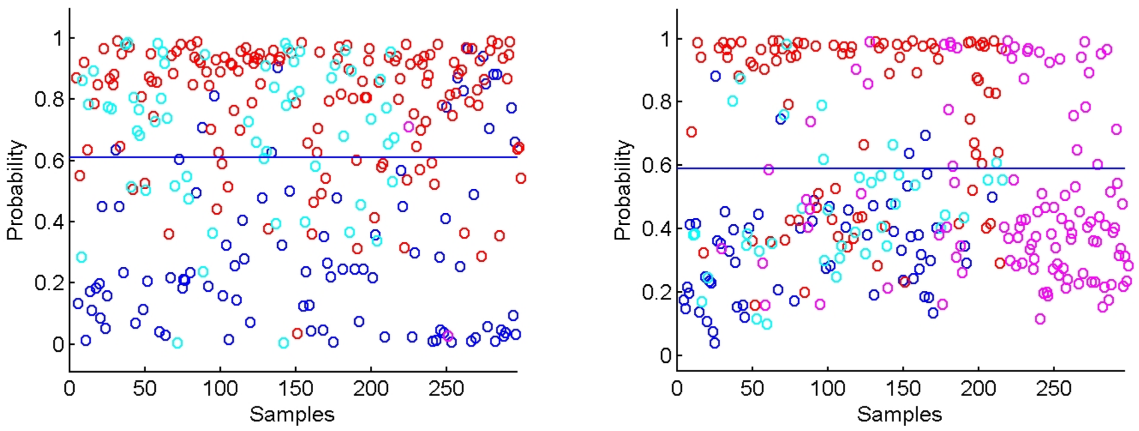

Figure 6: Breast cancer hormonal pathways. Scatter plot of fitted probabilities of ER+ and HER2+ from the overall factor regression model that includes probit components for these two binary responses. Color coding indicates hormonal receptor status as, in each case: red +, blue −, magenta missing, cyan indeterminate.

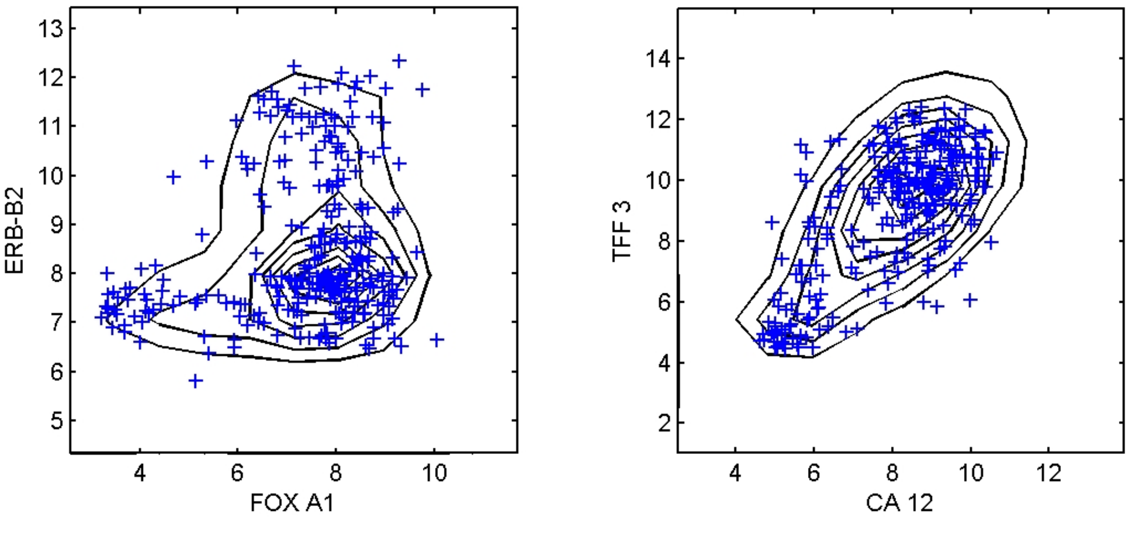

Figure 7: Breast cancer hormonal pathways. Plots display the approximate predictive density contours and observed data for two selected bivariate margins on four genes – HER2/ERB-B2 and the ER-related FOXA1, TFF3 and CA12 – with the observed data marked as crosses.

Factor	Top Genes	Function	p53 Status
1	CA12, TFF3, GATA3,<br>SPDEF, FOXA1	ER	Yes
2	PERP, E2F3, EP300,<br>BAX, RB1	cell development,<br>apoptosis	No
3	ESR1, SCUBE2, NAT1,<br>BCMP11, MAPT	ER	Yes
4	TOP2A, ASPM, CDC2,<br>RRM2, BUB1B	cell development,<br>apoptosis	Yes
5	ASPN, COL5A2, COL10A1,<br>COl3A1, COL6A2		No
6	CCL5, CXCL9, CXCL13,<br>LTB, TRGC2	Immunoregulation	Yes
7	FOS, JUN, EGR1,<br>EGR3, ATF3	cell development,<br>apoptosis	Yes
8	KRT14, KRT17, KRT5,<br>KRT6B, SFRP1		No
9	COL12A1, LTB4DH, CSPG2<br>MYC, RRM1		No
10	CDKN2A, SFN, SCUBE2,<br>CXCL10, BCL2	cell development,<br>apoptosis	No
11	AFF3, VTCN1, CPEB2<br>ENO2, PERP		No
12	CAV1, GPR116, TGFBR2<br>CAV2, PLVAP		No

Table 2: Breast cancer p53 study: Biological annotation for the latent factors defined by the model analysis. The p53 status column simply refers to the direct association between p53 mutational status and the posterior mean of the factor scores in a univariate model. See Figures 8 and 9 for some detailed investigation of the variation in factor levels across samples, and of their visual associations with the two binary ER (positive/negative) and p53 (mutant/wildtype) phenotypes.

Linear Predictor	Factor	$\hat{\pi}_{g,j}$	$\hat{\alpha}_{g,j}$
1: p53 Status	Response 1	1.000	0.111
	Factor 3	0.926	-0.347
	Factor 4	1.000	0.617
2: ER	Response 2	1.000	0.104
	Factor 1	0.953	-0.359
	Factor 3	0.910	0.3867
3: Survival	Response 3	1.000	0.089
	Factor 4	0.830	-0.345
	Factor 6	0.882	0.261

Table 3: Breast cancer p53 study. Coefficient probabilities and estimates for factors contributing to the linear predictors for the three response variables.

Response Factor	Top Genes	$\hat{\alpha}_{g,j}$
p53 Status	CSPG2	0.721
	ASPN	0.486
	COL3A1	-0.468
	COL3A1	-0.452
	COL1A1	-0.434
ER	EGR1	-0.226
	NPDC1	0.180
	EFEMP2	0.1607
	MCM4	0.156
	RBM5	0.968
Survival	COL5A2	0.380
	VIL2	0.342
	TRPS1	0.281
	TOP2A	0.272
	YY1	0.183

Table 4: Breast cancer p53 study. A few of the top genes and their estimated loadings on each of the three response factors.

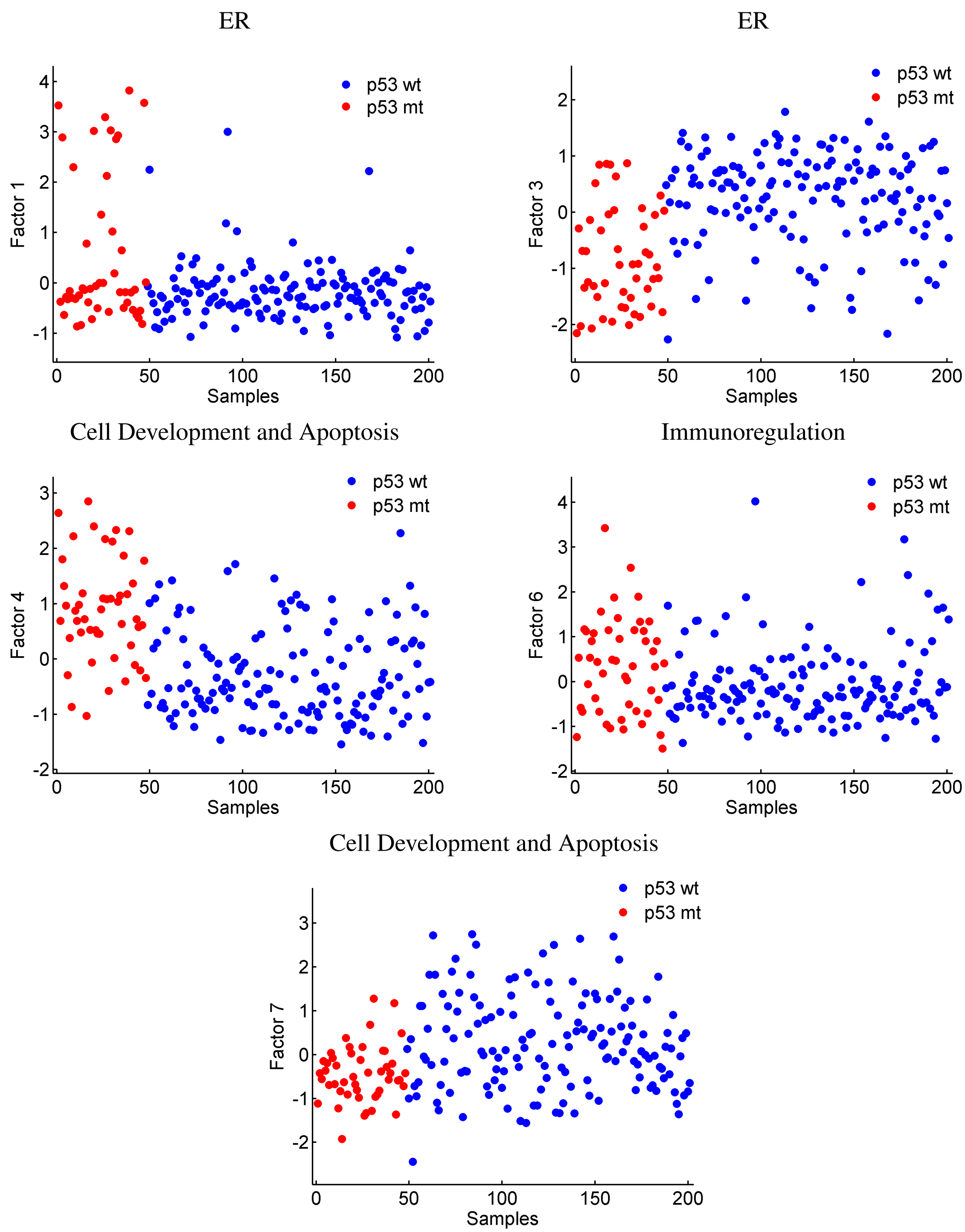

Figure 8: Breast cancer p53 study. Several of the estimated latent factors that show direct association with p53 Status (Table 2). Note also the interesting evidence of association between ER and p53 in some of these factors.

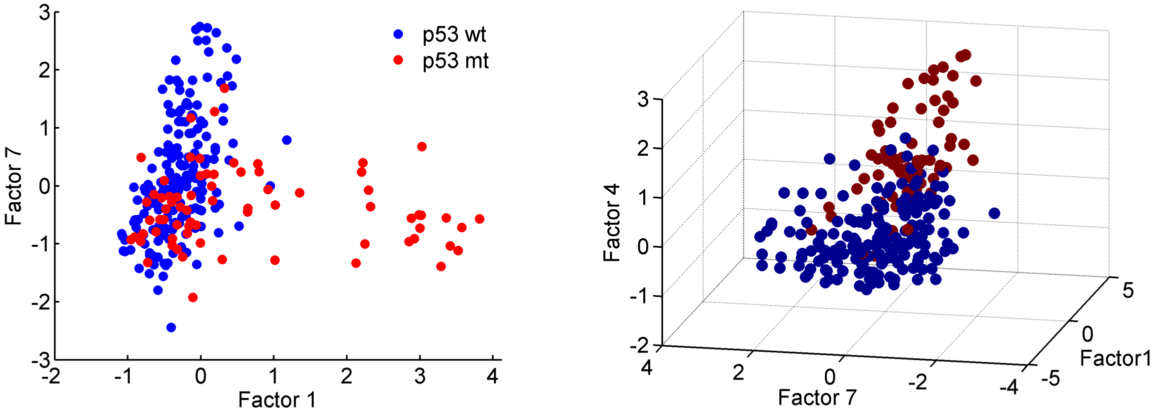

Figure 9: Breast cancer p53 study. Additional illustrative scatter plots of some of the key factors associated with p53 status.

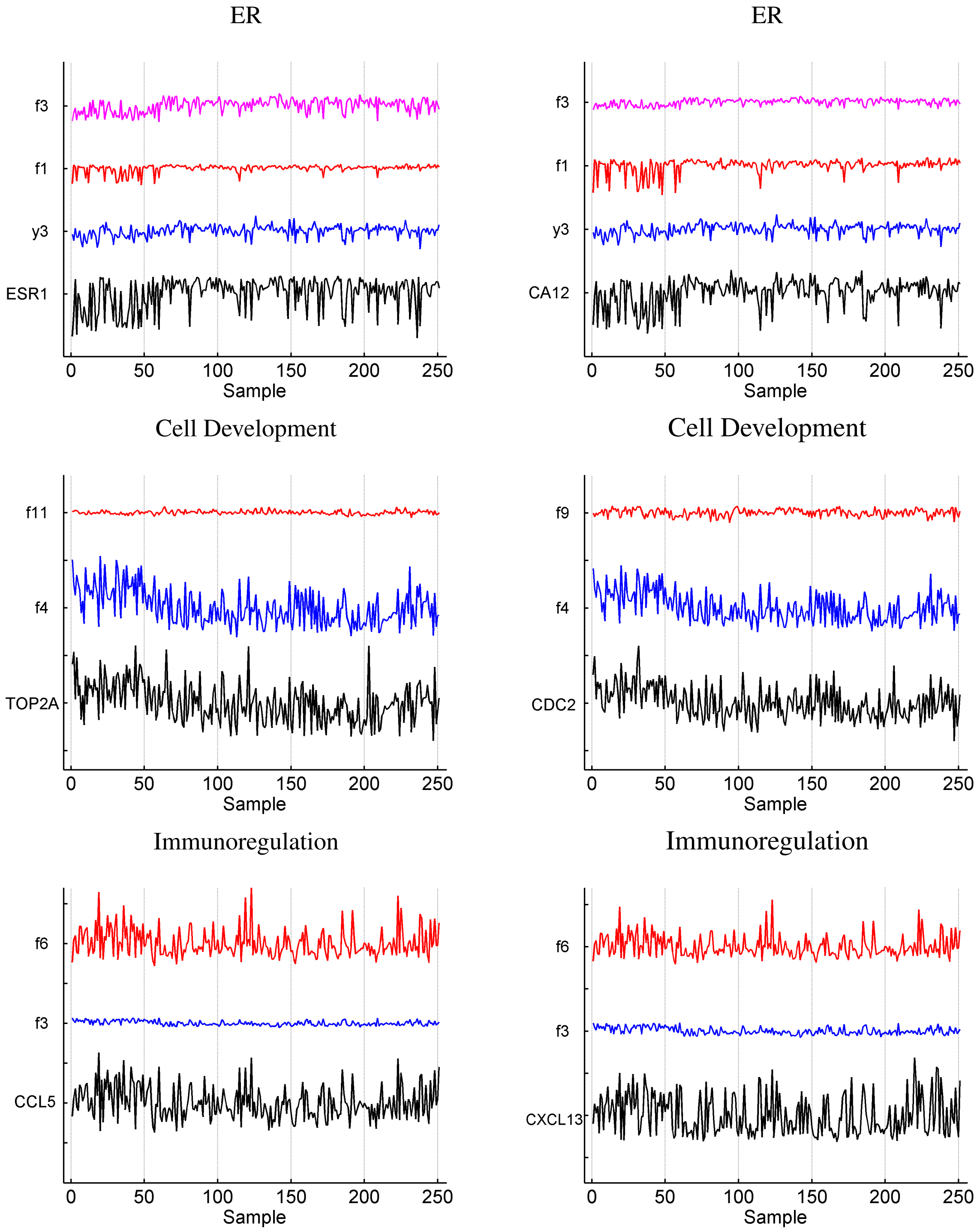

Figure 10: Decomposition of expression over samples of several top genes in the annotated ER factors 1 and 3, cell development factor 4 and immunoregulatory factor 6.

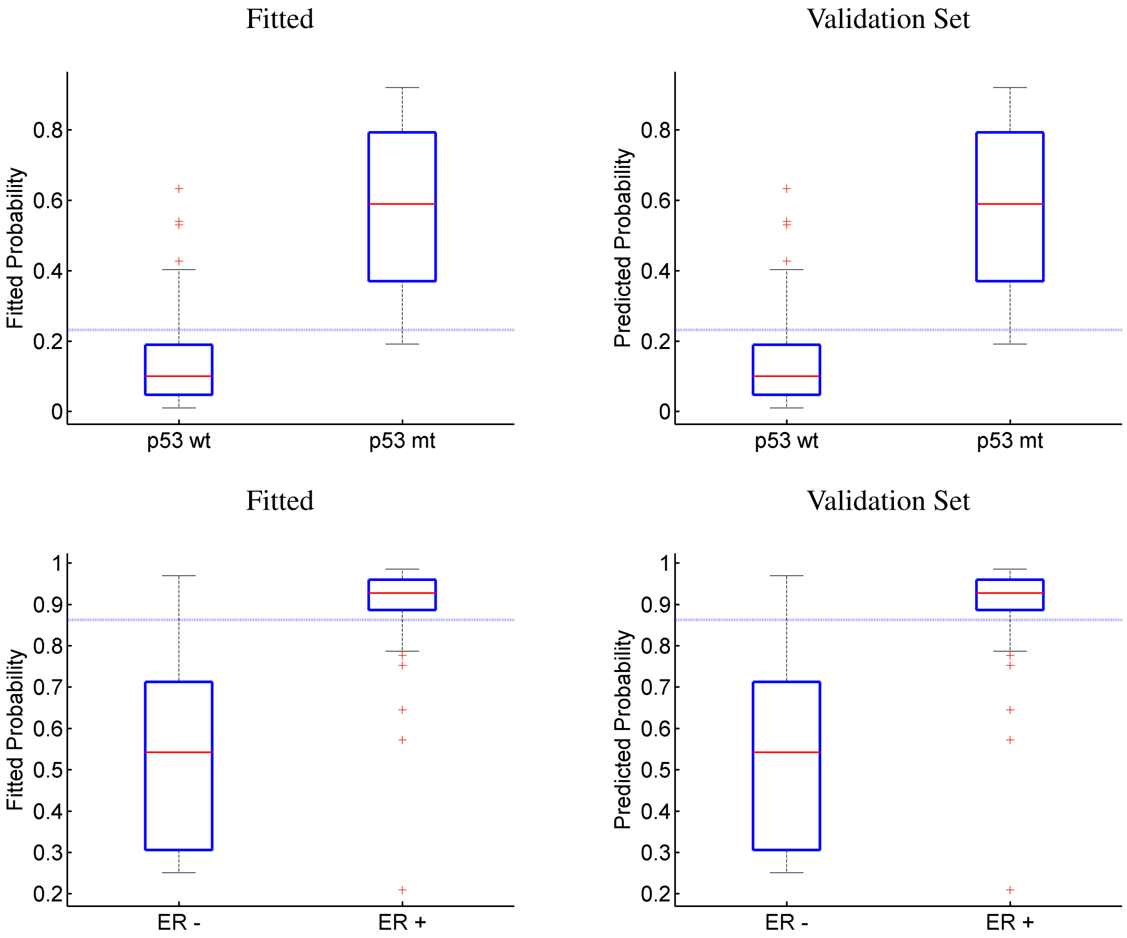

Figure 11: Breast cancer p53 study. Boxplots of fitted (left column) and out-of-sample predicted (right column) probabilities of p53 mutant versus wildtype (upper row) and ER$+$ versus $-$ (lower row).

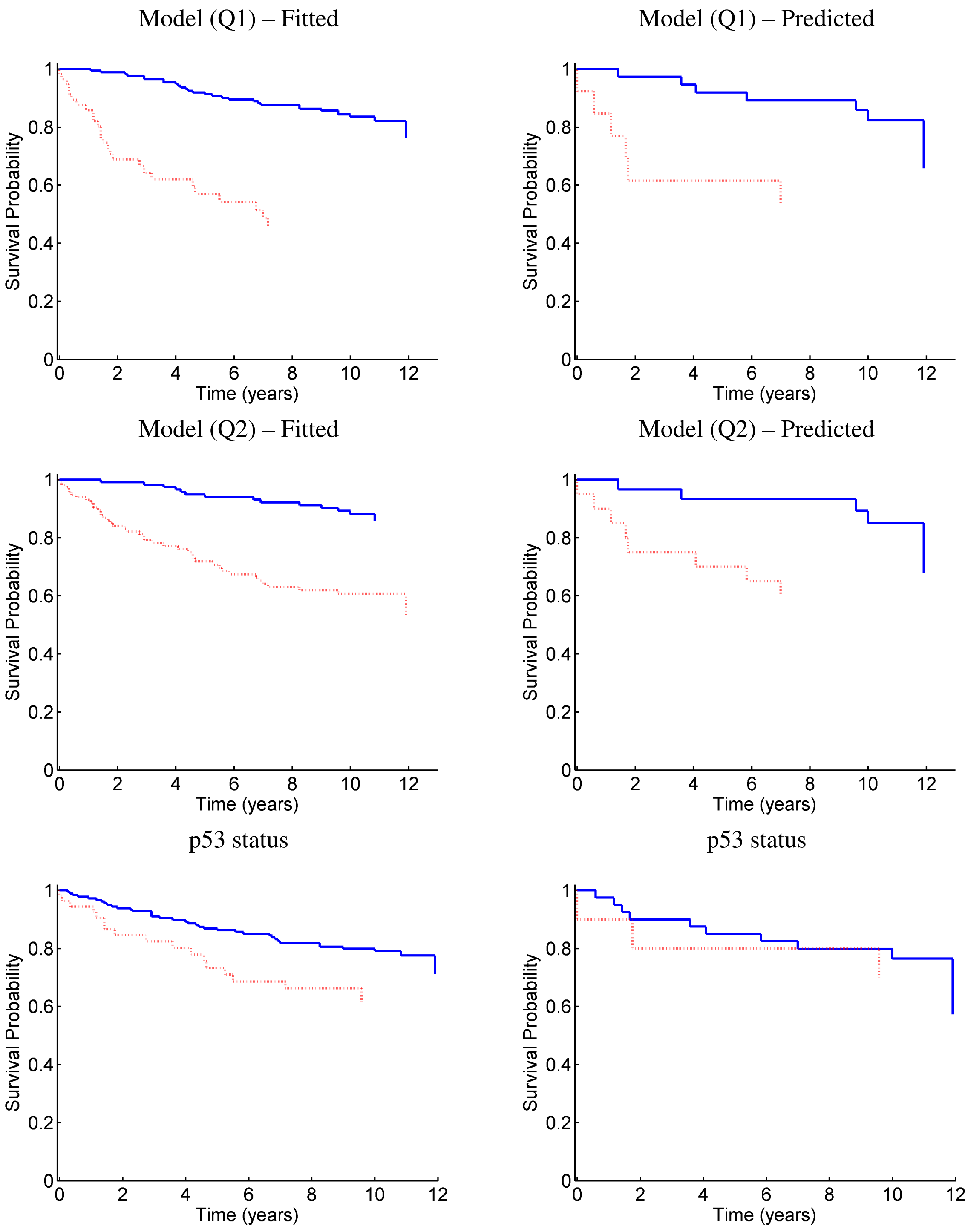

Figure 12: Breast cancer p53 study. Kaplan-Meier survival curves for the ($n = 201$ training and, separately $n = 50$ test) samples split according to the indicated thresholds. In the first and second rows, Q1 represents thresholding at the 1st quartile of the fitted or predicted linear predictor, and Q2 represents thresholding at the median. The lower two frames represent stratification simply on p53 wildtype versus mutant.
