# Supplementary material for: Cross-Study Projections of Genomic Biomarkers: An Evaluation in Cancer Genomics
Source: PLoS One. 2009 Feb 19;4(2):e4523. doi: 10.1371/journal.pone.0004523 (PMC2638006; doi:10.1371/journal.pone.0004523)
Supplement: Supplemental Materials S2 — In-vitro to in-vivo factor profiling in expression genomics. Reference 37 is currently in press, so we have included it as supplimentary material. (0.73 MB PDF) [file pone.0004523.s007.pdf]

Contents

I Part 1 **7**

1 In-Vitro to In-Vivo Factor Profiling in Expression Genomics 11

Joseph E. Lucas[1]*, Carlos M. Carvalho*[2]*, Daniel Merl*[1] *& Mike West*[1] [1]Duke University, [2]University of Chicago

1.1 Introduction 11

1.2 Modeling Gene Expression 13

1.2.1 Sparsity Priors 14

1.2.2 Signature Scores 14

1.2.3 Sparse, Semi-Parametric Latent Factor Models 16

1.3 Signature Factor Profiling Analysis 17

1.4 The E2F3 Signature in Ovarian, Lung, and Breast Cancer . 19

1.4.1 Indications of E2F3 Co-Regulation with NF-Y 20

1.4.2 Adenocarcinoma versus Squamous Cell Carcinoma . . 21

1.4.3 Survival and the E2F3 Pathway 21

1.5 Closing Comments 23

References **25**

0-8493-0052-5/00/$0.00+$.50
© 2001 by CRC Press LLC

List of Tables

0-8493-0052-5/00/$0.00+$.50
© 2001 by CRC Press LLC

List of Figures

1.1 Scatter plot of estimated values $\lambda^*_{1,i}$ in the ovarian cancer data set (x-axis) against scores on the ovarian tumors of the lung and breast cancer factors 1; these two sets of scores are constructed by projection of the estimated factor loadings of lung and breast factor 1 into the ovarian data set, as described in section 1.2.3. 29

1.2 A Venn diagram depicting intersections of the gene lists of factor 1 from breast, lung, and ovarian cancers (total number of genes in each factor listed outside the circles, the number of genes in each intersection listed inside). Although the ovarian cancer factor 1 significantly involves a smaller number of genes that the other cancers, almost all of these (109 of 124) are common with those in the lung and breast factors. 30

1.3 Patterns of expression of the 109 genes in the intersection group in Figure 1.2. The genes are ordered the same in the three heatmap images, and the patterns of expression across samples are evidently preserved across genes in this set. 31

1.4 Factors 4 and 5 from the E2F3 signature factor profile in the lung cancer data set show a clear ability to distinguish between adenocarcinoma and squamous cell carcinoma. The figure displays probabilities of squamous versus adenocarcinoma from binary regression models identified using SSS. 32

1.5 Patterns of expression of the genes in factor 4 in lung cancer. This factor shows differential expression of 350 genes that distinguish between squamous cell carcinoma and adenocarcinoma. It is therefore appropriate that the pattern of expression is clearly not conserved in the ovarian and breast cancers. . . 33

1.6 Kaplan-Meyer curves representing survival in breast cancer patients with above/below median scores for the E2F/NF-Y factor. Suppression of the E2F/NF-Y pathway appears to be associated with the decreased probability of long term survival. 33

1.7 Kaplan-Meyer curves representing survival in ovarian cancer patients with above/below median scores for the E2F/TFDP2 factor. Suppression of the E2F/TFDP2 pathway appears to be associated with decreased probability of long term survival. . 34

0-8493-0052-5/00/$0.00+$.50
© 2001 by CRC Press LLC

1.8 Kaplan-Meyer curves representing survival in lung cancer patients with above/below median scores for the E2F/ELF1 factor. Suppression of the E2F/ELF1 pathway appears to be associated with decreased probability of long term survival. . . 35

Part I

Part 1

Symbol Description

i — An index for the list of samples from a microarray experiment (e.g. a particular microarray).

g — An index for the list of probe IDs – i.e., genes – on the expression array.

$x_{g,i}$ — (log base 2) Gene expression for gene g and sample i.

x_i — $p-$vector of the $x_{g,i}$.

X — $p \times n$ matrix whose columns are the x_i.

H — Transpose of the $n \times r$ design matrix in a designed experiment.

h_i — The ith column of H corresponding to the ith sample.

B — A $p \times r$ matrix of regression coefficients, where r is the number of elements in the design matrix and p is the number of genes – or, more properly, probe sets – on the array.

Λ — $k \times n$ matrix of latent factors.

λ_i — The ith column Λ.

A — A $p \times k$ matrix of factor loadings, where k is the number of latent factors.

ψ_g — Residual variance of the expression measurement error for gene g.

$\pi_{g,j}$ — Sparsity probability governing non-zero values of (g, j) elements of A and B.

$\circ^*$ — For any quantity $\circ$, the MCMC-based approximation to the posterior mean $E(\circ|X)$.

Chapter 1

In-Vitro to In-Vivo Factor Profiling in Expression Genomics

Joseph E. Lucas[1], Carlos M. Carvalho[2], Daniel Merl[1] & Mike West[1]
[1]*Duke University,* [2]*University of Chicago*

1.1 Introduction 11
1.2 Modeling Gene Expression 13
1.3 Signature Factor Profiling Analysis 17
1.4 The E2F3 Signature in Ovarian, Lung, and Breast Cancer 19
1.5 Closing Comments 23
Acknowledgements 24

1.1 Introduction

The widespread use of DNA microarray gene expression technology offers the potential to substantially increase our understanding of cellular pathways and apply that knowledge in clinical as well as basic biological studies. In human biology, there are two common types of study performed with expression arrays. The first involves samples derived from cloned cells in a highly controlled environment, subject to some designed experimental intervention, such as up/down regulation of a particular target gene [1, 2, 3] or manipulation of the pH level of the growing environment [4]. Such experiments are useful in that they are designed to minimize all sources of gene expression variability except that of the experimental intervention. One result generated by this type of experiment is the identification of a subset of genes that are differentially expressed when subject to a given intervention. A list of such genes, together with the magnitudes by which those genes are differentially over/under-expressed and additional numerical summaries are said to comprise the *signature* of the intervention. The second, common type of study involves samples derived from tissue removed from living subjects, such as tumor tissue obtained via biopsy [5, 6, 7, 8, 9, 10, 11, 29]. A central goal of this second type of study is to find statistical associations between the measured expression levels in a tissue sample and clinical variables associated with the subject, e.g. survival time or metastasis.

Much of our interest in recent and current studies lies in relating these two

0-8493-0052-5/00/$0.00+$.50
© 2001 by CRC Press LLC

types of experiments. We concentrate in this chapter on studies in cancer genomics, though the general questions of cross-study projection of genomic markers, and the more general questions of trans-study and trans-platform integration of genomic data and analysis results, are central to all human disease studies with genomic methodologies (e.g., [13]). In cancer genomics, for example, a designed *in vitro* experiment in cultured cells may identify a list of genes that are differentially expressed in the presence of an up-regulated oncogene; we might then focus on data on the same genes in a collection of tumor tissue samples to determine the extent to which each tumor exhibits signs of de-regulation of that oncogene. One way to accomplish this is to make use of the oncogene signature derived from the controlled study to compute an appropriately weighted average of the relevant gene expression levels observed in the tumor tissue samples. This technique has been shown to be useful for quantifying pathway activity in a variety of different settings (e.g., [3]).

However, there are at least two difficulties with this approach. First, it is exceedingly rare to find that the exact patterns of differential expression discovered in the designed experiment are conserved in the tissue samples. Second, the highly controlled setting of the designed experiment results in a very limited representation of the various sources of variability in gene expression that are likely to affect expression in tissues grown *in vivo*. Returning to the example of up-regulation of a particular gene, consider an example of the MYC gene (myelocytomatosis viral oncogene homolog) in mammalian cell developmental processes. MYC is a transcription factor that is well-known to be involved in programmed cell death, cellular differentiation, proliferation, and potentially a number of other biological pathways. Myc is not, however, the only gene associated with each of these pathways, and therefore a tissue sample which exhibits high levels of expression of MYC may not exhibit high levels of activity of all of its associated downstream pathways due to the activity of other genes, especially other transcription factors. For the same reason, there will be genes in the MYC pathways that are not discovered by the simple MYC upregulation experiment due to low levels of expression of co-regulators. In short, the designed experiment lacks information pertaining to possible synergistic or antagonistic activity between MYC and other genes of the sort likely to be encountered *in vivo*.

Translation of patterns of differential gene expression derived from *in vitro* experiments to *in vivo* tissue studies therefore requires a methodology for decomposing an experimental signature into functional sub-units, each reflecting modular but interacting components of a larger cellular process. This chapter overviews and exemplifies our approach to this general problem area using sparse factor models, an approach that has been used effectively in a number of applications in cancer genomics and other contexts. Here we focus on the application to trans-study analyses connecting *in vitro* expression discovery to *in vivo* contexts in cancer. Based on formal Bayesian models for both contexts, we describe methodology for projection and then evolving experimentally derived signatures into sets of interacting sub-signatures, as

represented by multiple latent factors in formal latent factor models of observational human cancer data. We demonstrate how latent factors can be linked to components of known cellular pathways, as well as how latent factors can be predictively related to clinical outcomes. Issues of data calibration and other practicalities are also addressed in this formal factor model framework for *in vitro* to *in vivo* translation of biological markers.

1.2 Modeling Gene Expression

In general, for experiments performed on cloned cells under strictly controlled conditions, our models utilize multivariate regression and analysis of variance with a fixed, known design to describe observed expression patterns. We use the notation of [15]. Suppose gene expression assay has p genes (probe sets on a DNA microarray) and we perform the experiment on n samples, and let X be the $p \times n$ data matrix of observed expression values with elements $x_{g,i}$. Let the $r \times n$ matrix H be the transpose of the design matrix; so H has elements $h_{j,i}$ running over treatments/factors $j = 1 : r$ (rows) and expression array samples $i = 1 : n$ (columns). We model the measured expression values (on a log base 2, or fold-change scale) as:

$$x_{g,i} = \mu_g + \sum_{j=1}^{r} \beta_{g,j} h_{j,i} + \nu_{g,i}, \qquad \nu_{g,i} \perp\!\!\!\perp N(\nu_{g,i}|0, \psi_g)$$

or, in vector form,

$$x_i = \mu + Bh_i + \nu_i, \qquad (i = 1 : n),$$

where μ is the $p-$vector of baseline average expression levels μ_g for all genes, B is the $p \times r$ matrix of regression coefficients with elements $\beta_{g,j}$, h_i is the $r-$vector column i of H, ν_i is the $p-$vector of gene-specific experimental noise terms $\nu_{g,i}$ with individual normal error variances ψ_g and $\Psi = \text{diag}(\psi_{1:g})$. In matrix form,

$$X = \mu 1' + BH + N$$

where 1 is the $n-$vector of 1s and N the $p \times n$ error matrix with columns ν_i.

Depending on the experimental context, the rows of H will include 0/1 entries reflecting main effects and interactions of treatment factors, genetic or environmental interventions and so forth, with the corresponding $\beta_{g,j}$ values representing the changes in average expression for each gene g as a function of *design factor* j. The design matrix may also include measured values of regressor variables. In most of our studies, we generally include values of sample-specific control information constructed from housekeeping probe sets

on the array; these covariates carry *assay artifact* information reflecting experimental bias and errors that are often reflected at least sporadically, if not in some cases substantially, across the samples. Ignoring the potential for such assay artifacts to corrupt expression on a gene-sample specific level can lead to false discovery and/or obscure biological variation, and our approach to gene-sample specific normalisation using housekeeping gene data is generally applicable to at least partially address this in the automatic analysis [14, 15, 16]. These references also describe model fitting and evaluation under specified priors, and describe – with a number of examples – the MCMC implementation in the Bayesian Factor Regression Models (BFRM) software, available at the Duke Department of Statistical Science software web page.

1.2.1 Sparsity Priors

In the case of very high-dimensional genomic assays, we expect that most genes will not show differential expression in association with any particular design factor - biology is complex, but not that complex. That is, any column j of B will be sparse, with typically many, many zeros. Our studies over the last several years have demonstrated the importance of sparsity prior modeling utilising the class of prior distributions [14] that reflect this inherent biological view that, in parallel for all $j = 1 : r$, *many genes g have zero probability of a non-zero $\beta_{g,j}$*. That is, the priors utilise the standard Bayesian zero point-mass/mixture form in which each $\beta_{g,j}$ has an individual probability $\pi_{g,j} = Pr(\beta_{g,j} \neq 0)$, but now the extension allows some (typically many) of the $\pi_{g,j}$ themselves to be zero, inducing a more aggressive shrinkage of the $\beta_{g,j}$ to zero. Specifically, the hierarchical sparsity prior model is (see [14])

$$\begin{aligned}
\beta_{g,j} &\sim (1-\pi_{g,j})\delta_0(\beta_{g,j}) + \pi_{g,j} N(\beta_{g,j}|0,\tau_j),\\
\pi_{g,j} &\sim (1-\rho_j)\delta_0(\pi_{g,j}) + \rho_j Be(\pi_{g,j}|a_j m_j, a_j(1-m_j)),
\end{aligned}$$

with hyper-priors on the elements ρ_j and τ_j. We refer to such distributions interchangeably as sparsity priors and variable selection priors. Our assumption is that the probability of any particular probe associating with a given design factor is quite low, so that we assign the priors $Be(\rho_j|st, s(1-t))$ with s is large and t is small. The priors on the τ_j, conditionally conjugate inverse gamma priors, are specified in context of the design and, critically, the know range of (log base 2) expression data (see many details and examples in [14, 15, 16] and the BFRM software web site linked to the latter paper, under the Duke Department of Statistical Science software web page).

1.2.2 Signature Scores

Assume a model has been fitted to a given experimental data set, generating posterior summaries from the MCMC computations. We can explore, characterize and summarise significant aspects of changes in gene expression

expression with respect to design factors in a number of ways. Of particular interest in studies that aim to connect the results of such *in vitro* studies to data from human observational studies is the definition and evaluation of a summary numerical *signature score* for any set of genes that are taken as defining a signature of one of the design factors.

Suppose that for the design variable j there are a number of genes with very high values of $\pi^*_{g,j}$; one typical approach is to threshold the probabilities to identify a set of most highly significant genes, and then define a weighted average of expression on those genes as a signature score. In a number of studies we have used this approach with the weightings defined as follows.

Consider a potential future sample x_{new} with a fixed and known value h_{new}. For each g, j and conditional on all model parameters, the likelihood ratio $p(x_{g,new}|\beta_{g,j} \neq 0)/p(x_{g,new}|\beta_{g,j} = 0)$ depends on $x_{g,new}$ monotonically and only through the term

$$s_{g,j} x_{g,new} \quad \text{with} \quad s_{g,j} = \psi_g^{-1} \beta_{g,j}.$$

Hence the future observation will be more consistent with non-zero effects on design factor j for larger values of the sum of these terms over all genes g, the sum being implied by the conditional independence of the $x_{g,\cdot}$ given all model parameters. This leads to the obvious *signature score* for design factor j as

$$sig_{j,new} = \sum_{g=1}^{p} s_{g,j} x_{g,new} = s_j' x_{new}$$

for the signature j weight vector $s_j = (s_{1,j}, \ldots, s_{p,j})'$. In practice, we can estimate the signature weight vectors s_j by posterior estimates based on the MCMC samples of the observed data, and use these to evaluate the score on any future samples – i.e., to project the signature measure of the activity levels of genes related to design factor j onto the new sample, predictively. This can be done within the MCMC analysis by saving and summarising posterior samples of the set of s_j, or as an approximation after the analysis by substituting posterior estimates for the parameters in the expression for $s_{g,j}$, such as by plugging-in posterior means $\pi^*_{g,j} = Pr(\beta_{g,j} \neq 0|X)$, posterior estimates of residual variance ψ^*_g for each gene g, and posterior estimates of effects $B^* = (\beta^*_{g,j})$ where $\beta^*_{g,j} = E(\beta_{g,j}|X, \beta_{g,j} \neq 0)$. This latter approach leads to the defined signature scores $sig^*_{j,new}$ used here, in which $s_{g,j}$ is estimated by

$$s^*_{g,j} = \pi^*_{g,j} \beta^*_{g,j} / \psi^*_g.$$

Further, we may modify this definition to sum over just a selected set of signature genes for which $\pi^*_{g,j}$ exceeds some specified, high threshold. This is important for communication and transfer to other studies, as it generates a more manageable subset of signature genes rather than requiring the full set.

1.2.3 Sparse, Semi-Parametric Latent Factor Models

Some of our major applications in recent studies of observational data sets in cancer, as in other areas, have utilised large-scale Bayesian latent factor models [17, 18], either alone or as components of more elaborate *latent factor regression models* [14, 15]. The value of latent factor models to reflect multiple intersecting patterns underlying observed variations in gene expression data across samples of, for example, lung cancers is clear. It is also relevant in some experimental contexts. The model as described above assumes that all sources of variation in expression are known and represented by the fixed design, though imprecision in the design specification and other aspects of biological activity may lead to influences on expression reflected in common patterns in multiple subsets of genes that may be captured by latent factors.

The extension of the model to include latent factors is simply

$$X = \mu 1' + BH + A\Lambda + N$$

where the $k \times n$ matrix Λ represents the realised values of k latent factors across the n samples, having elements $\lambda_{j,i}$ for factor $j = 1 : k$ on sample $i = 1 : n$. Λ is a now uncertain analogue to the known matrix H from the design. The columns of the $p \times k$ factor loadings matrix $A = \alpha_{g,j}$ are the weights or coefficients of genes on factors (analogous to the regression coefficients in B). In observational cancer studies with known covariates, we will generally use the full model above; in other studies, either of the design or factor component may be absent, depending on context.

The elements of A are given sparsity priors as described for $\beta_{g,j}$. Our models for latent factor space utilise non-parametric components to allow for what may be quite non-Gaussian patterns in these common, underlying patterns that reflect underlying "gene pathway" activities influencing gene expression. It is common to observe genes in subsets that are unexpressed in some samples but clearly expressed at possibly various sets of levels across others, for example; subtle examples include expression in the presence/absence of a genetic mutation, in diseased versus non-diseased patients, or before and after treatment. In these situations, and others, a standard normal latent factor model would be lacking. Our models assume a Dirichlet process component for this. With λ_i representing the $k-$vector column of Λ, we have

$$\begin{aligned} \lambda_i &\sim F(\cdot), \\ F &\sim DP(\alpha F_0), \\ F_0(\lambda) &= N(\lambda|0, I) \end{aligned}$$

with total mass $\alpha > 0$. This encourages latent factor-space clustering of the samples with relation to gene expression patterns and can lead to cleaner separation when expression differences are due to binary or categorical phenotypes when appropriate, as well as allowing for adaptation to multiple other

non-Gaussian aspects. The model and MCMC computational extensions are routine and implemented in BFRM [16].

Consider now a potential future observation, x_{new} at a known design vector h_{new}, and set $z_{new} = x_{new} - BH$. Conditional on all model parameters and any realised Λ values (the full set of conditioning values being simply denoted by $\circ$ below) it then routinely follows that

$$(\lambda_{new}|z_{new}, \circ) \sim c_{new}N(\lambda_{new}|d, D) + \sum_{i=1}^{n} c_i \delta_{\lambda_i}(\lambda_{new}),$$

where $\delta_e(\cdot)$ is the Dirac delta function representing a point mass at e; the c_q are probabilities given by defined by

$$c_{new} = \alpha CN(z_{new}|0, AA' + \Psi),$$
$$c_i = CN(z_{new}|A\lambda_i, \Psi), \quad i = 1 : n$$

where C is a constant of normalisation; and

$$d = DA'\Psi^{-1}z_{new} \quad \text{and} \quad D^{-1} = I + A'\Psi^{-1}A.$$

We know, incidentally, that the λ_i cluster into subsets of common values, and hence the sum above collapses to a simpler sum over the distinct values; that fact and its implications is central to efficient MCMC computation though not immediately relevant for this specific theoretical discussion.

As with *in vitro* defined signature projection, we are often interested in predicting factor variability in a new sample, and the above distributions show how this is done. Given full posterior samples of the model parameters – now including Λ – based on the observed data X, we can at least approximately evaluate the terms defining the predictive distribution for λ_{new}. Plugging-in posterior estimates of model quantities as a practicable approximation leads to the ability to simulate λ_{new}, and also to generate point estimates such as those used in our examples below, viz.

$$\lambda^*_{new} = c^*_{new}d^* + \sum_{i=1}^{n} c^*_i \lambda^*_i \tag{1.1}$$

where λ^*_q are the MCMC posterior means of the λ_i and the the terms $c^*_\cdot$ and d^* are as theoretically defined above but now evaluated at posterior estimates of the model parameters and latent factors set at posterior means.

1.3 Signature Factor Profiling Analysis

Signature Factor Profiling Analysis (SFPA) refers to the overall enterprise of evaluating *in vitro* defined gene expression signatures of biological or environmental interventions, projecting those signatures into observational data

sets – such as a sample of arrays from breast cancer tissues – and then using latent factor models to explore and evaluate the expression patterns of signature genes, and other related genes, in the cancer data set. The complexity of *in vivo* biology compared to controlled experiments on cultured cells inevitably means that projected signatures involve genes that are simply not expressed in the cancer samples, and others that show far more complex and intricate patterns in the cancer samples. Thus factor analysis of a signature gene set can generate multiple (k) factors that reflect this increased complexity; this represents the $k-$dimensional *in vivo profile* of the one-dimensional *in vitro* signature.

Our factor modelling uses the evolutionary computational method for model search and gene inclusion that was introduced and described in [15]. Beginning with a small set of signature genes, MCMC analysis is used to fit an initial factor model, evolving the model in terms of the number of latent factors included. At the next step the model predicts outside that initial set of genes to evaluate all other (thousands of) genes on the array to assess concordance in expression of each of these genes with estimated latent factors in the "current" model. Of those genes showing high concordance, we can then select a subset to add to the initial set of genes and refit the factor model, potentially increasing the number of factors. Many examples are given in the references. In the current context, the biological focus is perfectly reflected in this evolutionary analysis: we are initally concerned with the "simple" expression signature of intervention on a single biological pathway – the design factor in question elicits that pathway response *in vitro*. When moving into observational cancer samples, biological action involving multiple other, intersecting pathways will generally be evident in the tumour data set. Thus (i) we will be interested in identifying other genes that seem to show patterns of related expression; and (ii) as we add in such additional genes, we are moving out of the initial signature pathway into intersecting pathways that will lead to the need for additional latent factors to reflect the increasingly rich and complex patterns observed. Operationally, this evolutionary process is allowed to continue until a certain number of factors has been found, until a certain number of genes have been added, or until no more factors or genes can be added based on thresholds on estimated posterior gene-factor loading probabilities (for details, see [15]). By restricting the termination requirements, we can control how closely the final list of factors remains to the initial set of probes, or how rich it can in principle become by exploring the biological neighbourhood of the original *in vitro* response.

1.4 The E2F3 Signature in Ovarian, Lung, and Breast Cancer

The E2F family of transcription factors is central to cell cycle regulation and synthesis of DNA in mammalian cells, playing roles in multiple aspects of cell growth, proliferation and fate [19, 20]. Deregulation of E2F family members is a feature of many human cancers [21]. Two of the family members, E2F1 and E2F3, are among the genes investigated in experiments defining *in vitro* signatures of oncogene deregulation in human mammary epithelial cells (HMEC) [3]. In these oncogene experiments, expression profiles were collected from cloned HMECs with each experimental group consisting of replicate observations from the same cloned HMECs after transfection with a viral plasmid containing a known oncogene. A series of control samples were also generated; see [14] for additional insights and examples using one-way Anova models under sparsity priors, as well as for discussion of the extension of this analysis to include assay artifact control regression variables. The nine oncogenes transfected were MYC, Src, Akt, P110, E2F1, E2F3, Ras, β-catenin, and p63. From among these, we focus here on E2F3 and explore the E2F3 signature projection to human cancers to exemplify signature factor profiling. The analysis generates biological connections in the E2F pathways consonant with known biology, as well as new and novel biological insights relevant to the oncogenic role of E2F3 when deregulated.

To define our initial signature gene set for evolutionary factor analysis, we reanalysed the full set of oncogene expression data (Affymetrix u133) in a one-way Anova design under sparsity priors. For design factor $j = E2F3$, the $\beta_{g,E2F3}$ then represent the average changes in expression on genes g due to upregulation of E2F3. We identify those probe sets g for which $\pi^*_{g,E2F3} > 0.99$ in this analysis.

Our interest here is in projecting the E2F3 signature to three different human cancer data sets: lung cancer [3], ovarian cancer [22], and breast cancer [11], also all on Affymetrix arrays. Of the initially screened genes, we first identified all of those probsets on the Affymetrix arrays used in each of the cancer studies; from among these, we then selected the 100 genes with the largest positive expression changes $\beta^*_{g,E2F3}$ in the experimental context. This defined our *in vitro* E2F3 signature gene set and the corresponding signature weight vector s^*_{E2F}.

Evolutionary factor analysis was applied to each of the three cancer data sets separately, initialising based on this signature gene set. The analyses evolved through a series of iterations, at each stage bringing in at most 25 additional genes most highly related to the "current" estimates of latent factors, and then exploring whether or not to add additional latent factors re-fitting the model and evaluating the estimated gene-factor loadings $\pi^*_{g,j}$ for all genes g and factors j in the model. Thresholding these probabilities at 0.75 was

used for both new gene inclusion and adding factors; an additional factor was added to the model only when at least 15 genes showed $\pi^*_{g,j} > 0.75$. The evolutionary analysis was run in the context of allowing expansion to no more than 1000 genes and 20 factors. The analyses terminated with $(p = 1000, k = 15)$ for lung, $(p = 1000, k = 14)$ for ovarian and $(p = 1000, k = 13)$ for breast cancer. Consistent with our strong belief that factors represent small collections of genes, the prior on ρ_j was a beta distribution with mean .001 and prior observations 1000. Both τ_j and Ψ_j were given diffuse inverse gamma priors.

In the remaining discussion, we describe some aspects of the resulting factor analyses of the three data sets and explore how these signature factor profiles relate across cancers and also relate to some underlying biological pathway interconnections. We refer to posterior means $\lambda^*_{j,i}$ as, simply, factor j or, the value of factor j, on sample i in any data set. Referring to the full vector of factors on sample i implicitly denotes the approximate posterior mean λ^*_i. Further, in discussing genes related to a specific factor we refer to genes being involved in the factor, or significantly loaded on the factor, based on $\pi^*_{g,j} > 0.99$. in the corresponding analysis.

1.4.1 Indications of E2F3 Co-Regulation with NF-Y

To begin, Figure 1.1 shows the high correlation between the first factor discovered in the ovarian cancer data (x-axis) compared to projections into the ovarian data of the first factors from each of the lung and breast analyses. This is done, according to equation 1.1, by projection of the estimated factor loadings of lung and breast factor 1 into the ovarian data set.

The first factor can be expected to represent the *in vitro* instantiation of key aspects of the projected E2F3 signature, suggesting that at least some, key aspects of E2F3 pathway activation across the cancer types is reflective of that seen in the experimental context. The consonance of the structure across the three cancers suggests a strong and meaningful biological underlying function is picked up in this component of the projected signature factor profile. This can be explored by simply looking at the names of genes identified as being highly loaded on factor 1 in each analysis.

If we look more closely at the three factors 1 in the different cancer types we see that, while the lung cancer data set yields a factor that highly loads substantially more genes than those discovered in breast and ovarian cancer, there is substantial overlap in highly loaded genes (Figure 1.2). If we look at the standardized expression levels of the genes in the intersection of these three factors in each of the three data sets, we see that there is a clear, shared pattern of expression across cancer types (Figure 1.3).

With the strongly conserved nature of the first factor across all three data sets, it is natural to ask whether there is a coherent biological structure one might attach to the associated genes. We used GATHER [23] to search for significant association between the 109 probes (representing 83 genes) in the intersection of the three factors and other known gene lists. All E2F's are

involved in cell cycle regulation, so it is of no surprise that this factor shows a strong association with the cell cycle pathway (38 of the 83 genes, GATHER Bayes' factor of 65). More remarkable is the fact that 50 of the 83 genes are in a list of 178 genes known to have binding sites for the transcription factor NF-Y in their promoter region [24]. There is strong evidence for the association between NF-Y and E2F; there is known to be a high correspondence between the presence of E2F3 and NF-Y binding sites in the promoter regions of many genes [25]. These two genes are also known to work in concert to regulate Cyclin B and the beginning of G_2/M phase of cell proliferation [20, 26]. Thus there is strong evidence that this factor represents co-regulation of the cell cycle by E2F3 and NF-Y.

1.4.2 Adenocarcinoma versus Squamous Cell Carcinoma

The lung cancer data set (from [3]) is comprised of some tissues from patients with squamous cell carcinoma, and some from patients with adenocarcinoma. These are significantly different types of non-small cell lung carcinoma (as defined histologically) and we expect that many pathways would show differential expression between these two groups. To explore this, we generated binary regression models with the lung cancer sub-type as response and the 15 estimate factors λ_i^* in lung cancer as candidate predictors. Bayesian model fitting and exploration of subset regressions on these factors used Shotgun Stochastic Search (SSS) of [27, 28] – following previous uses of SSS methodology in "large p" regression in genomics [29, 30, 31] – to search over models involving factors which distinguish between squamous cell and adenocarcinoma.

A model involving lung cancer E2F3 profiled factors 4 and 5 shows a clear ability to distinguish between the expression patterns of squamous cell carcinoma and adenocarcinoma (Figure 1.4). Given that there is no such distinction among the patients in the ovarian or breast cancer data sets, it is gratifying to note that the expression pattern exhibited by these genes is not conserved across the other two cancer data sets (Figure 1.5). This is a nice example of the use of factor profiling to identify clinically relevant predictive factor structure, one of the key reasons for interest in these models and the overall approach and is being used in other applications (e.g., [4, 13, 15]).

1.4.3 Survival and the E2F3 Pathway

The same general strategy was explored to assess whether the E2F3 profiled factors relate at all to tumor malignancy and aggressiveness, proxied by survival data available in all three cancer studies. Again we used SSS for regression model evaluation, this time using Weibull survival models that draw on the set of estimated factors as candidate predictors [27, 28]. From each of the three separate analyses, we then identified those factors with the highest posterior probabilities of being included in the survival regression across many

models explored and evaluated on subsets of factors. To exemplify the immediate clinical relevance of these "top survival factors" and their connections back to the underlying E2F3 pathway related biology, we mention here one of the top factors from each of the three analyses.

First, it is a measure of the importance of the E2F3/NF-Y pathway that the long term survival of breast cancer patients depends significantly on the E2F/NF-Y factor (Figure 1.6), the factor earlier discussed as being highly consonant across cancer types. Interestingly, from the survival regressions and follow-on exploration of survival curves, this pathway factor appears to be only mildly important in connection with ovarian cancer survival and really unimportant in lung cancer survival.

Survival in ovarian cancer is strongly and most heavily associated with ovarian factor 5 (Figure 1.7), though 1, 5, and 12 in combination produced the most probable individual survival model candidate in SSS. Factor 5 significantly loads on 129 probe ID's representing 113 genes. We again utilized GATHER to identify that 47 of these genes contain known binding sites for Transcription Factor DP-2 in their promoter region. While this factor does not share a strong correlation with any factor discovered in the breast or lung tissues, it is true that the expression pattern of the genes in the factor are conserved in the other data sets. Transcription Factor DP-2 is a known dimerization partner of the E2F family [32], and is important in cell cycle regulation via this interaction and perhaps other roles, and so this suggests a plausible interpretation of factor 5 as related to the interactions of E2Fs with DP-2. Again, this factor naturally emerges in the factor analysis as reflecting key aspects of variation of genes in the E2F3 related pathways, and this very strong linkage to survival outcomes is novel and a starting point for further biological investigation.

The most probable Weibull survival model in the lung cancer analysis involved lung cancer factor 9, and some of the strength of association of factor 9 is illustrated in Figure 1.8. This factor contains significant 80 probes representing 66 genes. Of these, 44 contain a known binding motif for E74-like factor 1 (ELF1) [33], a gene known to be important in the interleukin-2 immune response pathway. Additionally, ELF1 binds to the key Retinoblastoma protein [34]. Rb is a central checkpoint in the Rb/E2F network controlling cell proliferation, and is also bound by the members of the E2F family [19, 21]. This factor seems specific to lung cancer, and the genes it involves do not appear to show consistent expression in either breast or ovarian samples. A possible explanation of this is that the lung tumor tissue may have a higher level of invasion by immune cells than the other tissues. This possibility is corroborated by factor 8, which shows a high degree of relatedness (GATHER Bayes factor >18) to the biologically annotated pathways representing *"response to biotic stimulus", "defense response"* and *"immune response"* as calculated by GATHER. If we examine the expression patterns of lung factor 8 genes in the other two tumor tissue types, we find that the signature is visibly preserved, but the pattern is weaker and contains a higher noise level than in

lung cancers.

1.5 Closing Comments

Much recent and current expression genomics research has been devoted to the discovery of lists of genes showing differential expression across some known phenotypes, or that demonstrate an ability to stratify patients into different risk groups. While studies of this type have been shown to be useful in many applications, they have typically relied on the use of clustering and other simple methods to make the transition between *in vitro* and *in vivo* studies. The overall framework of Bayesian factor regression modelling utilising sparsity priors, as implemented in the BFRM software and exemplified in a range of recent studies, provides a formal, encompassing framework for such trans-study analysis.

The *in vivo* factor model profiling of *in vitro* defined expression signatures of controlled biological perturbations or environmental changes is a powerful and statistically sound approach that we have further explored and exemplified in the current chapter. Multiple current studies in cancer genomics, and other areas of both basic and human disease related biology, are using this approach. The example of the E2F3 signature factor profiling across three distinct cancer types and sample data sets is a vivid illustration of the kinds of results that can be expected: factor model refinements of *in vitro* signatures define multiple factors that relate to underlying biological pathway interconnections, generating suggestions for interpretation of factors, biologically interesting contrasts across cancer types, and suggesting novel directions for functional evaluation as well as identifying clinically useful predictive factors as potential biomarkers of cancer subtypes, survival, and other outcomes. Critically, the methodological framework of Bayesian regression factor modelling under sparsity priors for both designed experiments and observational samples enables and drives the overall strategy.

Acknowledgements

We are grateful to Ashley Chi and Joe Nevins of Duke University for useful discussions. We acknowledge support of the National Science Foundation (grants DMS-0102227 and DMS-0342172) and the National Institutes of Health (grants NHLBI P01-HL-73042-02 and NCI U54-CA-112952-01). Any opinions, findings and conclusions or recommendations expressed in this work are those of the authors and do not necessarily reflect the views of the NSF or NIH.

References

[1] E. Huang, M. West and J.R. Nevins (2003). Gene expression phenotypes of oncogenic pathways. *Cell Cycle*, **2**, 415–417.

[2] E. Huang, S. Ishida, J. Pittman, H. Dressman, A. Bild, M. D'Amico, R. Pestell, M. West and J.R. Nevins (2003). Gene expression phenotypic models that predict the activity of oncogenic pathways. *Nature Genetics*, **34**, 226–230

[3] A.H. Bild, G. Yao, J.T. Chang, Q. Wang, A. Potti, D. Chasse, M. Joshi, D. Harpole, J.M. Lancaster, A. Berchuck, J.A. Olson, J.R. Marks, H.K. Dressman, M. West and J.R. Nevins (2006). Oncogenic pathway signatures in human cancers as a guide to targeted therapies. *Nature*, **439**, 53–357.

[4] J.L. Chen, J.E. Lucas, T. Schroeder, S. Mori, J.R. Nevins, M. Dewhirst, M. West J.T. Chi (2008). Genomic analysis of response to lactic acidosis in human cancers. *Submitted to PLoS Medicine.*

[5] C.M. Perou, T. Sorlie, M.B. Eisen, M. van de Rijn, S.S. Jeffrey, C.A. Rees, J.R. Pollack, D.T. Ross, H. Johnsen, L.A. Akslen, O. Fluge, A. Pergamenschikov, C. Williams, S.X. Zhu, P.E. Lnning, A.-L. Brresen-Dale, P.O. Brown, and D. Botstein (2000). Molecular portraits of human breast tumors. *Nature* **406**, 747–752.

[6] M. West, C. Blanchette, H. Dressman, E. Huang, S. Ishida, R.!Spang, H. Zuzan, J.R. Marks and J.R. Nevins (2001). Predicting the clinical status of human breast cancer utilizing gene expression profiles. *Proceedings of the National Academy of Sciences*, **98**, 11462-11467.

[7] E. Huang, M. West and J.R. Nevins (2002). Gene expression profiles and predicting clinical characteristics of breast cancer. *Hormone Research*, **58**, 55–73.

[8] E. Huang, S. Chen, H. Dressman, J. Pittman, M.H. Tsou, C.F. Horng, A. Bild, E.S. Iversen, M. Liao, C.M. Chen, M. West, J.R. Nevins and A.T. Huang (2003). Gene expression predictors of breast cancer outcomes. *The Lancet*, **361**, 1590–1596.

[9] J. Pittman, E. Huang, H. Dressman, C.F. Horng, S.H. Cheng, M.H. Tsou, C.M. Chen, A. Bild, E.S. Iversen, A.T. Huang, J.R. Nevins and M. West (2004). Integrated modeling of clinical and gene expression

0-8493-0052-5/00/$0.00+$.50
© 2001 by CRC Press LLC

information for personalized prediction of disease outcomes. *Proceedings of the National Academy of Sciences*, **101**, 8431–8436.

[10] D.M. Seo, T. Wang, H. Dressman, E.E. Herderick, E.S. Iversen, C. Dong, K. Vata, C.A. Milano, J.R. Nevins, J. Pittman, M. West and P.J. Goldschmidt-Clermont (2004). Gene expression phenotypes of atherosclerosis. *Arteriosclerosis, Thrombosis and Vascular Biology*, **24**, 1922–1927.

[11] L.D. Miller, J. Smeds, J. George, V.B. Vega, L. Vergara, A. Ploner, Y. Pawitan, P. Hall, S. Klaar, E.T. Liu and J. Bergh (2005). An expression signature for p53 status in human breast cancer predicts mutation status, transcriptional effects, and patient survival. *Proceedings of the National Academy of Sciences*, **102**, 13550–13555.

[12] J. Rich, B. Jones, C. Hans, R. McClendon, D. Bigner, A. Dobra, J.R. Nevins and M. West (2005). Gene expression profiling and graphical genetic markers glioblastoma survival. *Cancer Research*, **65**, 4051–4058.

[13] D.M. Seo, P.J. Goldschmidt-Clermont and M. West (2007). Of mice and men: Sparse statistical modelling in cardiovascular genomics. *Annals of Applied Statistics*, **1**, 152–178.

[14] J. Lucas, C.M. Carvalho, Q. Wang, A. Bild, J.R. Nevins and M. West (2006). Sparse statistical modelling in gene expression genomics. In *Bayesian Inference for Gene Expression and Proteomics*, (eds: M. Vannucci *et al*), Cambridge University Press, 155–176

[15] C.M. Carvalho, J. Lucas, Q. Wang, J. Chang, J.R. Nevins and M. West (2008). High-dimensional sparse factor modelling - Applications in gene expression genomics. *Journal of the American Statistical Association* (in press)

[16] Q. Wang, C. Carvalho, J.E. Lucas and M. West (2007). BFRM: Bayesian factor regression modelling. *Bulletin of the International Society for Bayesian Analysis*, **14**, 4–5.

[17] H. Lopes and M. West (2003). Bayesian model assessment in factor analysis. *Statistica Sinica*, **14**, 41–67.

[18] M. West (2003). Bayesian factor regression models in the "large p, small n" paradigm. In *Bayesian Statistics 7*, (J.M. Bernardo, M.J. Bayarri, J.O. Berger, A.P. Dawid, D. Heckerman, A.F.M. Smith and M. West, eds.), Oxford University Press, 723–732.

[19] J.W. Harbour and D.C. Dean (2000). The Rb/E2F pathway: Expanding roles and emerging paradigms. *Genes and Development* **14**(19), 2393–2409.

[20] W. Zhu, P.H. Giangrande and J.R. Nevins (2004). E2Fs link the control of G1/S and G2/M transcription. *EMBO Journal*, **23**, 4615–4626.

[21] J.R. Nevins (2001). The Rb/E2F pathway and cancer. *Human Molecular Genetics*, **10**, 699–703.

[22] H.K. Dressman, A. Berchuck, G. Chan, J. Zhai, A. Bild, R. Sayer, J. Cragun, J.P. Clarke, R. Whitaker, L.H. Li, J. Gray, J. Marks, G.S. Ginsburg, A. Potti, M. West, J.R. Nevins and J.M. Lancaster (2007). An integrated genomic-based approach to individualized treatment of patients with advanced-stage ovarian cancer. *Journal of Clinical Oncology*, **25**, 517–525.

[23] J.T. Chang and J.R. Nevins (2006). GATHER: A systems approach to interpreting genomic signatures. *Bioinformatics*, **22**, 2926–2933.

[24] R. Mantovani (1998). A survey of 178 NF-Y binding CCAAT boxes. *Nucleic Acids Research*, **26**(5), 1135–1143.

[25] Z. Zhu, J. Shendure and G.M. Church (2005). Discovering functional transcription-factor combinations in the human cell cycle. *Genome Research*, **15**, 848–855.

[26] Q. Hu, J-F. Lu, R. Luo, S. Sen and S. Maity (2006). Inhibition of CBF/NF-Y mediated transcription activation arrests cells at G_2/M phase and suppresses expression of genes activated at G_2/M phase of the cell cycle. *Nucleic Acids Research*, **34**, 6272–6285.

[27] C. Hans, A. Dobra and M. West (2007). Shotgun stochastic search in regression with many predictors. *Journal of the American Statistical Association*, **102**, 507–516.

[28] C. Hans, Q. Wang, A. Dobra and M. West (2007). SSS: High-dimensional Bayesian regression model search. *Bulletin of the International Society for Bayesian Analysis*, **14**, 8-9.

[29] J. Rich, B. Jones, C. Hans, E.S. Iversen, R. McClendon, A. Rasheed, D. Bigner, A. Dobra, H.K. Dressman, J.R. Nevins and M. West (2005). Gene expression profiling and genetic markers in glioblastoma survival *Cancer Research*, **65**, 4051–4058.

[30] H.K. Dressman, C. Hans, A. Bild, J. Olsen, E. Rosen, P.K. Marcom, V. Liotcheva, E. Jones, Z. Vujaskovic, J.R. Marks, M.W. Dewhirst, M. West, J.R. Nevins and K. Blackwell (2006). Gene expression profiles of multiple breast cancer phenotypes and response to neoadjuvant therapy. *Clinical Cancer Research*, **12**, 819–216.

[31] C. Hans and M. West (2006). High-dimensional regression in cancer genomics. *Bulletin of the International Society for Bayesian Analysis*, **13**, 2–3.

[32] K.T. Rogers, P.D.R. Higgins, M.M. Milla and R.S. Phillips (1996). DP-2, a heterodimeric partner of E2F: Identification and characterization of

DP-2 proteins expressed *in vivo* *Proceedings of the National Academy of Sciences*, **93**, 7594–7599.

[33] C. Nishiyama, K. Takahashi, M. Nishiyama, K. Okumura, C. Ra, Y. Ohtake and T. Yokota (2000). Splice isoforms of transcription factor Elf-1 affecting its regulatory RT function in transcription-molecular cloning of rat Elf-1. *Bioscience, Biotechnology, and Biochemistry*, **64**, 2601–2607.

[34] C.Y. Wang, B. Petryniak, C.B. Thompson, W.G. Kaelin and J.M. Leiden (1993). Regulation of the Ets-related transcription factor Elf-1 by binding to the retinoblastoma protein *Science* **260**, 1330–1335.

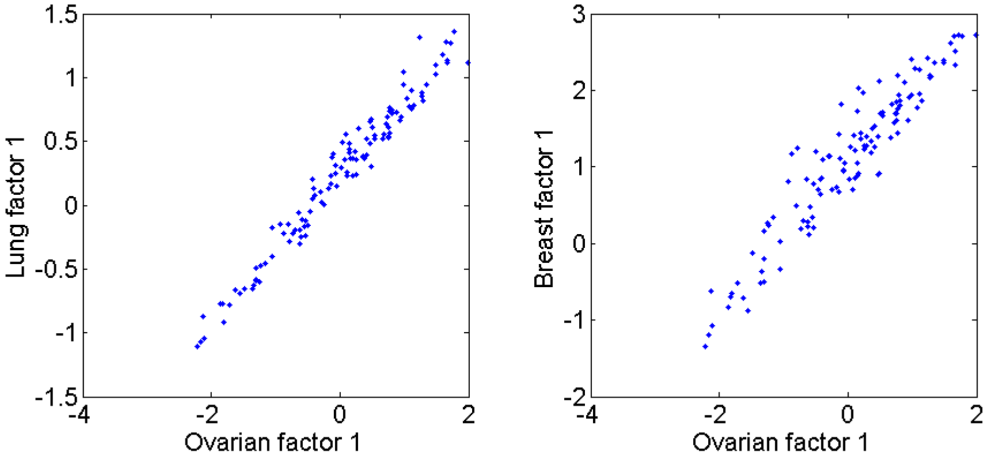

FIGURE 1.1: Scatter plot of estimated values $\lambda^*_{1,i}$ in the ovarian cancer data set (x-axis) against scores on the ovarian tumors of the lung and breast cancer factors 1; these two sets of scores are constructed by projection of the estimated factor loadings of lung and breast factor 1 into the ovarian data set, as described in section 1.2.3.

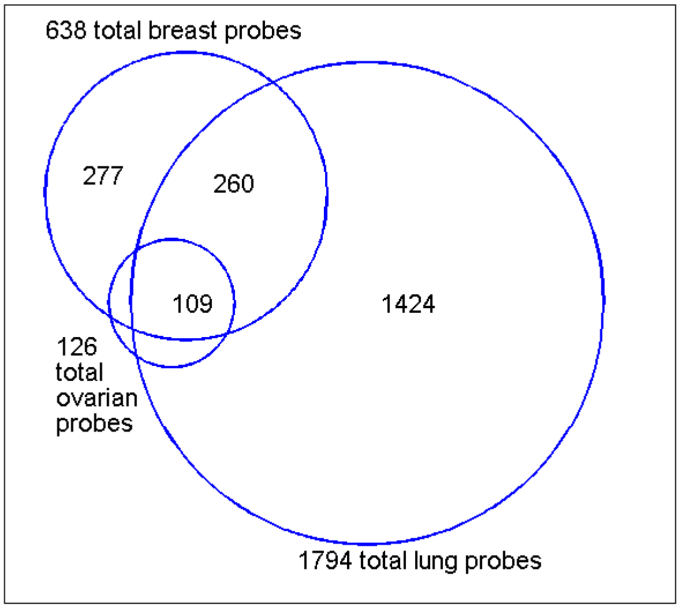

FIGURE 1.2: A Venn diagram depicting intersections of the gene lists of factor 1 from breast, lung, and ovarian cancers (total number of genes in each factor listed outside the circles, the number of genes in each intersection listed inside). Although the ovarian cancer factor 1 significantly involves a smaller number of genes that the other cancers, almost all of these (109 of 124) are common with those in the lung and breast factors.

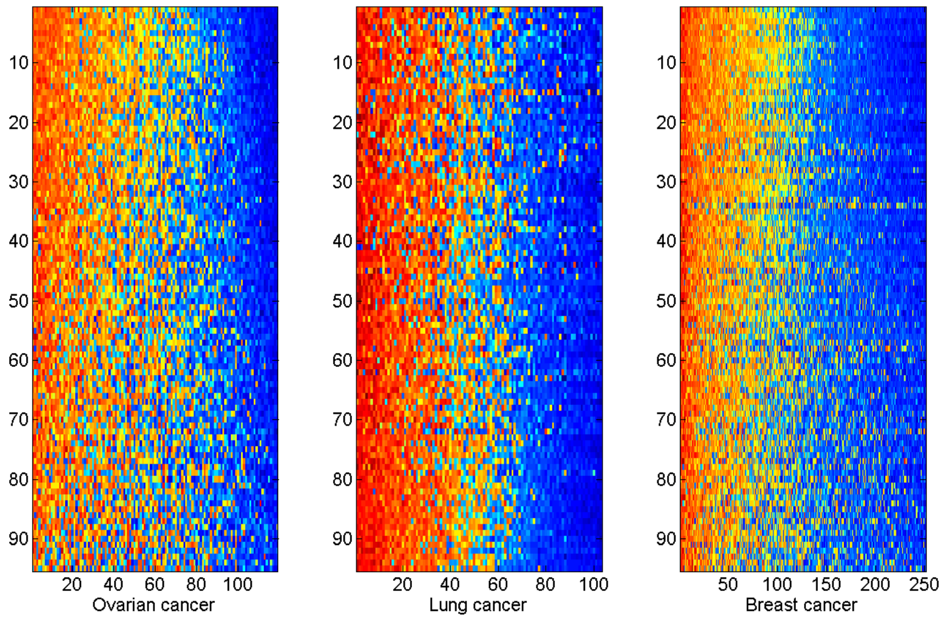

FIGURE 1.3: Patterns of expression of the 109 genes in the intersection group in Figure 1.2. The genes are ordered the same in the three heat-map images, and the patterns of expression across samples are evidently preserved across genes in this set.

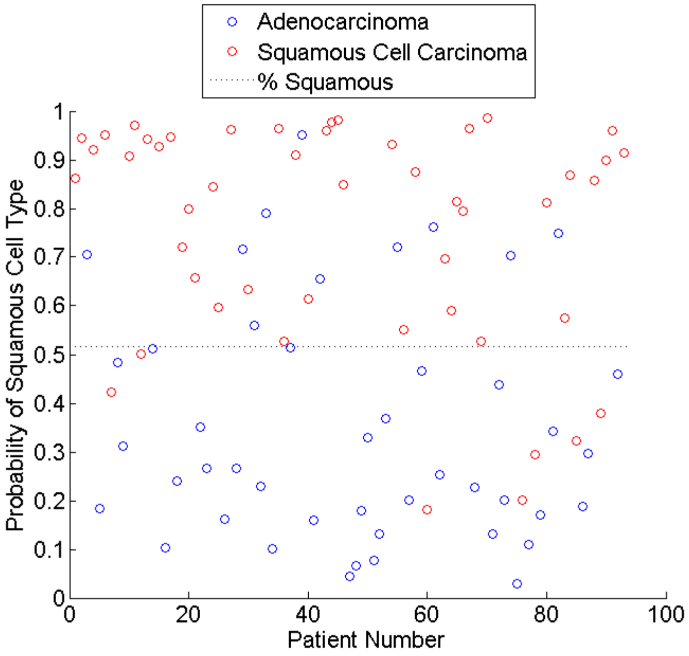

FIGURE 1.4: Factors 4 and 5 from the E2F3 signature factor profile in the lung cancer data set show a clear ability to distinguish between adenocarcinoma and squamous cell carcinoma. The figure displays probabilities of squamous versus adenocarcinoma from binary regression models identified using SSS.

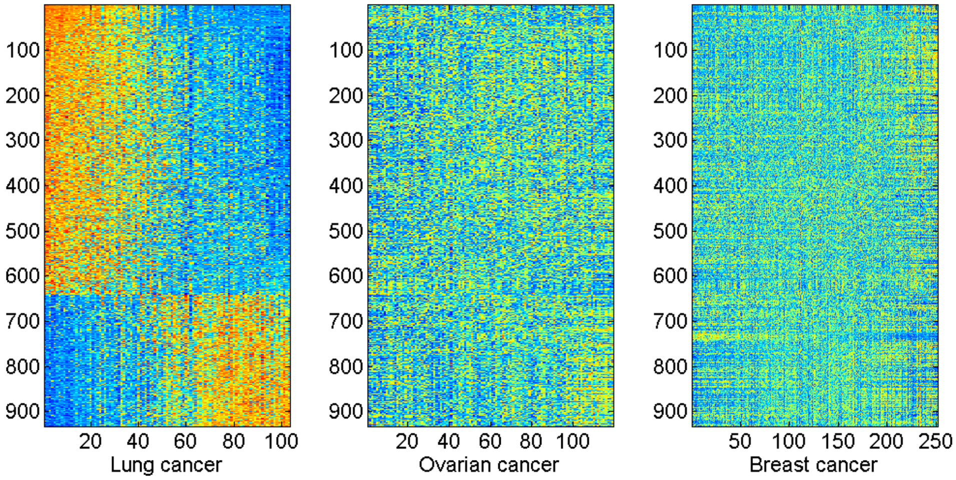

FIGURE 1.5: Patterns of expression of the genes in factor 4 in lung cancer. This factor shows differential expression of 350 genes that distinguish between squamous cell carcinoma and adenocarcinoma. It is therefore appropriate that the pattern of expression is clearly not conserved in the ovarian and breast cancers.

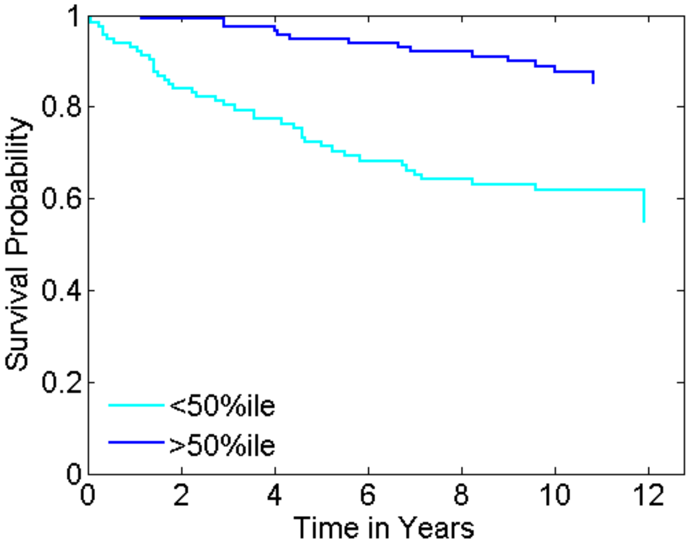

FIGURE 1.6: Kaplan-Meyer curves representing survival in breast cancer patients with above/below median scores for the E2F/NF-Y factor. Suppression of the E2F/NF-Y pathway appears to be associated with the decreased probability of long term survival.

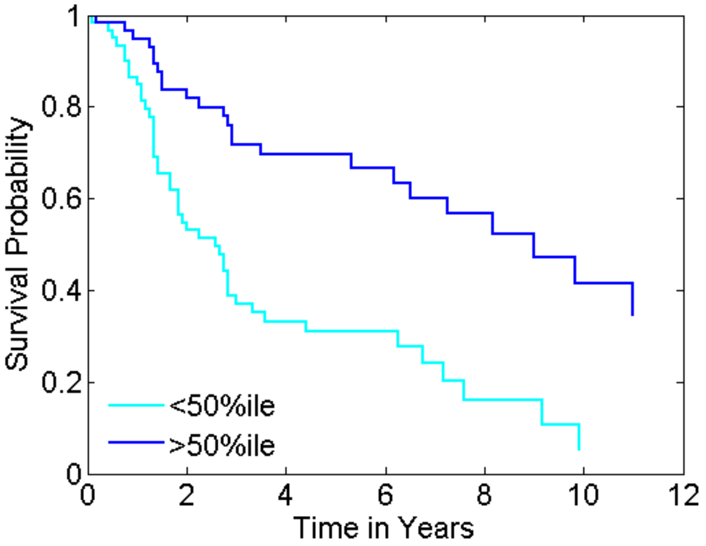

FIGURE 1.7: Kaplan-Meyer curves representing survival in ovarian cancer patients with above/below median scores for the E2F/TFDP2 factor. Suppression of the E2F/TFDP2 pathway appears to be associated with decreased probability of long term survival.

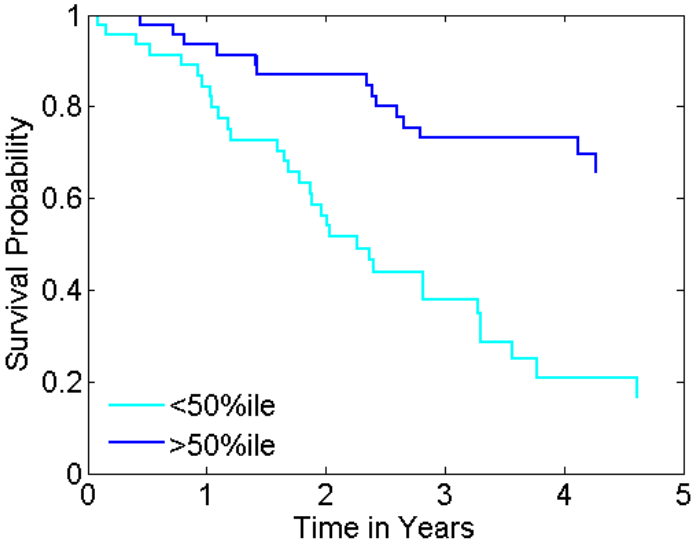

FIGURE 1.8: Kaplan-Meyer curves representing survival in lung cancer patients with above/below median scores for the E2F/ELF1 factor. Suppression of the E2F/ELF1 pathway appears to be associated with decreased probability of long term survival.
